# Redirecting microglia phenotype via inhibition of NFAT1 ameliorates deficits in mouse model of synucleinopathies

**DOI:** 10.1038/s12276-025-01564-4

**Published:** 2025-11-17

**Authors:** Michiyo Iba, Yeon-Joo Lee, Somin Kwon, Liam Horan-Portelance, Katherine Chang, Minhyung Kim, Natalie Landeck, Robert A. Rissman, Seung-Jae Lee, Sungyong You, Changyoun Kim

**Affiliations:** 1 Cell Biology and Gene Expression Section, Laboratory of Neurogenetics, National Institute on Aging, National Institutes of Health, Bethesda, MD USA; 2 Departments of Urology and Computational Biomedicine, Cedars–Sinai Medical Center, Los Angeles, CA USA; 3 Internal Medicine Residency Program, Walter Reed National Military Medical Center, Bethesda, MD USA; 4 Neurodegenerative Diseases Research Unit, National Institute of Neurological Disorders and Stroke, National Institutes of Health, Bethesda, MD USA; 5 Department Neurosciences, School of Medicine, University of California, San Diego, La Jolla, CA USA; 6 Department of Biomedical Sciences, Neuroscience Research Institute and Department of Medicine, Seoul National University College of Medicine, Seoul, Republic of Korea; 7 Samuel Oschin Comprehensive Cancer Institute, Cedars−Sinai Medical Center, Los Angeles, CA USA

**Keywords:** Neuroimmunology, Parkinson's disease

## Abstract

The abnormal deposition of α-synuclein (α-syn) and neuroinflammation are key features of synucleinopathies. We recently demonstrated that leucine-rich repeat kinase 2 (LRRK2) and nuclear factor of activated T cells 1 (NFAT1) modulate the neurotoxic inflammation in synucleinopathies mediated by microglia. Therefore, we hypothesized that targeting NFAT1 might ameliorate the microglial neurotoxicity in synucleinopathies. Here we utilized 11R-VIVIT, an NFAT1 inhibitory peptide, in in vivo, ex vivo and in vitro synucleinopathy models to evaluate the effects of NFAT1 inhibition to test this hypothesis. The microglia in synucleinopathy mouse models become excessively activated due to chronic disease conditions, thereby increasing the expressions of proinflammatory cytokines in these cells and decreasing the expressions of genes associated with microglial mobility and phagocytosis, strongly associated with neurodegeneration and pathogenic α-syn deposition. However, we observed that the inhibition of NFAT1 decreased the microglial neuroinflammation, thereby ameliorating neurodegeneration and α-syn neuropathology in vivo. Furthermore, the comprehensive in vivo transcriptomic analysis of the microglia revealed that the inhibition of NFAT1 restored their mobility and phagocytic abilities via upregulations of related genes. Our study proposes that the inhibition of NFAT1 redirects the excessively activated microglia to active healthy microglia, thereby reducing synucleinopathy neurotoxicity.

## NFAT1 inhibition reduces microglial neurotoxicity in synucleinopathies

Synucleinopathies, such as dementia with Lewy bodies and Parkinson’s disease, are neurodegenerative diseases characterized by the pathogenic accumulation of the protein α-synuclein. This pathogenic α-synuclein can spread between cells, promoting neurodegeneration and neuroinflammation. Microglia are the brain’s resident immune cells, which can become excessively activated in the synucleinopathy mouse model due to chronic disease conditions. This activation is associated with the increased expression of neurotoxic cytokines and the decreased expression of genes related to mobility and phagocytosis. NFAT1 is an immune-modulatory protein that plays a key role in microglia-mediated neuroinflammation. Researchers inhibited NFAT1 by the inhibitory peptide 11R-VIVIT in a mouse model of synucleinopathy and demonstrated that NFAT1 inhibition reduced neurodegeneration, neuroinflammation and α-synuclein neuropathology. The study suggests that targeting NFAT1 could be a promising therapeutic approach for treating synucleinopathies by restoring microglial function and protecting neurons.

This summary was initially drafted using artificial intelligence, then revised and fact-checked by the author.

## Introduction

Synucleinopathies are a group of age-related neurodegenerative diseases that include Parkinson’s disease (PD), dementia with Lewy bodies (DLB) and multiple system atrophy^1–3^. The abnormal depositions of α-synuclein (α-syn), called Lewy bodies and Lewy neurites, are representative pathological features strongly associated with the pathogenesis of these diseases^2,3^. Recent studies have shown evidence of cell-autonomous neurotoxicity of α-syn depositions, such as the disruption of synaptic vesicle recycling, mitochondrial homeostasis and protein homeostasis^1,3^. However, a large body of evidence also demonstrates the non-cell-autonomous neurotoxic mechanisms of α-syn deposition in these diseases, such as the pathogenic neuron-to-neuron and neuron-to-glial α-syn transmission^4–9^.

Neuroinflammation is another key pathologic feature of synucleinopathies and plays a critical role in the pathogenesis of neurodegeneration^7^. Although the mechanisms of neuroinflammation in synucleinopathies are not fully understood, the deposition of α-syn has been known to be strongly associated with neuroinflammation^10,11^. For example, we have demonstrated that neuron-released oligomeric forms of α-syn can induce microglial inflammatory responses through Toll-like receptor 2 (TLR2), thereby inducing the production of neurotoxic proinflammatory cytokine/chemokines, intracellular reactive oxygen species and nitric oxide^4,8,12^. TLR2 belongs to a family of pattern recognition receptors that has been known to play critical roles in innate immune responses^13–15^. Although TLR2 is mainly expressed in innate immune cells, it is also expressed in glial and neuronal cell populations within the central nervous system^16–18^. Furthermore, TLR2 has been known to be associated with multiple neurodegenerative diseases such as synucleinopathies and Alzheimer’s disease (AD). For example, TLR2 nucleotide polymorphisms are associated with patients with PD and AD^19–22^, along with the upregulation of TLR2 in the disease-affected brains of these patients^23–26^. Therefore, targeting TLR2 has been considered a reasonable therapeutic approach for many neurodegenerative disorders, including synucleinopathies^12^. Once activated, TLR2 induces a series of inflammatory responses in microglia via the activation of nuclear factor-κB (NFκB) and downstream signaling cascades^16^. However, the microglial TLR2–NFκB signaling cascade plays an important role in homeostasis in the central nervous system, such as producing neurotrophic factors^27,28^. Therefore, targeting TLR2, the most upstream element of this pathway, may not be an optimal therapeutic strategy to treat the disease, as it is likely to cause unexpected off-target effects.

We recently demonstrated the activation of an NFκB-independent signaling cascade in TLR2-stimulated microglia via leucine-rich repeat kinase 2 (LRRK2)^29^. In the study, we also verified that nuclear factor of activated T cell 1 (NFAT1) is a kinase substrate of LRRK2 in microglia, thereby mediating TLR2–LRRK2 signaling in the cells^29^. Given that the activation of the LRRK2 and NFAT1 signaling cascade plays a critical role in neuroinflammation and neurodegeneration of synucleinopathies^29^, we hypothesized that the selective inhibition of the most downstream component of the cascade, NFAT1, would rescue the overall burden of the pathologies without off-target effects of upstream target inhibition, such as TLR2. To address this hypothesis, we used a synucleinopathy mouse model, which overexpresses human wild-type α-syn under the murine Thy1 promotor, and administrated 11R-VIVIT, a selective cell-permeable NFAT1 inhibitor. This mouse model is known to mimic the neurodegenerative aspects of synucleinopathies, including α-syn neuropathology, neuroinflammation, neurodegeneration and behavioral deficits^30,31^. We selected 11R-VIVIT as a tool peptide among NFAT inhibitors owing to its selectivity and permeability to the blood–brain barrier (BBB)^27^. We found that 11R-VIVIT is capable of decreasing neuroinflammation and attenuating neuropathology and behavioral phenotypes via redirecting microglial phenotypes in the synucleinopathy model.

## Materials and methods

### Antibodies and chemicals

The antibodies used for the current study are summarized in Supplementary Table 1. The cell-permeable NFAT inhibitor (11R-VIVIT) was purchased from Tocris Bioscience. FluoSpheres (0.2 μm, red fluorescent) and human LRRK2 recombinant protein were obtained from Thermo Fisher Scientific. The human NFAT1 recombinant protein was purchased from ORIGENE. The lipopolysaccharide (LPS) was purchased from Sigma-Aldrich.

### Administration of NFAT1 inhibitor

Age-matched nontransgenic (non-TG) and synucleinopathy transgenic mice (α-syn-tg, line 61, mThy1 promoter)^30^ were injected intraperitoneally with either a control vehicle (saline) or an NFAT1 inhibitor (1 mg kg^-1^, 11R-VIVIT) 3 days per week for 5 weeks (Supplementary Table 2). We conducted three sets of animal experiments (for cohort 1, ~8–9 months old, total *n* = 20; for cohort 2, 3 months old, total *n* = 16; for cohort 3, ~3–4 months old, total *n* = 32). We used cohort 1 for immunohistochemical and biochemical analysis. For this, the mice brains were collected after the behavior test. The left hemispheres were snap-frozen and stored at −70 °C for biochemical analysis, and the right hemispheres were fixed with 4% paraformaldehyde for neuropathology analysis. The second cohort was used for microglial transcriptome analysis. We isolated the microglia from the brains for the RNA sequencing (RNA-seq) analysis. The third cohort was used for additional microglial transcriptome analysis, cytosolic-nuclear NFAT1 analysis in the brain and peripheral T cell profiling. All animal experiments were performed in accordance with protocol 463-LNG-2018, 463-LNG-2021 and 463-LNG-2024, approved by the institutional animal care and use committees of the NIH.

### Immunohistochemistry, immunofluorescence labeling and image analysis

The primary microglia were seeded onto poly-ᴅ-lysine (PDL)-coated cover slips, and blind-coded vibratome brain sections were incubated with various primary antibodies at 4 °C overnight. To detect proteinase K (PK)-resistant α-syn aggregates, brain sections were incubated with PK (10 μg/ml) for 8 min as previously described^31^. For immunofluorescence labeling, the brain sections and cover slips were incubated with fluorescein isothiocyanate and Texas Red secondary antibodies. To determine the level of NFAT1 in the cells, the sections were double-immunolabeled with Iba-1, NeuN or GFAP. The immunofluorescence was imaged with a Zeiss 63× (numerical aperture 1.4, Zeiss) objective on an Axiovert 35 microscope with an attached MRC 1023 LSCm system. For immunohistochemistry, the brain sections were incubated with biotinylated secondary antibodies and reacted with 3,3′-diaminobenzidine. The immunohistochemistry sections were scanned with NanoZoomer slide scanner (Hamamatsu) and the image files were processed utilizing QuPath software (v0.4.3)^32^. The numbers of Iba-1-positive, NeuN-positive, TH-positive, phosphor-α-syn-positive and nucleus NFAT1-positive cells were determined using QuPath’s built-in ‘positive cell detection’ per each field (400 μm × 400 μm) of the selected brain area. The immunoreactivity levels against NFAT1, GFAP, TH and α-syn were determined using QuPath’s built-in ‘pixel classification’ per field (400 μm × 400 μm) of the selected brain area.

### Behavioral test

After 4 weeks of injection, the animals were assessed for behavior. The motor coordination and motor learning were examined by rotarod analysis (IITC Life Science). The procedure for rotarod analysis is previously described^29^, and all data obtained from cohorts 1, 2 and 3 were combined. In brief, the rotation speed of the rod was gradually increased from 4 to 40 rpm over 5 min. The final rpm, rotarod time and distance were analyzed five times a day for a total of 4 days. The motor function was analyzed by the wire hang test, and the latency of the mice to fall was analyzed. The general and gross locomotor activity were determined by open field analysis. The total activity, ambulatory time and horizontal/vertical distances were determined by the Flex field photobeam activity system (San Diego Instruments).

### Detection of the tagged peptides in the brain

The endotoxin-removed 11R-VIVIT-V5 (RRRRRRRRRRRGGGMAGPHPVIVITGPHEEGKPIPNPLLGLDST) and TP10-V5 (Transportan 10-V5, AGYLLGKINLKALAALAKKILGKPIPNPLLGLDST) peptides were generated by GenScript. TP10 peptide was used as a control because it is known that peripherally administrated TP10 peptide does not cross the BBB in rodent model^28^. Eight-month-old non-TG mice were injected intraperitoneally with either the control vehicle (saline, *n* = 4), TP10-V5 (*n* = 4, 1 mg/kg) or 11R-VIVIT-V5 (*n* = 5, 1 mg/kg) daily for 3 days. One hour post final injection, the mice were perfused, and the brains were collected and separated into cytosolic and particulate fractions.

The brain infiltrated peptides were determined by the detection of the V5 tag in the brain homogenates utilizing the SimpleStep Enzyme-Linked Immunosorbent Assay (ELISA) Custom ELISA Kit (Abcam). In brief, monoclonal anti-V5 was used as the capture antibody, and polyclonal anti-V5 was used as the detection antibody. The brain cytosolic proteins (100 μg) were loaded onto a 96-well plate, followed by incubation with capture and detection antibodies (Supplementary Fig. 1a). The level of V5 was measured by SpectraMax M5e microplate reader (Molecular Devices).

### Nuclear and cytoplasmic extractions

Nuclear and cytoplasmic extractions were isolated from mouse brains utilizing the Cytoplasmic and Nuclear Protein Extraction Kit (Boster) and from primary microglia utilizing the NE-PER Nuclear and Cytoplasmic Extraction Reagents (Thermo Fisher Scientific) according to manufacturer instruction.

### In vivo microglia phenotype analysis

The blinded vibratome brain sections (40 μm) were immunostained with anti-Iba-1, and *Z*-stack images were taken with a Zeiss 780 confocal microscope (Zeiss). The background-adjusted images were imported into Bitplane Imaris 10.0.0. The surface reconstruction was performed with a surface detail of 0.2 μm and absolute threshold setting. The seed points were set at 10 μm. After surface reconstruction, cells touching the edge of the image were manually removed. Next, the images were masked using the surface reconstruction of the remaining microglia. Finally, the filaments were constructed using the filament tracer mode with the following settings: estimated largest diameter, 10.4 μm; seed point diameter, 0.6 μm; seed point threshold, 141; diameter of sphere region, 15. Following the filament tracing, the filaments were manually edited to separate the adjacent cells, and improperly traced filaments were deleted. For quantification, the 15 highest values were selected from each animal. The representative images were taken at 63 × magnification.

### Microglia isolation from adult brain

Following the treatment, the mouse microglia were isolated from the brain utilizing the Adult Brain Dissociation Kit (Miltenyi Biotec) according to the manufacturer’s instruction^33^. In brief, enzyme-mixed chopped brains were dissociated by gentleMACS Octo Dissociator with Heaters (Miltenyi Biotec) for 30 min. The brain homogenates were filtered with a 70-μm strainer and incubated with CD11b magnetic beads (Miltenyi Biotec) at 4 °C. After a 15-min incubation, the beads were rinsed with MACS buffer (Miltenyi Biotec), and microglia were eluted from the brain by pressure.

### Microglial transcriptome data analysis

The total RNA was isolated from microglia extracted from adult mice (cohorts 2 and 3), and RNA-seq was performed by Psomagen. For the bulk RNA-seq analysis, a total 16 mice were used; non-TG + saline (*n* = 4), non-TG + 11R-VIVIT (*n* = 4), α-syn-tg + saline (*n* = 4) and α-syn-tg + 11R-VIVIT (*n* = 4). For the RNA-seq data analysis, the quality of sequence reads was assessed using the FastQC tool (v0.11.9)^34^. Trim Galore (v 0.6.4_dev)^35^ was used to remove adapters from paired-end sequencing reads. The alignment of trimmed reads and transcript quantification was performed with mouse reference genome (mm10) using STAR aligner (v2.7.5c)^36^ and RSEM (v1.3.1)^37^ software. The genes were filtered out and excluded from the differential analysis if raw read counts were less than 10 in a sample. The raw data was normalized using the trimmed mean of M values (TMM) normalization procedure^38^. The raw expression levels were log_2_-transformed. The differentially expressed genes (DEGs) were determined using the hypothesis testing method as previously reported^39^. The DEGs were considered significant with the false discovery rate (FDR) <0.05 and absolute log_2_(fold change) ≥0.45.

### Functional enrichment analysis and network modeling

For gene ontology enrichment analysis, the Database for Annotation, Visualization and Integrated Discovery (DAVID, v6.8) was used^40^. DAVID provides web-based functional annotation tools for deriving biological meaning from large gene lists. To construct protein–protein interaction (PPI) networks, we used the genes with significant gene ontology terms (EASE score, a modified Fisher exact *P* value, <0.05). Among the genes enriched in migration, cell mobility, locomotion, phagocytosis, inflammatory response and regulation of immune system process, we indicated the genes reported to be associated with microglia^41–44^. The PPI between genes were assigned by interaction information from the Search Tool for the Retrieval of Interacting Genes/Proteins database (STRING, v11.5)^45^. Cytoscape software (v3.8.2)^46^ was used to reconstruct and visualize the network model. The gene sets for each enrichment term also were defined as the gene signatures affected by the NFAT inhibitor. The gene sets for each enrichment term are presented in Supplementary Table 6. The *Z*-score method was used to identify the signature activities^47^. We validated the activities of gene signatures in the PD cohort (GSE182622)^48^. The normalized expression matrix was obtained from the NCBI GEO database (http://www.ncbi.nlm.gov/geo). GSE182622 contains RNA-seq in mDA neurons isolated by laser-capture microdissection from postmortem cases of PD. The neuromelanin-positive dopamine neurons were isolated from the postmortem human substantia nigra from control, PD and incidental Lewy body disease cases. Among them, we used patients with PD with Braak stages of I and IV.

### Primary cell cultures and treatments

The procedure for neonatal primary microglia culture has been described elsewhere^8^. In brief, meninge-removed wild-type mice cortices were broken into small pieces in Dulbecco’s modified Eagle medium F12 by pipetting and incubation with trypsin and DNase1. After 2-min centrifugation (500*g*), the cell pellets were resuspended with fresh media, filtered with 120-μm Nylon mesh filter and incubated in PDL-coated T75 flasks. After ~12–15 days of incubation, the primary microglia were isolated from shake flasks and seeded onto PDL-coated cover slips or cell culture wells. The next day, microglia were treated with α-syn conditioned medium (αSCM), β-galactosidase conditioned medium (LZCM) or LPS (0.2 μg/ml) in the presence or absence of NFAT inhibitor (1 μM, 11R-VIVIT).

The procedure for primary cortical neuronal culture has been previously described^8^. In brief, meninge-removed mouse cortices were incubated with papain solution (Hanks’ Balanced Salt Solution containing 10 U/ml papain, 0.2 mg/ml cysteine, 0.5 mM ethylenediaminetetraacetic acid, 1 mM CaCl_2_ and 0.003 N NaOH), followed by incubation with a trypsin inhibitor solution (MEM with Earl’s salts containing 5% fetal bovine serum, 2.5 mg/ml bovine serum albumin and 2.5 mg/ml trypsin inhibitor). The tissues were dissociated by pipetting and centrifuged for 1 min at 200*g* to pellet the cells. The pellet was resuspended with complete neurobasal media (2% B-27, 0.5 mM Glutamax-1) and anti-mitotic media (2 μg/ml 5-fluoro-2′deoxyuridine and 9.6 μg/ml uridine), seeded onto PDL/laminin-coated culture plates and incubated for 5 days.

### Immunoblot analysis

The cells were rinsed with ice-cold phosphate-buffered saline (PBS), then lysed with NuPAGE LDS sample buffer (Thermo Fisher Scientific) in the presence of protease and phosphatase inhibitors. Before separation, the cell lysates were briefly sonicated.

The human and mouse brain tissues were homogenated in platelet-derived growth factor lysis buffer (1% Triton X-100, 10% glycerol, 50 mM HEPES, 140 mM NaCl, 1 mM ethylenediaminetetraacetic acid, 1 mM Na_3_VO_4_, 20 mM β-glycerophosphate and protease–phosphatase inhibitors). After the removal of debris by centrifugation (1,000*g* for 5 min at 4 °C), the supernatants (whole brain lysate) were separated into cytoplasmic and particulate fractions (100,000*g* for 1 h at 4 °C). The whole brain lysates and cytoplasmic/particulate fractions were then lysed with NuPAGE LDS sample buffer.

The cells and brain lysates were separated with 4–12% Bis–Tris sodium dodecyl sulfate–polyacrylamide gel electrophoresis gels and transferred onto polyvinylidene fluoride membranes. The membranes were blocked with Odyssey Blocking Buffer (LI-COR Biosciences) and subsequently incubated with various primary and fluorescent secondary antibodies. The images were obtained by Odyssey CLx Imaging System (LI-COR Biosciences) and analyzed with Image studio software (LI-COR Biosciences).

### Quantitative PCR

The total RNA was extracted from microglial cells and mouse brains using the RNeasy mini kit and RNeasy universal kit (Qiagen), respectively. A total of 0.1–0.5 μg of RNA for microglia and 1 μg of RNA for mouse brains were reverse transcribed using SuperScript VILO cDNA synthesis kit (Thermo Fisher Scientific) according to the manufacturer’s instruction. The quantitative real-time PCR was performed using TaqMan Fast Advanced Master Mix (Thermo Fisher Scientific) and various gene-specific primers (Supplementary Table 3). The DNA amplification was measured by the StepOnePlus real-time PCR system (Thermo Fisher Scientific). The relative mRNA levels were calculated according to the 2^−ΔΔCt^ method^8^. All ΔCT values were normalized to β-actin and displayed as fold inductions.

### ELISA

To measure the microglial secretions of TNFα and IL-6, primary wild-type microglia were treated with either αSCM or LZCM in the presence or absence of 11R-VIVIT 30 min pretreatment. After a 6-h treatment, culture media was collected and centrifuged at 10,000*g* for 1 min to remove cell debris. The whole cell lysates and cytoplasmic/particulate fractions were utilized to determine the levels of internalized α-syn in primary wild type microglia. ELISA was conducted with the TNFα Mouse ELISA kit, IL-6 Mouse ELISA kit or α-syn Human ELISA kit (Thermo Fisher Scientific).

To determine Thr862-phosphorylated NFAT1, we utilized SimpleStep ELISA Custom ELISA kit (Abcam) according to the manufacturer’s instruction. In brief, anti-NFAT1 (Thermo Fisher Scientific) was used as the detection antibody, and anti-phosphor-NFAT1 (Thr862) (C9) (Generated by GenScript) was used as the capture antibody. The LRRK2 kinase-reacted NFAT1 samples were gradually loaded to a 96-well plate, followed by incubation with detection and capture antibodies.

The levels of TNFα, IL-6, human α-syn and phosphor-Thr862-NFAT1 were measured by SpectraMax M5e microplate reader (Molecular Devices).

### Preparation of MgCM

The preparation of microglial conditioned medium (MgCM) has been described elsewhere^29^. In brief, non-TG primary microglia were treated with either αSCM or LZCM in the presence or absence of NFAT inhibitor (11R-VIVIT) for 1 h. After washing four times, the cells were incubated with fresh complete neurobasal media for 6 h. The culture media was collected and centrifuged at 10,000*g* for 10 min to remove cell debris.

### Preparations of neuron-released and recombinant fibrillar α-syn

αSCM and control LZCM were obtained from α-syn-expressing and β-galactosidase-expressing differentiated SH-SY5Y neuronal cells, respectively. αSCM contains 1.06 ± 0.371 mg/ml of α-syn^8^, and approximately 100 ng/ml of α-syn was used to treat cells of the current study. α-syn fibrils were generated by endotoxin-free human recombinant α-syn. The procedures for preparing conditioned medium and α-syn fibrils have been previously described^49^.

### Neuronal toxicity analysis

The neuronal cytotoxicity analysis procedure has been previously described^29^. In brief, wild type primary mouse cortical neurons were treated with Dulbecco’s modified Eagle medium (control), Con-MgCM, 11R-MgCM, αS-MgCM, or αS-11R-MgCM for 18 h before cytotoxicity assay (Fig. 5m). The neuronal cytotoxicity was determined by two different methods, TC20 Automated Cell Counter (BIO-RAD) and CyQUANT cell proliferation assay (Thermo Fisher Scientific), according to the manufacturer’s instructions.

### Wound healing assay

The wound healing procedure has been previously described^50^. In brief, primary wild-type microglia were seeded onto a 24-well PDL-coated plate and pretreated with either control or LPS (0.2 μg/ml) for 24 h, and then, the surfaces of cell cultures were scratched with a 200-μl tip and the widths of scratched surfaces were measured at nine marked sites per sample. After washing four times, cells were incubated with α-syn fibrils (400 nM) in the presence or absence of NFAT inhibitor (1 μM). At 4 and 24 h of incubation, the recovery width of the scratched surfaces was remeasured at the nine marked sites for each independent experiment.

### Brain slice culture and ex vivo α-syn phagocytosis assay

The brain slices were prepared from wild-type pups between postnatal days 10–12. The mouse pups were decapitated, the brains extracted and immediately cooled in ice-cold artificial cerebrospinal fluid (125 mM NaCl, 2.5 mM KCl, 1.3 mM NaH_2_PO4, 25 mM NAHCO_3_, 2 mM CaCl_2_, 1 mM MgCl_2_ (Sigma-Aldrich), 25 mM glucose (Gibco), carbogen infused (95% oxygen, 5% CO_2_)). Then, the brains were mounted upright onto a clean Leica Vibratome (VT100 S) and cut into 300-μm-thick coronal sections while in ice-cold artificial cerebrospinal fluid under constant carbogen infusion. The collected brain slices were randomly plated onto a semiporous membrane insert (0.4-μm pore size (Millipore)) set into a sterile six-well plate. The slices were maintained at 37 °C and 5% CO_2_ in sterile filtered media (75% Basal Medium Eagle (Sigma-Aldrich), 25% heat-inactivated horse serum, 1 × B27 supplement, 1 × N2 supplement, 25 mM glucose, 1% GlutaMax and 1% Pen/Strep (Gibco)). After a day, the brain slices were incubated with either control or LPS (1 μg/ml) for 48 h. Then, the brain slices were exposed to human α-syn fibrils (500 nM) in the presence or absence of 11R-VIVIT (2 μM) for 4 h. The brain slices were gently washed three times with PBS and separated into cytosolic and particulate fractions for further analysis.

### Microglial phagocytosis assay

The primary wild-type microglia were seeded onto PDL-coated cover slips and pretreated with either control or LPS (0.2 μg/ml). After 24 h, the cells were treated with FluoSpheres (0.1%) in the presence or absence of the NFAT inhibitor (0.1 μM) for 1 and 4 h after pretreatment. The cells were then washed three times with PBS and fixed with 4% paraformaldehyde. The fixed cells were immunostained with anti-Iba-1 and analyzed with a Zeiss 63 × (Zeiss) objective on an Axiovert 35 microscope with an attached MRC 1023 LSCm system.

### In vitro LRRK2 kinase assay

The recombinant human NFAT1 protein was incubated with equal amounts of recombinant G2019S LRRK2^970–2527^ (5:1 molar ratio) in 1 × kinase buffer in the presence or absence of ATP (1 mM) for 30 min at 30 °C. To terminate the reactions, sodium dodecyl sulfate loading buffer (4×) was added to the samples. The phosphorylation of NFAT1 were determined by immunoblot analysis and ELISA.

### Human specimens, neuropathological evaluation and diagnostic criteria

The human specimens (9 age-matched controls and 12 PD/DLB cases) used in this study were obtained from the Alzheimer Disease Research Center at the University of California, San Diego (Supplementary Table 4). Diagnosis was based on initial clinical presentation with dementia followed by parkinsonism and presences of cortical and subcortical α-syn/Ubiquitin-positive Lewy bodies^51^. All brain tissues were donated with written informed consent from donors and their families, and all protocols were approved by the University of California, San Diego ethics committee.

### Flow cytometry

The cell suspensions from spleen and lymph nodes (6 × 10^6^ cells) were stimulated with phorbol 12-myristate 13-acetate (50 ng/ml) and ionomycin (1 μM) for 4 h. To inhibit the cytokine secretion, monensin (Sigma-Aldrich) was added during the final 2 h of stimulation at a final concentration of 3 μM. The cells were stained with fixable live/dead fluorescent Aqua dye (Invitrogen) according to the manufacturer’s instructions, followed by surface staining at 4 °C for 30 min with anti-CD4 APC-eFluor 780 (RM4-5, Invitrogen) and anti-CD8 Pacific Blue (5H10, Invitrogen). The cells were fixed with 4% paraformaldehyde for 10 min at room temperature and permeabilized with Cytofix/Cytoperm solution (BD Biosciences) for 45 min. The intracellular cytokine staining was performed with anti-IFN-γ PE (XMG1.2, BioLegend) and anti-IL-2 PE-Cyanine7 (JES6-5H4, Invitrogen) antibodies for 45 min at room temperature. The samples were acquired on a BD LSRII flow cytometer (BD Biosciences) and analyzed with FlowJo v10.9 software (BD Life Sciences).

### Statistical analysis

All data were presented as means ± SEM. The number of independent experiments in in vitro assays, human participants and mouse subjects are represented by *n*. The statistical analyses were performed using Prism 10 (GraphPad). The statistical analyses were conducted using unpaired *t*-test, one-way analysis of variance (ANOVA) followed by Tukey’s multiple comparison tests, two-way ANOVA followed by Tukey’s multiple comparison tests and three-way ANOVA with the Geisser–Greenhouse correction. No animal or sample was excluded from the analysis except in the wire hang analysis, which excluded mice according to body weight.

## Results

### Administration of cell-permeable NFAT1 inhibitor ameliorates deficits in mouse model of synucleinopathies

We first demonstrated the successful trafficking of intraperitoneally administrated NFAT1 inhibitory peptide (11R-VIVIT) to the brain of nontransgenic (non-TG) mice (Supplementary Fig. 1a, b). Age-matched non-TG mice were intraperitoneally injected with either saline, V5-tagged 11R-VIVIT (11R-VIVIT-V5) or V5-tagged transportan 10 peptide (TP10-V5) daily for 3 days, and the peptides that could cross the BBB were determined by detection of V5 in the cytosolic fraction of mouse brains (Supplementary Fig. 1a). TP10 peptide was used as a control as it has been known to be unable to cross the BBB when peripherally administered, whereas other peptides such as pVEC are immediately able to cross the BBB in a mouse model^28^. The level of V5 was significantly increased in the cytosolic fractions of 11R-VIVIT administrated mouse brain homogenates compared with saline-administrated non-TG mice, whereas it was not increased in TP10-V5-administrated mouse brain homogenates (Supplementary Fig. 1b).

**Fig. 1:**
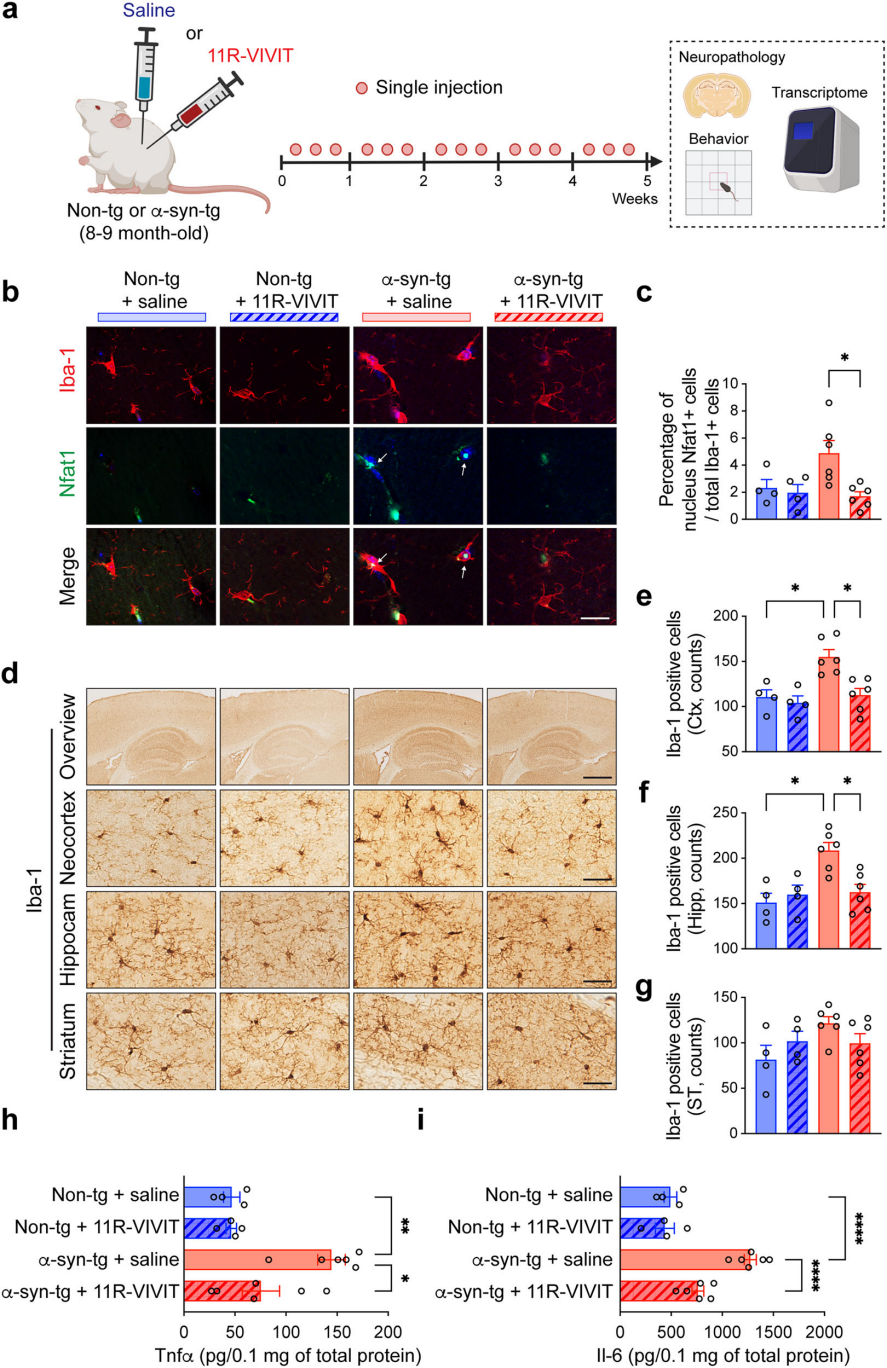
Administration of NFAT1 inhibitor reduces microglial activation in a synucleinopathy mouse model. **a** The experimental scheme; non-TG and α-syn-tg mice were administrated either saline or 11R-VIVIT three times per week intraperitoneally for 5 weeks. **b** The representative images from double-immunolabeling for Nfat1 and microglia marker Iba-1. Scale bar, 15 μm. The arrows indicate nuclear Nfat1. **c** The percentages of nucleus Nfat1-positive among Iba1-positive cells in the neocortex of the mice (*n* = 4 or 6 per group). The data are shown as means ± s.e.m. Two-way ANOVA and Tukey’s multiple comparison test, *P < 0.05. **d** The representative images from immunohistochemical staining of Iba-1 in the neocortex (Ctx), hippocampus (Hipp) and striatum (ST) of the mice. Scale bars, 250 μm (low magnification) and 25 μm (high magnification). **e**–**g** The numbers of Iba-1-positive cells in the neocortex (**e**), hippocampus (**f**) and striatum (**g**) (*n* = 4 or 6 per group). The data are shown as means ± s.e.m. Two-way ANOVA and Tukey’s multiple comparison test, *P < 0.05. **h**, **i** The levels of Tnfα (**h**) and Il-6 (**i**) in the whole mice brain homogenates determined by ELISA (*n* = 4 or 6 per group). The data are shown as means ± s.e.m. Two-way ANOVA and Tukey’s multiple comparison test, *P < 0.05, **P < 0.01, ****P < 0.0001.

We next administrated 11R-VIVIT to non-TG and α-syn-tg mice. Age-matched non-TG and α-syn-tg mice were administrated either saline or 11R-VIVIT (1 mg/kg) intraperitoneally three times per week (Fig. 1a). Aged α-syn-tg mice (8–9-month-old) with neuropathology and behavioral deficits were used to evaluate the effect of NFAT1 inhibition. After 5 weeks of injection, the mouse behavioral tests were performed before collecting the brains (Fig. 1a). We first determined the level of NFAT1 in the mouse model. The immunoreactivity of NFAT1 in α-syn-tg mice was increased in the hippocampus compared with non-TG mice and was decreased in the group administrated with 11R-VIVIT in both the neocortex and hippocampus relative to those treated with saline (Supplementary Fig. 2a–c). The 11R-VIVIT treatment also decreased NFAT1 in the neocortex of non-TG mice (Supplementary Fig. 2a, b). The level of striatal NFAT1 was not affected by the α-syn expression or NFAT1 inhibition (Supplementary Fig. 2d). The immunoblot analysis likewise demonstrated that the levels of total and activated (phosphor-S54 NFAT1) NFAT1 were increased in the brains of α-syn-tg mice compared with non-TG mice and were decreased by 11R-VIVIT administration (Supplementary Fig. 2e–h). Once activated, NFAT1 is known to translocate to the nucleus and induce the transcription of multiple immune-related genes^52,53^. Therefore, we also examined the level of nucleus-translocated NFAT1 by utilizing double immune-labeling analysis (Fig. 1b, c and Supplementary Fig. 3) and nucleus fractionation of the mouse brains (Supplementary Fig. 4). The percentages of nuclear NFAT1-positive microglia, astrocytes and neurons were decreased by the 11R-VIVIT treatment of the α-syn-tg mice (Fig. 1b, c and Supplementary Fig. 3a–d). Furthermore, the level of phosphor-S54 NFAT1 was increased in the nucleus fractions of α-syn-tg mice brains compared with non-TG mice, whereas it was significantly decreased by 11R-VIVIT administration (Supplementary Fig. 4a, c). The level of cytosolic phosphor-S54 NFAT1 was not altered by genotype or 11R-VIVIT administration (Supplementary Fig. 4a, b).

The number of Iba-1-positive microglia was increased in the neocortex and hippocampus of α-syn-tg mice compared with non-TG mice, whereas it was significantly decreased by 11R-VIVIT administration (Fig. 1d–f). However, it was not affected by the genotype or 11R-VIVIT administration in the striatum of the mice (Fig. 1d, g). The microglial phenotype was also analyzed utilizing Imaris (Supplementary Fig. 5a–c). As expected, compared with non-TG mice, the microglia of α-syn-tg mice had shorter and fewer branches (Supplementary Fig. 5a–c), though interestingly, the administration of 11R-VIVIT decreased the length and number of the microglial branches in both non-TG and α-syn-tg mice (Supplementary Fig. 5a–c). We next determined the levels of NFAT1-associated cytokines in mouse brain homogenates through ELISA analysis. We found that Tnfα and Il-6 levels were increased in the α-syn-tg compared with non-TG mice and decreased by NFAT1 inhibition (Fig. 1h, i). Along with microgliosis, astrogliosis was also increased in the neocortex and hippocampus of the α-syn-tg mice but was not significantly affected by the 11R-VIVIT administration (Supplementary Fig. 6a–c). Similar to striatal microgliosis, striatal astrogliosis was not affected by the genotype or 11R-VIVIT administration (Supplementary Fig. 6a, d).

**Fig. 2:**
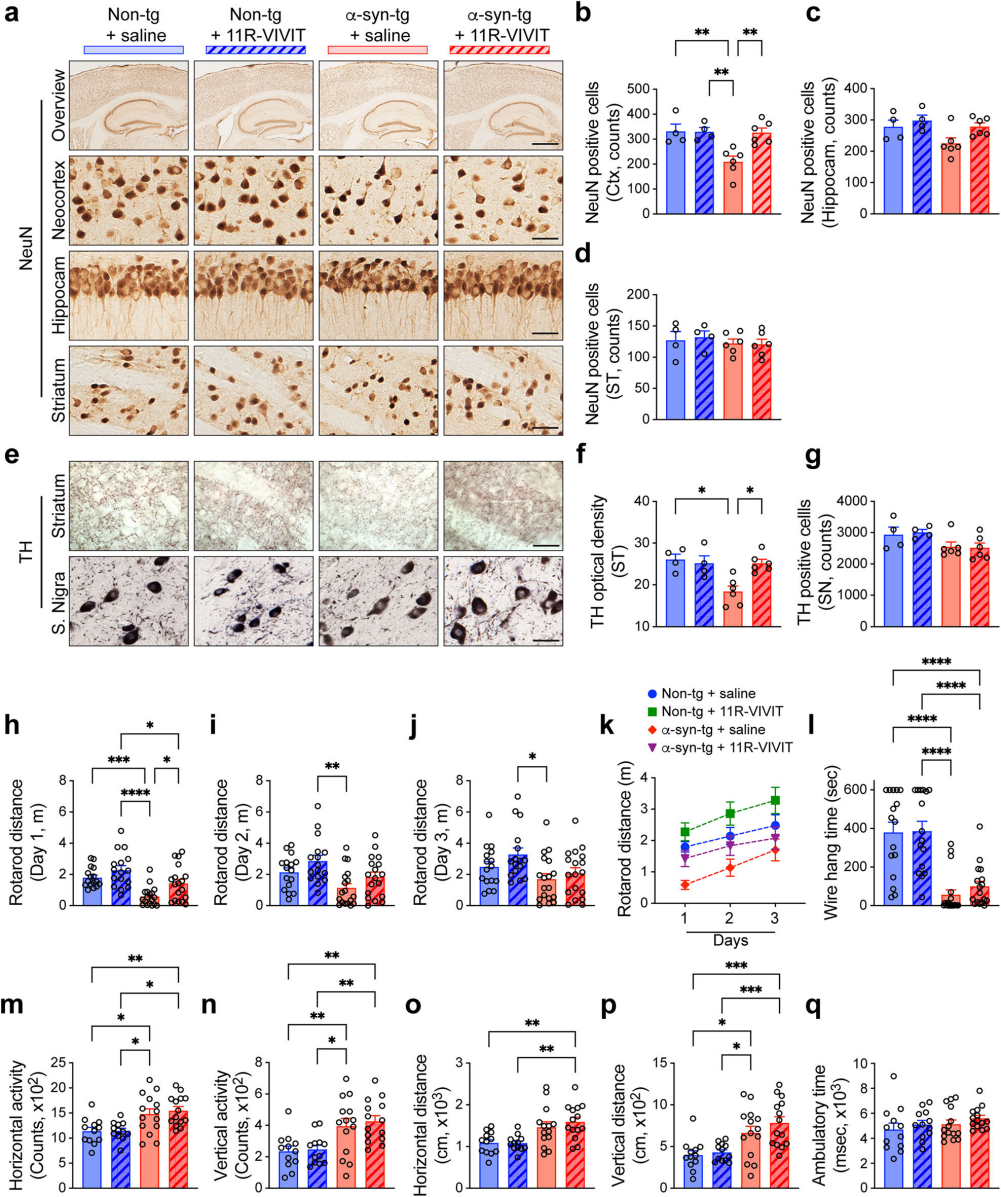
Inhibition of NFAT1 ameliorates neurodegeneration and behavioral deficits in a synucleinopathy mouse model. **a** The representative images from the immunohistochemical staining of NeuN in the neocortex, hippocampus and striatum of the mice. Scale bars, 250 μm (low magnification) and 25 μm (high magnification). **b**–**d** The numbers of NeuN-positive cells counted in the neocortex (Ctx) (**b**) hippocampus (Hipp) (**c**) and striatum (ST) (**d**) (*n* = 4 or 6 per group). The data are shown as means ± s.e.m. **P < 0.01, two-way ANOVA and Tukey’s multiple comparison test. **e** The representative images from the immunohistochemical staining of TH in the striatum and substantia nigra of the mice. Scale bar, 25 μm. **f** The level of TH was analyzed by optical density quantification in the striatum of the mice. **g** The number of TH-positive cells counted in the substantia nigra of the mice (*n* = 4 or 6 per group). The data are shown as means ± s.e.m. *P < 0.05, two-way ANOVA and Tukey’s multiple comparison test. **h**–**q** The behavioral analysis of the injected mice; motor learning and function were tested by rotarod.; average rotarod end distances from five trials for days 1 (**h**), 2 (**i**) and 3 (**j**) were recorded and analyzed (*n* = 16 or 18 per group), and the data are shown as means ± s.e.m., *P < 0.05, **P < 0.01, ***P < 0.001, ****P < 0.0001, two-way ANOVA and Tukey’s multiple comparison test; the distance records by day were displayed on a graph (**k**) and the data are shown as means ± s.e.m., three-way ANOVA with the Geisser–Greenhouse correction; the motor coordination and neuromuscular impairment were tested by the wire hang test (*n* = 16 or 18), and the data are shown as means ± s.e.m., ****P < 0.0001, two-way ANOVA and Tukey’s multiple comparison test (**l**) total horizontal (**m**) and vertical activities (**n**) total horizontal (**o**) and vertical distances (**p**) and total ambulatory time (**q**) were tested by open field test (*n* = 12 or 14 per group). The data are shown as means ± s.e.m. Two-way ANOVA and Tukey’s multiple comparison test, *P < 0.05, **P < 0.01, ***P < 0.001.

Consistent with the previous findings, neurodegeneration was also observed in the α-syn-tg mice^29,31^. Compared with the non-TG mice, the number of NeuN-positive neurons was decreased in the neocortex of α-syn-tg mice, which was rescued by the administration of 11R-VIVIT (Fig. 2a, b). The numbers of NeuN-positive hippocampal and striatal neurons were not affected by the genotype or 11R-VIVIT treatment in animals (Fig. 2a, c, d). Neuropil of tyrosine hydroxylase (TH)-positive neurons was decreased in the striatum of α-syn-tg mice compared with non-TG mice and rescued by 11R-VIVIT treatment (Fig. 2e, f). The numbers of TH-positive substantia nigral neurons were not affected by genotype or 11R-VIVIT treatment either (Fig. 2e, g). We next investigated the expressions of neuronal presynaptic genes synapsin I (Syn1), synaptojanin 1 (SynJ1), synaptosomal-associated protein 25k (Snap25) and synaptophysin (SynP) and post-synaptic genes activity regulated cytoskeleton associated protein (Arc) and neuroligin 1 (Nlgn1) to evaluate neuronal activity in the mice (Supplementary Fig. 7a–f). Consistent with our previous report^54^, among these genes, the expression of Snap25 was decreased in α-syn-tg compared with non-TG mice, which was rescued by 11R-VIVIT treatment in animals (Supplementary Fig. 7a). The expression of Arc was also decreased in α-syn-tg mice and rescued by NFAT1 inhibition in animals (Supplementary Fig. 7b). However, the expression of Syn1 was increased in α-syn-tg compared with non-TG mice, and 11R-VIVIT treatment induced further increases in both non-TG and α-syn-tg mice (Supplementary Fig. 7c). Lastly, the expression of SynJ1, SynP and Nlgn1 were not affected by genotype or 11R-VIVIT treatment in both sets of mice (Supplementary Fig. 7d–f).

We next examined whether administrating the NFAT1 inhibitor to α-syn-tg mice could ameliorate the behavioral deficits seen in this model^9^. The behavior was assessed utilizing the rotarod (Fig. 2h–k), wire hang (Fig. 2l) and open field tests (Fig. 2m–q). The administration of the NFAT1 inhibitor restored motor behavioral impairment in α-syn-tg mice on the first day of analysis (Fig. 2h). However, this was not observed on the second or third days of analysis (Fig. 2i, j). The effect of genotype, 11R-VIVT treatment and motor learning did not show a significant three-way interaction (*F*(2,128) = 1.306, *P* = 0.655) (Fig. 2k). Motor neuromuscular impairment was not significantly improved by 11R-VIVIT treatment in α-syn-tg mice (Fig. 2l). Consistent with previous reports^54^, the horizontal and vertical activities of the open field test were significantly increased in α-syn-tg mice compared with non-TG mice (Fig. 2m–p). However, these were not affected by NFAT1 inhibition in either non-TG or α-syn-tg mice (Fig. 2m–p). Ambulatory time was not altered by genotype or NFAT1 inhibition in the mice (Fig. 2q).

**Fig. 3:**
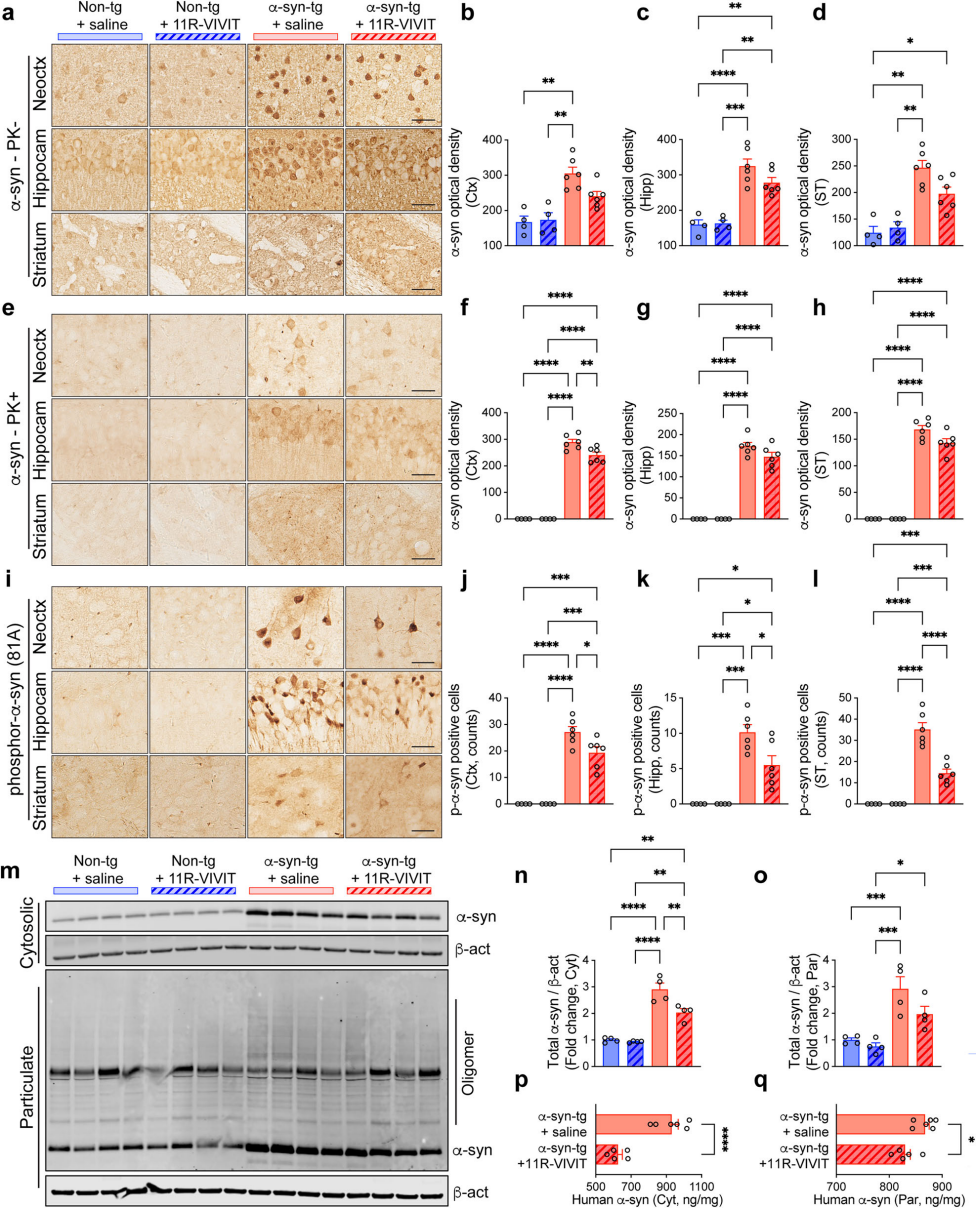
Reduction of α-syn neuropathology in a mouse model of synucleinopathy by NFAT inhibition. **a** The representative images from immunohistochemical staining of total α-syn (Syn1) in the neocortex, hippocampus and striatum of the mice. Scale bar, 25 μm. **b**–**d** The levels of α-syn in the neocortex (Ctx) (**b**) hippocampus (Hipp) (**c**) and striatum (ST) (**d**) analyzed by optical density quantification (*n* = 4 or 6 per group). The data are shown as means ± s.e.m. Two-way ANOVA and Tukey’s multiple comparison test, *P < 0.05, **P < 0.01, ***P < 0.001, ****P < 0.0001. **e** The representative images from the immunohistochemical staining of PK-resistant α-syn in the neocortex, hippocampus and striatum of the mice. Scale bar, 25 μm. **f**–**h** The levels of PK-resistant α-syn in the neocortex (**f**) hippocampus (**g**) and striatum (**h**) of the mice analyzed by optical density quantification (*n* = 4 or 6 per group). The data are shown as means ± s.e.m. Two-way ANOVA and Tukey’s multiple comparison test, **P < 0.01, ****P < 0.0001. **i** The representative images from the immunohistochemical staining of phosphorylated-α-syn (81 A) in the neocortex, hippocampus and striatum of the mice. Scale bar, 25 μm. **j**–**l** The numbers of phosphor-α-syn-positive cells was counted in the neocortex (**j**) hippocampus (**k**) and striatum (**l**) of the mice (*n* = 4 or 6 per group). The data are shown as means ± s.e.m. Two-way ANOVA and Tukey’s multiple comparison test, *P < 0.05, ***P < 0.001, ****P < 0.0001. **m** The immunoblotting analysis of the mouse brains. The cytosolic and particulate lysates were probed for α-syn and β-actin. **n**, **o** The levels of α-syn in cytosolic (Cut) (**n**) and particulate (Par) (**o**) lysates were determined by densitometric quantification and normalized to β-actin (*n* = 4 per group). The data are shown as means ± s.e.m. Two-way ANOVA and Tukey’s multiple comparison test, *P < 0.05, **P < 0.01, ***P < 0.001, ****P < 0.0001. **p**, **q** The amounts of human α-syn in the cytosolic (**p**) and particulate (**q**) fractions were measured by ELISA (*n* = 5 or 6 per group). The data are shown as means ± s.e.m. Unpaired *t*-test. *P < 0.05, ****P < 0.0001.

The neuronal overexpression of human α-syn results in the abnormal accumulation of α-syn aggregates in the neuronal bodies and neuropil of α-syn-tg mice^31^. Consistent with this finding, we observed an increase of α-syn immunoreactivity in the neocortex, hippocampus and striatum of α-syn-tg mice, which was not significantly decreased by the 11R-VIVIT administration (Fig. 3a–d). The levels of PK-resistant α-syn were likewise significantly increased in the neocortex, hippocampus and striatum of α-syn-tg mice, where 11R-VIVIT administration decreased the level of PK-resistant α-syn in the neocortex (Fig. 3e–h). The numbers of phosphorylated α-syn-positive cells in the neocortex, hippocampus and striatum of α-syn-tg mice were also increased compared with non-TG mice and were significantly decreased by 11R-VIVIT administration (Fig. 3i–l). The immunoblot analysis of brain homogenates additionally demonstrated that the level of α-syn was decreased in both cytosolic and particulate fractions of α-syn-tg mice by NFAT1 inhibition (Fig. 3m–o). These findings were confirmed by a human α-syn ELISA assay (Fig. 3p, q).

NFAT1 is a critical transcriptional regulator of T cell development, activation and function^53,55,56^. Consequently, it is imperative to determine whether the peripheral T cell population might be affected by the administration of the NFAT1 inhibitor, 11R-VIVIT, in our experimental mice. To address this, we conducted a comprehensive functional T cell analysis of non-TG and α-syn-tg mice treated with either saline or 11R-VIVIT by flow cytometry (Supplementary Fig. 8a). Our analysis revealed no significant differences in the frequencies or absolute numbers of CD4^+^ and CD8^+^ T cells between the saline- and 11R-VIVIT-treated groups in both the lymph nodes and spleen (Supplementary Fig. 8b–d, o–q). Furthermore, the frequencies and absolute numbers of IL-2- and IFN-γ-producing CD4^+^ and CD8^+^ T cells remained largely unaffected by 11R-VIVIT treatment in both the lymph nodes and spleen (Supplementary Fig. 8e–n, r–aa). There was a modest increase in the frequency of IL-2-producing CD4^+^ T cells in both the lymph nodes and spleen of 11R-VIVIT-treated α-syn-tg mice compared with saline-treated mice (Supplementary Fig. 8f, s), reinforcing that the observed reduction in neuroinflammation associated with 11R-VIVIT administration is not attributable to the impact of NFAT1’s inhibition on peripheral T cells.

Taken together, these results indicate that the inhibition of NFAT1 ameliorated neuroinflammation, neurodegeneration, α-syn neuropathology and motor behavioral deficits, which could not be attributed to peripheral adaptive immunity.

### Inhibition of NFAT1 alters microglial characteristics in animal model of synucleinopathies

**Fig. 4:**
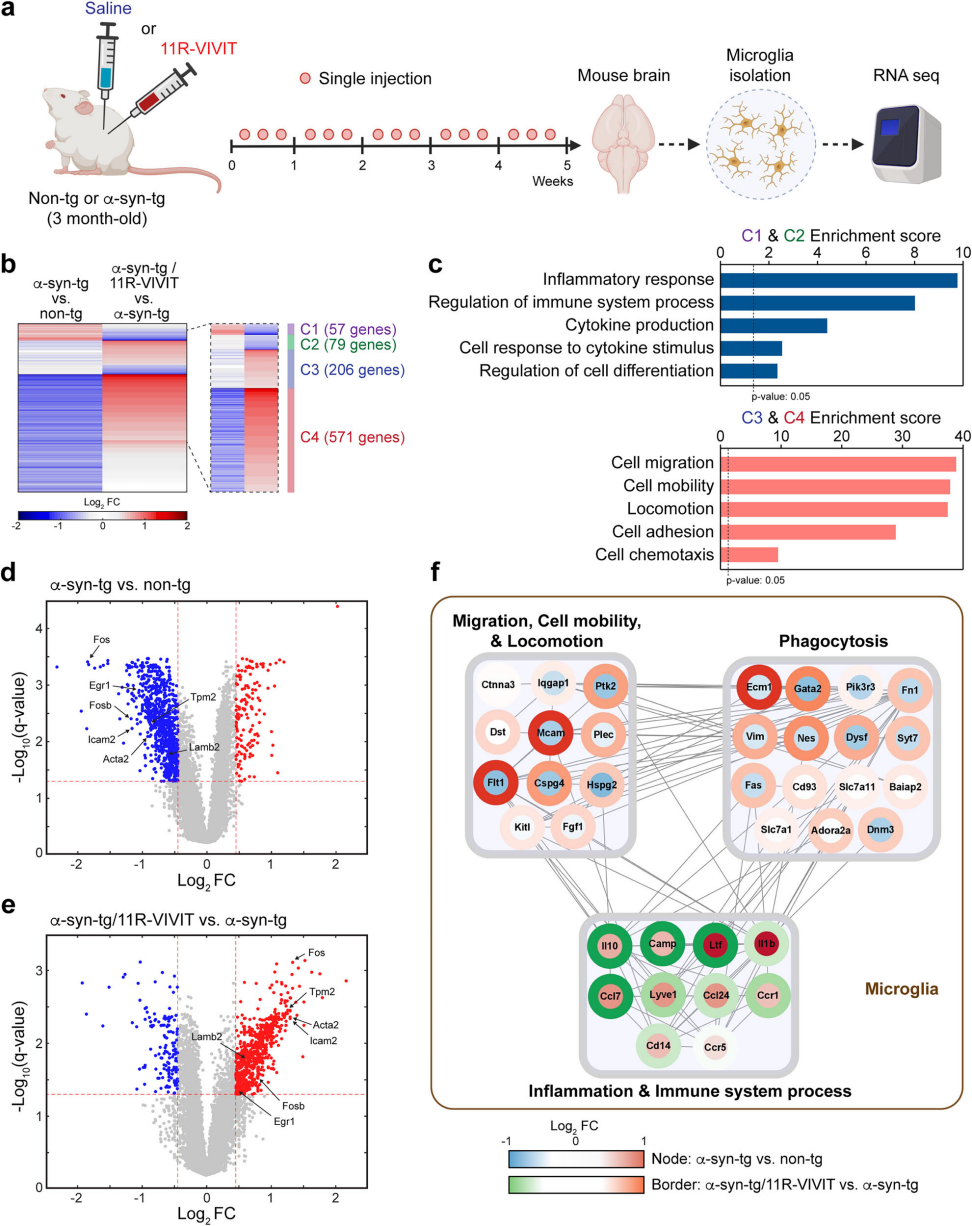
Transcriptomic analysis of microglia isolated from the mice. **a** The experimental scheme. The mouse microglia were isolated from the brains of non-TG and α-syn-tg mice injected with either saline or 11R-VIVIT; non-TG + saline, non-TG + 11R-VIVIT, α-syn-tg, α-syn-tg + 11R-VIVIT (*n* = 4 per group). The total RNA extracted from the microglia was analyzed by RNA-seq. **b** The heat map displays of the differential expression of 913 genes (Cluster 1, C1, 57 genes; Cluster 2, C2, 79 genes; Cluster 3, C3, 206 genes; Cluster 4, C4, 571 genes) in the group comparison of α-syn-tg versus non-TG and α-syn-tg-11R-VIVIT versus α-syn-tg (cutoff criteria: FDR <0.05 and the absolute value of log_2_(fold change) ≥0.45). The red and blue represent upregulation and downregulation, respectively. **c** The gene ontology terms significantly enriched (EASE score, a modified Fisher exact *P* value, <0.05) by the DEGs of clusters involved in biological processes. **d**, **e** The volcano plots of the DEGs from α-syn-tg versus non-TG (**d**) and α-syn-tg/11R-VIVIT versus α-syn-tg (**e**). The points in red indicate upregulated DEGs with FDR <0.05 and log_2_-transformed ratios of ≥0.45. The points in blue indicate downregulated DEGs with FDR <0.05 and log_2_-transformed ratios of ≤0.45. The points in gray indicate non-DEGs in the between-groups comparison. **f** The network describes PPIs between functional modules. The node and border color gradients illustrate the log_2_ fold changes (node, α-syn-tg versus non-TG; border, α-syn-tg-11R-VIVIT versus α-syn-tg). The edges (gray) represent the PPIs between genes of functional modules. FC Fold change.

Because we previously demonstrated the role of NFAT1 in α-syn-mediated neurotoxic microglial activation^29^, we hypothesized that the inhibition of NFAT1 may reduce microglial neurotoxicity and ameliorate neurodegenerative aspects in α-syn-tg mice. To verify this hypothesis, we performed microglial transcriptomic analysis utilizing microglia isolated from the age-matched non-TG and α-syn-tg mice, treated with either saline or 11R-VIVIT three times per week (Fig. 4). Young α-syn-tg mice (3-month-old), whose microglia have more sensitive reactivities against stimuli, were used to evaluate the effect of NFAT1 inhibition. After 5 weeks, microglia were isolated from the hemibrains of the mice, and transcriptome data were generated through RNA-seq (Fig. 4a). To evaluate the effects of NFAT1 inhibition, we first obtained microglial DEGs from two different comparisons; comparison (1) α-syn-tg-saline versus non-TG-saline and comparison (2) α-syn-tg-11R-VIVIT versus α-syn-tg saline-treated mice, resulting in 1,166 (145 upregulated and 1,021 downregulated) and 914 (778 upregulated and 136 downregulated) genes, respectively. To identify differences in the gene expression between non-TG and α-syn-tg mice and determine if NFAT1 inhibition altered gene expression in α-syn-tg mice, we then clustered the DEGs by differential expression pattern across groups (Fig. 4b and Supplementary Table 5). This analysis identified 913 genes that were differentially expressed between the two comparisons. The expression of genes in cluster 1 (57 genes) was increased in the microglia of α-syn-tg mice compared with the microglia of non-TG mice and decreased by 11-VIVIT treatment to the cells of α-syn-tg mice. The expression of genes in cluster 2 (79 genes) was not altered in the microglia of α-syn-tg compared with the cells of the non-TG mice but was decreased by 11R-VIVIT treatment in the cells of the α-syn-tg mice. The expression of genes in cluster 3 (206 genes) was not altered in the microglia of α-syn-tg compared with the cells of non-TG mice, but was increased by 11R-VIVIT treatment in the cells of α-syn-tg mice. Lastly, the expression of genes in cluster 4 (571 genes) was decreased in the microglia of α-syn-tg compared with the cells of non-TG, whereas they were increased by 11R-VIVIT treatment in the microglia of α-syn-tg. The functional enrichment analysis of each cluster of genes revealed that clusters 1 and 2 are enriched in microglial proinflammatory responses, and clusters 3 and 4 are enriched in microglial mobility, migration and phagocytosis (Fig. 4c and Supplementary Table 6). A volcano map of the DEGs further revealed the reduction of microglial motility associated genes, such as Icam2, Acta2, Fos, Fosb, Egr1, Lamb2 and Tpm2 in microglia of α-syn-tg compared with non-TG mice, which were increased by the 11R-VIVIT administration to the microglia of α-syn-tg mice (Fig. 4d, e). Given these enriched cellular functions and their differential expressions, we further investigated the interactions between the genes associated with the enriched cellular functions by reconstructing a network model describing the modular interactions between the microglial functions perturbed by 11R-VIVIT. The network model displays the activation of neuroinflammation and suppression of the mobility and phagocytosis of microglia of α-syn-tg compared with non-TG mice, although these were reverted by 11R-VIVIT administration (Fig. 4f).

In summary, these findings demonstrate that the administration of the NFAT1 inhibitor reversely altered microglial characteristics in α-syn-tg mice, such as the reduced expression of proinflammatory response-associated genes and increased expression of mobility and phagocytosis-associated genes.

### Inhibition of NFAT1 reduces neurotoxic inflammatory responses in microglia exposed to neuron-released α-syn

**Fig. 5:**
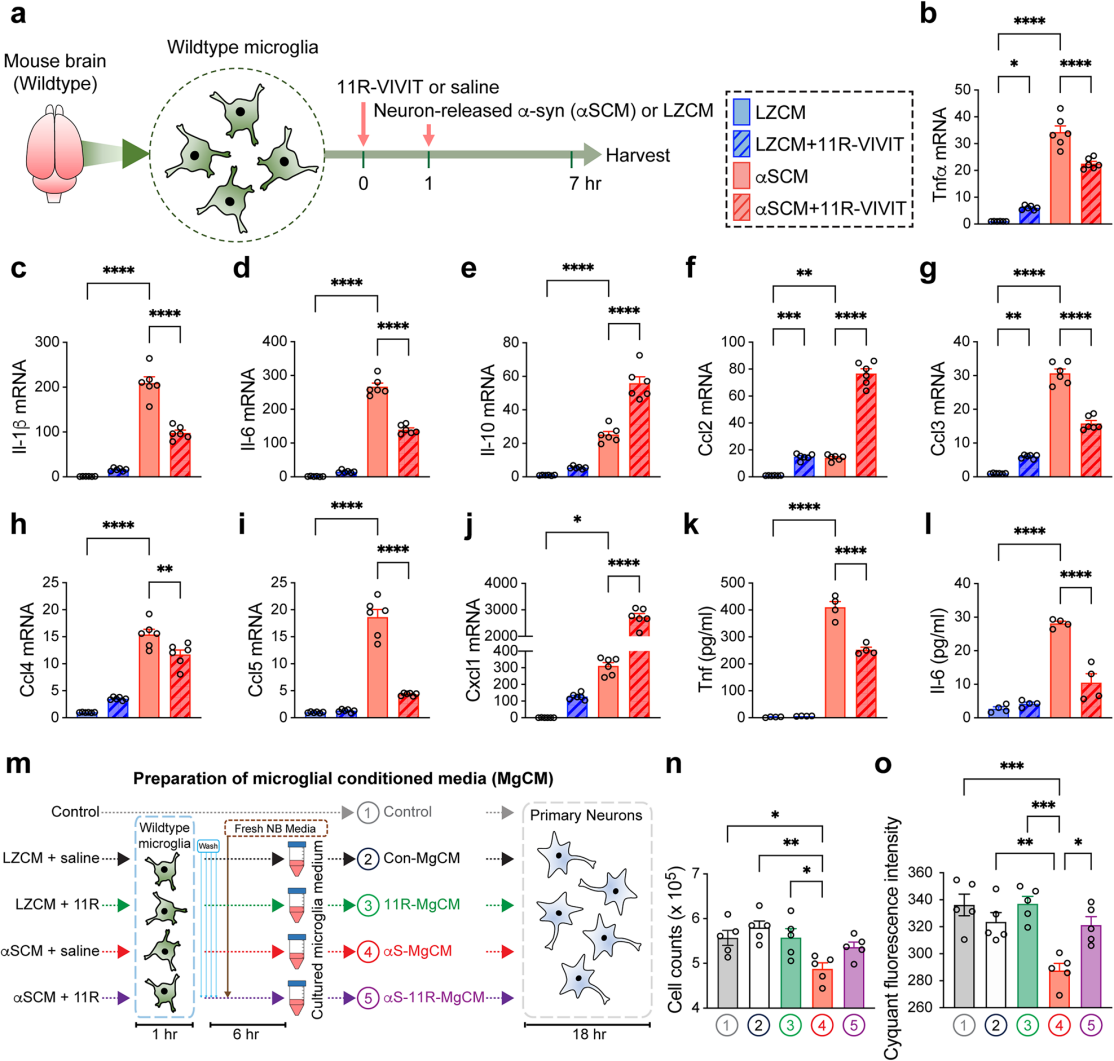
Microglial induction of neurotoxic proinflammatory responses by α-syn was reduced by NFAT1 inhibition. **a** The experimental scheme. The primary wild-type mouse microglia were pretreated with either saline or 11R-VIVIT (1 μM) for 1 h and exposed to control LZCM or neuron-released α-syn (αSCM) for 6 h. **b**–**j** The microglial expressions of Tnfα (**b**) Il-1β (**c**) Il-6 (**d**) Il-10 (**e**) Ccl2 (**f**) Ccl3 (**g**) Ccl4 (**h**) Ccl5 (**i**) and Cxcl1 (**j**) were determined by quantitative PCR (*n* = 6 per group). The data are shown as means ± s.e.m. One-way ANOVA and Tukey’s multiple comparison test, *P < 0.05, **P < 0.01, ***P < 0.001, ****P < 0.0001. **k**, **l** The microglial secretions of Tnfα (**k**) and Il-6 (**l**) were determined by ELISA (*n* = 4 per group). The data are shown as means ± s.e.m. One-way ANOVA and Tukey’s multiple comparison test, ****P < 0.0001. **m** The experimental scheme of the MgCM preparation and the determination of microglial neurotoxicity. The primary wild-type mouse neurons were treated with control media, Con-MgCM, 11R-MgCM, αS-MgCM or αS-11R-MgCM for 18 h. **n** The neuronal cell viability determined by CyQUANT cell proliferation assay. **o** The number of live neurons counted utilizing an automated cell counter (*n* = 5 per group). The data are shown as means ± s.e.m. One-way ANOVA and Tukey’s multiple comparison test, *P < 0.05, **P < 0.01, ***P < 0.001.

To verify the findings obtained from the microglial transcriptomic analysis, we first examined the anti-inflammatory effect of 11R-VIVIT on microglial activation by neuron-released α-syn (Fig. 5). The primary wild-type mouse microglia were pretreated with either saline or 11R-VIVIT for 1 h and then treated with neuron-released α-syn (αSCM, obtained from α-syn overexpressing neuronal cells) for 6 h (Fig. 5a). The exposure of microglia to neuron-released α-syn increased their expressions of proinflammatory cytokines and chemokines, including Tnfα, Il-1β, Il-6, Il-10, Ccl2, Ccl3, Ccl4, Ccl5 and Cxcl1, compared with those treated with the control conditioned media (LZCM, conditioned media obtained from β-galactosidase-overexpressing neuronal cells) (Fig. 5b–j). The pharmacological inhibition of NFAT1 in microglia significantly attenuated the αSCM-induced expression of Tnfα, Il-1β, Il-6, Ccl3, Ccl4 and Ccl5, whereas the expression of Il-10, Ccl2 and Cxcl1 were further increased by the treatment. The inductions of microglial Tnfα and Il-6 secretions by αSCM were also significantly reduced by inhibition of NFAT1, as determined by ELISA (Fig. 5k, l).

When microglia are activated by neuron-released α-syn, TLR2 and its downstream signaling cascade regulators are activated, such as phosphorylation of NFκB, p38 MAPK and NFAT1^8,29^. In accordance with previous findings, mouse primary microglia treated with αSCM increased the phosphorylation of NFκB and p38 MAPK (Supplementary Fig. 9a–c). The phosphorylation of NFAT1 at Ser54 was also increased in the nuclear fraction of mouse primary microglia exposed to αSCM (Supplementary Fig. 9d, e). However, inhibition of NFAT1 only decreased the phosphorylation of NFAT1 by neuron-released α-syn, whereas the phosphorylation of NFκB and p38 MAPK were not inhibited by 11R-VIVIT treatment (Supplementary Fig. 9a–e).

We next examined the effect of NFAT1 modulation on neuron-released α-syn-mediated microglial neurotoxicity (Fig. 5m–o). We have previously shown that the exposure to neuron-released α-syn induces microglial neurotoxicity through a series of activation steps culminating in the release of neurotoxic cytokines such as Tnfα and Il-6^29^. To determine whether NFAT1 plays a role in this process, we obtained MgCM from the cultures of mouse primary microglia exposed to either LZCM or αSCM in the presence or absence of 11R-VIVIT (Fig. 5m). Next, the primary mouse neurons were treated with control, LZCM/saline-treated MgCM (Con-MgCM), LZCM/11R-VIVIT-treated MgCM (11R-MgCM), αSCM/saline-treated MgCM (αS-MgCM) or αSCM/11R-VIVIT-treated MgCM (αS-11R-MgCM) for 18 h (Fig. 5m). The neuronal toxicity was determined in an automated and blinded manner using intact neuronal cell body counting and neuronal DNA content analysis via CyQUANT assay (Fig. 5n, o). The treatment of αS-MgCM significantly decreased both the numbers of viable cells and DNA content in primary neurons (Fig. 5n, o). By contrast, significant neuronal loss was not observed in the control, Con-MgCM-, 11R-MgCM- or αS-11R-MgCM-treated groups (Fig. 5n, o).

Taken together, these observations support that neuron-released α-syn induces microglial neurotoxicity through inductions of proinflammatory cytokines via activation of NFAT1.

### Blocking of NFAT1 activation restored cell mobility and phagocytic ability to excessively activated microglia in the synucleinopathies model

**Fig. 6:**
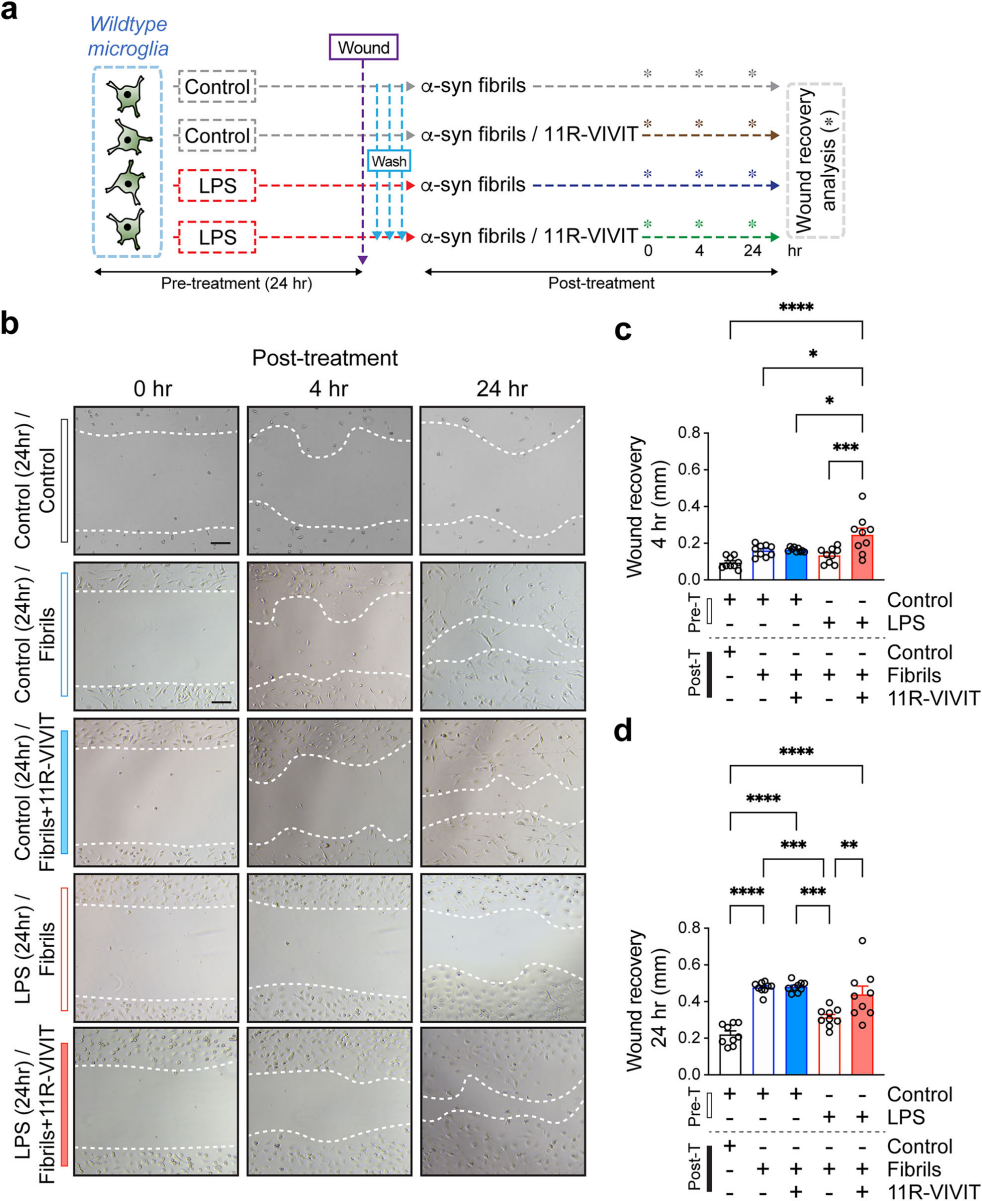
Inhibition of NFAT1 restores suppressed mobility in excessively activated microglia. Experimental scheme for microglial wound healing assay. **a** The wild-type mouse primary microglia were pretreated with control or LPS (200 ng/ml) for 24 h. After the wound generation, the cells were washed and treated with α-syn fibrils with or without 11R-VIVIT (1 μM). The recovery of the wound was measured at 0, 4 and 24 h of incubation time. **b** The representative images of microglia in the wound healing assay. Scale bar, 25 μm. **c**, **d** The width of the wound recovery was determined at 4-h (**c**) and 24-h (**d**) time points (*n* = 9 per group). The data are shown as means ± s.e.m. Two-way ANOVA and Tukey’s multiple comparison test, *P < 0.05, **P < 0.01, ***P < 0.001, ****P < 0.0001.

**Fig. 7:**
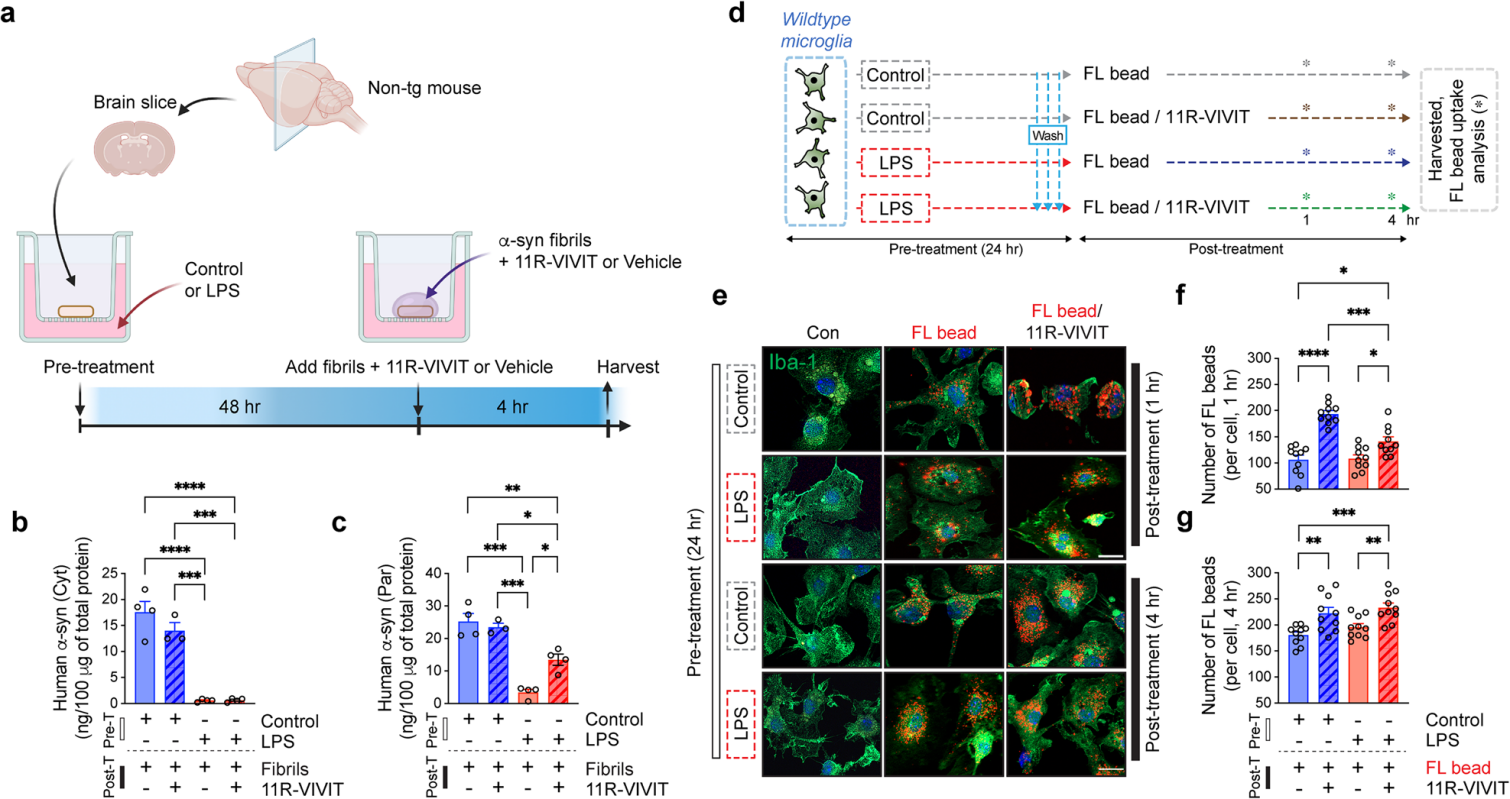
Restoration of suppressed phagocytosis by inhibition of NFAT1 in excessively activated microglia. **a** The experimental scheme for ex vivo brain slice culture and treatment; non-TG mouse brain slices were pretreated with control or LPS (1 μg/ml) for 48 h and exposed to human α-syn fibrils (500 nM) in the presence and absence of 11R-VIVIT (2 μΜ) for 4 h. The brain slices were then collected and separated into cytosolic and particulate fractions. **b**, **c** The levels of internalized human α-syn in cytosolic (**b**) and particulate (**c**) fractions of the brain slices determined by human α-syn ELISA (*n* = 4 per group). The data are shown as means ± s.e.m. Two-way ANOVA and Tukey’s multiple comparison test, **P < 0.01, ****P < 0.0001. **d** The experimental scheme of the microglial phagocytosis assay. The primary wild-type mouse microglia were pretreated with either control or LPS (1 μg/ml) for 24 h. Then, the control and activated microglia were treated with fluorescent beads in the presence or absence of 11R-VIVIT (1 μM) for 1 and 4 h. **e** The representative images from the microglial fluorescent bead-based phagocytosis assay. Scale bar, 20 μm. **f**, **g** The quantitative analysis of microglial fluorescent beads internalized at 1 (**f**) and 4 (**g**) h (*n* = 10 per group). The data are shown as means ± s.e.m. Two-way ANOVA and Tukey’s multiple comparison test, *P < 0.05, **P < 0.01, ***P < 0.001, ****P < 0.0001.

Given that our transcriptome analysis revealed that the administration of 11R-VIVIT accelerates the mobility of excessively activated microglia in α-syn-tg mice, we next evaluated its effect on microglial migration and phagocytosis (Figs. 6 and 7 and Supplementary Fig. 10). We first determined the migration ability of the microglia via a wound healing assay. Although αSCM and LPS activate microglia through different pathways, such as TLR2 and TLR4, we demonstrated that the cells thereby excessively activated exhibit similar characteristics, including expression patterns of microglial mobility-associated genes and morphology (Supplementary Fig. 10a–c). As a result, we first sought to induce excessive activation in microglia with LPS to distinguish microglia stimulation from further extracellular α-syn treatment. The primary mouse microglia were treated with either control or LPS for 24 h to obtain control and LPS-induced excessively activated microglia. Next, the microglial cell cultures were scratched and treated with pathogenic α-syn aggregates (α-syn fibrils) to mimic the disease condition in the presence or absence of 11R-VIVIT (Fig. 6a). The width of the wound was determined after 0, 4 and 24 h of incubation (Fig. 6a). After 4 h, the recovery rate of the wound was not altered in the control or excessively activated microglia, whereas 11R-VIVIT treatment increased the wound recovery rate of only excessively activated microglia (Fig. 6b, c). By contrast, 24 h after α-syn fibrils exposure, the recovery rate of the wound was delayed in the excessively activated microglia compared with control cells but was rescued by 11R-VIVIT treatment (Fig. 6b, d).

We next examined whether inhibition of NFAT1 affects microglial phagocytosis of extracellular α-syn aggregates (Fig. 7a–c). To address this, we used an ex vivo mouse brain slice culture. We first pretreated non-TG mouse brain slices with either control or LPS for 48 h. After the pretreatment, the brain slices were exposed to human α-syn fibrils in the presence or absence of 11R-VIVIT (Fig. 7a). After a 4-h incubation, the brain slices were collected, and cytosolic and particulate α-syn were analyzed by human α-syn ELISA (Fig. 7b, c). The incubation with α-syn fibrils was done for only a short period to distinguish the microglial α-syn uptake from other brain cell uptake, as microglia are the fastest in internalizing extracellular α-syn among brain cells such as neurons and astrocytes^1,57^. Interestingly, the level of internalized human α-syn was significantly reduced in the LPS-treated group compared with the control group in both cytosolic and particulate fractions, although the 11R-VIVIT treatment partly recovered α-syn internalization only in the particulate fraction (Fig. 7b, c). However, the treatment of 11R-VIVIT did not induce a further increase of human α-syn internalization in the control group (Fig. 7b, c). The microglial phagocytosis ability was further confirmed with a fluorescence-bead-based phagocytosis assay (Fig. 7d–g). After 24 h of treatment with either control or LPS, mouse primary microglia were incubated with fluorescent beads in the presence or absence of 11R-VIVIT for 1 and 4 h (Fig. 7d). In contrast to the α-syn fibril uptake analysis, the internalization of fluorescent beads was not delayed in excessively activated microglia (Fig. 7e–g). However, the treatment with 11R-VIVIT increased the microglial uptake of fluorescent beads in both the control and LPS-induced excessively activated microglia (Fig. 7e–g).

We next investigated the microglial state by utilizing gene expression profiling analysis (Supplementary Fig. 11). The primary mouse microglia were pretreated with either LZCM or αSCM for 24 h to obtain control and α-syn-induced excessively activated microglia. The cells were then incubated with or without 11R-VIVIT for 4 h, after which, the gene expression was determined by quantitative PCR (Supplementary Fig. 11a). The expressions of proinflammatory cytokines and chemokines, including Tnfα, Ccl2, Ccl3, Ccl4, Cxcl1 and Cxcl2, were increased at 24 h of αSCM treatment^8^, and all were found to be recovered by the 4-h time point except for Il-6 and Ccl5 (Supplementary Fig. 11b–i). Interestingly, post-treatment with 11R-VIVIT increased the expression of all proinflammatory cytokine and chemokine genes in both control and activated microglia except for Ccl5 (Supplementary Fig. 11b–i). Among them, the expressions of Il-6 and Cxcl2 were significantly increased by 11R-VIVIT treatment in only activated microglia and in both the control and activated microglia, respectively (Supplementary Fig. 11c, i). Similar to the microglia transcriptome analysis, the expressions of microglia mobility- and phagocytosis-associated genes, such as Fos, Fosb and Egr1, were significantly increased by 11R-VIVIT post treatment in both control and activated microglia, whereas Acta2 and Icam2 expressions were significantly increased by 11R-VIVIT only in activated microglia (Supplementary Fig. 11j–n). Post-treatment with 11R-VIVIT also increased the expression of Il-10, an anti-inflammatory cytokine, in activated microglia (Supplementary Fig. 11o). The expressions of microglial phenotype-associated genes exhibited a complex pattern in both the control and excessively activated microglia (Supplementary Fig. 11p–s). Both Cd68 and Cd86 were decreased in excessively activated microglia, but only the expression of Cd86 was increased by 11R-VIVIT treatment in both the control and excessively activated microglia (Supplementary Fig. 11p, q). By contrast, the expression of Arg1 was significantly increased in excessively activated microglia and decreased by 11R-VIVIT post-treatment (Supplementary Fig. 11r), whereas Cd206 was decreased in the microglia and not rescued by 11R-VIVIT post-treatment (Supplementary Fig. 11s). The expressions of neurotrophic growth factors Bdnf and Tgfb1 were not altered by microglial activation state, whereas Gdnf expression was slightly decreased in excessively activated microglia (Supplementary Fig. 11t–v). Interestingly, the level of Bdnf was increased by 11R-VIVIT post-treatment in both control and excessively activated microglia (Supplementary Fig. 11t). The expressions of microglial senescence-associated genes, such as Cdkn2a/p16 and Glb1/SAbgal, were not affected by the microglial activation state or post-treatment with 11R-VIVIT (Supplementary Fig. 11w, x).

Taken together, these findings support that the inhibition of NFAT1 restored the mobility and phagocytic abilities of excessively activated microglia via gene expression alteration, although further evaluation is needed to determine the microglial state.

### NFAT1 and microglial-mobility- and phagocytosis-associated genes in PD/DLB

**Fig. 8:**
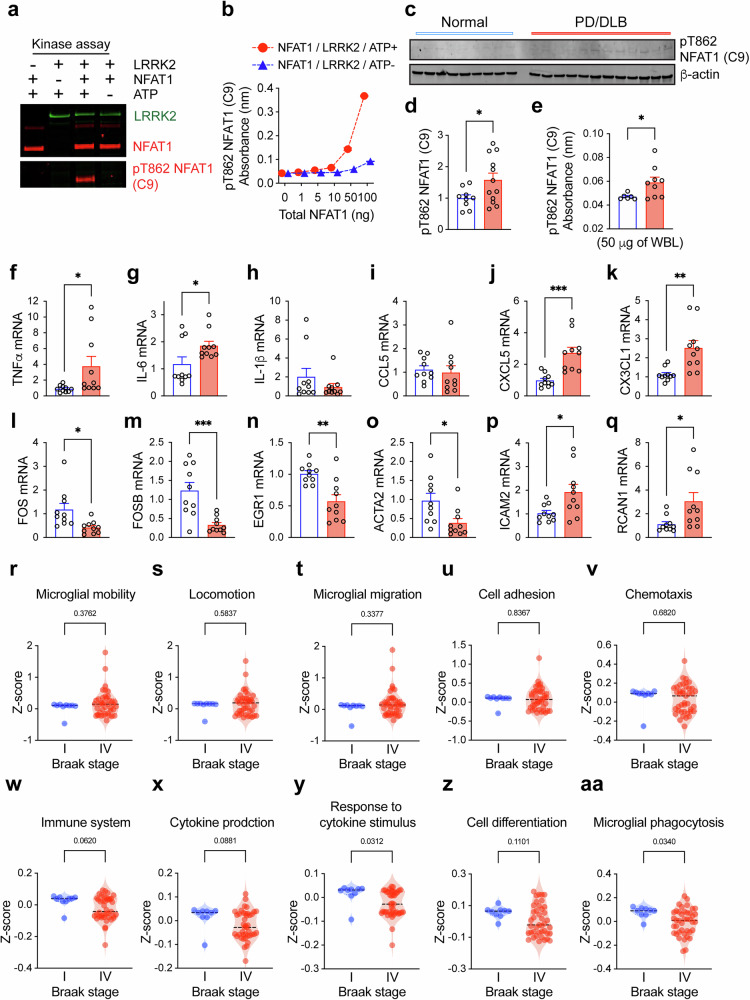
Activation of NFAT1 and suppression of microglial mobility and phagocytosis ability in patients with PD/DLB. **a** The in vitro LRRK2 kinase assay. Recombinant LRRK2 wild type was incubated with NFAT1 in the presence or absence of ATP for 30 min. The samples were analyzed by immunoblot analysis. The blot was first incubated with anti-pT862 NFAT (C9) and probed with anti-LRRK2 and anti-NFAT1. **b** The level of phosphorylated NFAT1 (T862) in the sample were determined by a dose-gradient sandwich ELISA. **c** The immunoblot analysis of particulate fractions of normal patients and patients with PD/DLB. **d** The level of phosphorylated NFAT1 (T862) was determined by densitometric quantification and normalized to β-actin (*n* = 9 or 12 per group). The data are shown as means ± s.e.m. Unpaired *t*-test, *P < 0.05. **e** The level of phosphorylated NFAT1 (T862) in the human whole brain lysates was analyzed by ELISA (*n* = 6 or 9). The data are shown as means ± s.e.m. Unpaired *t*-test, *P < 0.05. The quantitative gene expression analysis of human brains. **f**–**q** The expressions of TNFα (**f**) IL-6 (**g**) IL-1β (**h**) CCL5 (**i**) CXCL5 (**j**) CX3CL1 (**k**) FOS (**l**) FOSB (**m**) EGR1 (**n**) ACTA2 (**o**) ICAM2 (**p**) and RCAN1 (**q**) were determined by quantitative PCR (*n* = 10 per group). The data are shown as means ± s.e.m. Unpaired *t*-test, *P < 0.05, **P < 0.01, ****P < 0.0001. **r**–**aa** The violin plots indicating distributions of transcriptional signature activities in the brains of patients with PD; each point indicates a *Z*-score from a sample of lower (I) and higher Braak stages (IV) from the brains of patients with PD; the lines indicate means of the *Z*-scores; the signature activities are microglial mobility (**r**) locomotion (**s**) microglial migration (**t**) cell adhesion (**u**) chemotaxis (**v**) immune system (**w**) cytokine production (**x**) response to cytokine stimulus (**y**) cell differentiation (**z**) and microglial phagocytosis (**aa**) (*n* = 9 or 41 per group). The data are shown as means ± s.e.m., unpaired *t*-test. *P* values are indicated in the graph.

The microglial transcriptome analysis showed that the induction of proinflammatory cytokines and chemokines and the downregulation of microglial-mobility- and phagocytosis-associated genes in excessively activated microglia of the synucleinopathy mouse model while these were rescued by NFAT1 inhibition (Fig. 4). To verify these observations in a human model, we investigated the identified genes from the microglial transcriptome of human synucleinopathies such as PD/DLB. We first evaluated the activation status of NFAT1 in the brains of synucleinopathies. We previously identified five residues, T483, T733, T862, T870 and T893, of NFAT1, which could be phosphorylated by the activation of TLR2 and the following LRRK2 signaling cascade in microglia^29^. Among them, we successfully generated an anti-mouse antibody (C9) that recognizes phosphorylated NFAT1 at the T862 residue in cooperation with GenScript. The detection of phosphorylated NFAT1 in the samples obtained from the LRRK2 kinase assay verified the specificity of the C9 antibody (Fig. 8a, b). The immunoblot analysis demonstrated that phosphorylated NFAT1 at T862 was detected only in the sample incubated with wild-type LRRK2 in the presence of ATP, whereas it was not detected in the sample without ATP or with NFAT1 alone (Fig. 8a). The ELISA assay also revealed that the absorbance of the C9 antibody was only increased in response to the increment of the total amount of NFAT1 (Fig. 8b). Next, we determined the level of pT862 NFAT1 in the brains of control and patients with PD/DLB (Fig. 8c–e). The fresh brain homogenates were separated into cytosolic and particulate fractions and then analyzed by immunoblot analysis (Fig. 8c, d and Supplementary Fig. 12). The amount of monomeric α-syn was unaltered in both cytosolic and particulate fractions of brain homogenates (Supplementary Fig. 12a–c). However, the level of oligomeric α-syn was significantly increased in cytosolic and particulate fractions of patients with PD/DLB (Supplementary Fig. 12a, d, e). The phosphorylation of NFAT1 (T862) was not detected in the cytosolic fraction of both normal patients and patients with PD/DLB. However, the level of pT862 NFAT1 was increased in the particulate fraction of patients with PD/DLB compared with controls (Supplementary Fig. 8c, d). This was confirmed by a whole brain lysate with NFAT1 and anti-pT862 NFAT1 sandwich ELISA assay (Fig. 8e).

To investigate the microglial feature in the brains of patients with PD/DLB, we next analyzed the microglial gene expressions (Fig. 8f–q and Supplementary Fig. 12f–l). The expressions of proinflammatory cytokines and chemokines TNFα, IL-6, CXCL5 and CX3CL1 were increased in the brains of patients with PD/DLB compared with normal subjects (Fig. 8f, g, j, k), although levels of IL-1β and CCL5 were not (Fig. 8h, i). Interestingly, the expressions of microglial mobility- and phagocytosis-associated genes such as FOS, FOSB, EGR1 and ACTA2 were decreased in the brains of patients with PD/DLB compared with those of normal subjects (Fig. 8l–o). The expression of ICAM2 was increased in patients with PD/DLB compared with normal subjects (Fig. 8p). This is different from the in vitro observation, where Icam2 was not increased in control and excessively activated microglia but induced by 11R-VIVIT treatment only in activated microglia (Supplementary Fig. 11n). The levels of microglial phenotype-associated genes such as ARG1 were increased in both cytosolic and particulate fractions of patients with PD/DLB compared with normal subjects (Supplementary Fig. 12f–h). However, the expressions of IL-10 and CD206 were decreased in both cytosolic and particulate fractions of patients with PD/DLB (Supplementary Fig. 12f, i–l). The expression of an NFAT1 modulator RCAN1 (calcineurin inhibitor) was also upregulated in the brains of patients with PD/DLB (Fig. 8q).

To assess whether NFAT-governed transcriptional programs (Fig. 4c) are associated with the progression and development of neurodegenerative diseases, we calculated gene signature activity with the transcriptome data of normal subjects and patients with PD from the GEO database (GSE182622) using *Z*-score method^47^ (Fig. 8r–aa, Supplementary Fig. 13 and Supplementary Table 6). To investigate the association of the gene expression alteration with disease progression, we analyzed lower (I) and higher (VI) Braak stage groups of the patients. The microglial mobility, locomotion, migration, cell adhesion and chemotaxis were significantly increased in patients with PD compared with normal subjects (Supplementary Fig. 13a–e). These were not altered in the higher Braak stage as compared with the lower Braak stage group (Fig. 8r–v). The immune system, cytokine production and microglial phagocytosis showed increasing trends in patients with PD compared with normal subjects, whereas immune system and cytokine production were significantly decreased in the higher Braak stage group compared with the lower Braak stage group (Fig. 8w, x and Supplementary Fig. 13f, g). The response to cytokine stimulus and cell differentiation were decreased in the higher Braak stage group compared with the lower Braak stage group (Fig. 8y, z). Finally, microglial phagocytosis was not altered in patients with patients with PD compared with normal subjects, whereas it was significantly decreased in the higher Braak stage group compared with the lower Braak stage group (Fig. 8aa and Supplementary Fig. 13h).

Taken together, these findings support the alteration of the microglial state and the downregulations of the microglial migration and phagocytosis-associated genes in the brains of disease-affected patients.

## Discussion

The microglia play central roles in neuroinflammation and neurohomeostasis and have various cell states, which should be determined through a multitude of analyses including transcriptomics, proteomics and metabolomics^58^. Furthermore, exposure to stimuli induces microglia activation and increases their expressions of proinflammatory cytokines and chemokines^29,31^. In the current study, we demonstrated that the expressions of these neurotoxic proinflammatory cytokines and chemokines were increased in chronically activated microglia, whereas their cellular mobility and phagocytosis activity were suppressed. We termed these as ‘excessively activated microglia’, which we then isolated from the mouse model. We also found that the translocation of the activated NFAT1 to the nucleus was increased in the microglia of the synucleinopathy mouse model. The pharmacological inhibition of NFAT1 not only decreased neurotoxic proinflammatory responses but also restored the mobility and phagocytic ability of the microglia, thereby redirecting excessively activated microglia toward an active healthy microglia state (Supplementary Fig. 14). These findings support the hypothesis that the functional inhibition of NFAT1 might be of therapeutic interest for synucleinopathies.

NFAT is a family of transcription factors and a well-known modulator of T cell activation and differentiation^59–62^. The NFAT family consists of five proteins, NFAT1 (NFATc2 or NFATp), NFAT2 (NFATc1 or NFATc), NFAT3 (NFATc4), NFAT4 (NFATc3 or NFATx) and NFAT5 (TonEBP or OREBP)^62^. Although NFAT1 has been known to modulate the process of immune cell activation, recent studies have also suggested the role of NFAT1 in cellular migration processes such as tumor cell invasion^63^. Although the underlying mechanism is not yet understood, studies have shown that activation of NFAT1 increases the invasion and migration of breast tumor cells via the upregulation of cyclooxygenase 2 (Cox-2). In the current study, the alteration of Cox-2 expression was not detected in the microglia of α-syn-tg mice (Supplementary Table 5). Instead, we demonstrated the suppression of other microglial mobility/phagocytosis-associated genes such as Icam2, Acta2, Fos, Fosb, Egr1, Lamb2 and Tpm2 in both synucleinopathy patients and the animal model (Figs. 4 and 8). Strikingly, the pharmacological inhibition of NFAT1 rescued these suppressions in the microglia of α-syn-tg mice (Fig. 4). These findings strongly support that NFAT1 was induced in the excessively activated microglia of synucleinopathies, which suppresses the expression of genes associated with microglial mobility and phagocytosis. Excitingly, the inhibition of NFAT1 can ameliorate the deficits of synucleinopathy by redirecting the excessively activated microglia to active healthy microglia through the suppression of neuroinflammation and the restoration of cellular mobility and phagocytosis abilities.

NFAT is known as a transcription factor regulated by the phosphorylation and dephosphorylation of multiple residues^64,65^. The canonical NFAT activation process is by the Ca^2+^–calcineurin signaling cascade^52,65^. However, recent studies also demonstrated NFAT1’s interaction with LRRK2, though these findings are conflicting as to whether LRRK2 activates or inhibits NFAT1. Liu et. al reported that LRRK2 negatively modulated nuclear localization of NFAT1 in bone-marrow-derived macrophages^66^. The study showed the increase of NFAT1 immunoreactivity in the nucleus of colon sections of *Lrrk2* deficient mice compared with wild-type mice, and nucleus localization of NFAT1 was accelerated in *Lrrk2* deficient bone-marrow-derived macrophages. However, we recently demonstrated that the kinase activity of LRRK2 induced activation and nucleus translocation of NFAT1 in the microglia of the synucleinopathy mouse model^29^. Furthermore, Yan et. al demonstrated that the treatment of neurotoxin acrylamide activated LRRK2 and increased the nuclear translocation of NFAT1 in mouse microglia cell line^67^. In addition, the kinase enhanced LRRK2 microglia (G2019S) led to neurite shortening, which is indicative of neurotoxicity^68^. According to these findings, NFAT1 and LRRK2 seem to interact but may have different characteristics depending on cell type, and further research in this area is necessary.

We used a progressive mouse model of synucleinopathies using mice overexpressing human wild-type α-syn driven by the murine Thy-1 promoter. The mouse model reproduces many features of synucleinopathies including progressive changes in dopamine release, α-syn neuropathology, neuroinflammation, neurodegeneration and behavioral deficits such as motor and nonmotor dysfunctionality^30,69^. Therefore, this mouse model has been widely used for preclinical studies on synucleinopathies^30,69^. In the current study, we observed locomotor and motor coordination deficits as well as anxiety-like hyperactivity in the α-syn-tg mice. Although locomotor deficits of α-syn-tg mice were rescued by the administration of NFAT1 inhibitor on the first day of rotarod test, we could not observe statistically significant differences on days 2 and 3 (Fig. 2i, j). The average distance traveled by the mice on the rotarod increased over the test days, which has been known to be indicative of motor skill learning^70^ (Fig. 2k). Therefore, we recognize that the motor skill learning ability of α-syn-tg mice was not affected by α-syn overexpression despite exhibiting locomotor deficits.

In our study, we observed that αSCM reduced the messenger RNA (mRNA) expression of Cd68 after 24 h of treatment, which was not recovered in the control or 11R-VIVIT post-treatment in a short period (Supplementary Fig. 11p). CD68 is known to be associated with microglial phagocytosis, and the induction of CD68 was observed in the substantia nigra of patients with PD and animal models^25,71,72^. However, other studies also showed impaired phagocytic function of cells including microglia in patients with PD and animal models despite an increase in the expression of CD68^73,74^. In addition, studies demonstrated that CD68 induction is associated with dopamine neuronal death^75^. Following the association of CD68 with immune cell phagocytosis, the upregulation of CD68 was also reported in cell cultures when cells were exposed to proinflammatory cytokines^76^. Similarly, recent studies also demonstrated the reduction of the expression of CD68 in primary rat microglia after 24 h of LPS alone and LPS with other cytokine treatments^77^. Therefore, we speculate that the improvement of microglial phagocytosis with NFAT1 inhibition in the current study might be associated with a CD68-independent pathway.

α-syn is a typical neuronal cytosolic protein; however, small amounts of α-syn aggregates can be secreted into the extracellular space and transferred to neighboring cells, such as microglia, astrocytes and neurons, thereby mediating the neurotoxic inflammation of glial cells^1,12^. Although the mechanism of cell-to-cell transmission of α-syn has not been fully elucidated, several receptors have been known to interact with extracellular α-syn, such as TLR2, TLR4, LAG3, CD36 and FcγR^8,78–80^. Among them, we have demonstrated the pathogenic role of microglial TLR2 in synucleinopathies; therefore, we proposed TLR2 as a new therapeutic target for synucleinopathies^12^. However, it is also worth identifying more specific downstream targets for synucleinopathies considering TLR2 is the most upstream signaling receptor in these cells. TLR2 is primarily an innate immune receptor; thereby, it mainly modulates the immune response, but it has also been shown to modulate various cellular responses, such as the production of glial-derived neurotrophic factor and the architecture of the enteric nervous system^81,82^. Hence, the benefits of TLR2 inhibition in synucleinopathies might be limited owing to its potential off-target effects. To overcome these limitations, we investigated the downstream signaling cascade of TLR2 in synucleinopathies and revealed the activation of a signaling cascade including LRRK2 and NFAT1 in microglia in the context of synucleinopathies^29^. This is an NFκB-independent pathway—therefore, the inhibition of the downstream elements of this cascade may reduce the off-target effects of TLR2 inhibition in synucleinopathies and selectively reduce microglial neurotoxic inflammation while avoiding the inhibition of NFκB-dependent pathways that may play central roles in microglial neurohomeostasis^83,84^. Therefore, we proposed NFAT1 as a new drug target for synucleinopathies and used 11R-VIVIT, an NFAT1 inhibitory peptide, as a tool compound to more comprehensively understand the effects of NFAT1 inhibition on microglial phenotype and interrogate the pathways involved to shed further light on its potential as a therapeutic target.

Due to the importance of NFAT in the peripheral immune system, it has been extensively studied in various peripheral immune-related diseases and immune-modulating therapies, such as various cancers and organ transplantation^85^. Due to the lack of NFAT-specific inhibitors, cyclosporine and/or tacrolimus, which modulate calcineurin, a master regulator of NFAT1, were first utilized in various allergic and autoimmune diseases to modulate the function of NFAT^85^. However, the treatment of cyclosporine and/or tacrolimus can also suppress the host immune system extensively, causing a wide variety of serious side effects, such as cardiovascular disorders, hypertension, malignancies and organ disorders, including those of the kidney, liver and pancreas^86–88^. Therefore, to reduce the concerns for side effects, new agents such as 11R-VIVIT, A-285222 and INCA-6, which are able to directly modulate NFAT activity, have been developed^89–91^. Among them, the 11R-VIVIT peptide has been developed and extensively tested in various animal models^92^. The administration of 11R-VIVIT efficiently suppressed the activity of NFAT in mouse models of various diseases, such as heart disease, colitis, bronchial asthma and type 2 diabetes with lesser side effects^93–96^. Unlike disease models in which the peripheral inflammatory responses are activated, the animal model used in the current study exhibits the activation of brain inflammatory responses rather than the activation of peripheral inflammatory responses^69^. In addition, deficiency of NFAT1 has been known to induce altered immune responses such as allergic contact hypersensitivity and T cell exhaustion^97^. Although we demonstrated that the development and functions of T cells were not affected by the short-term inhibition of NFAT1 in this mouse model, the long-term inhibition of NFAT1 may cause different immune reactions in the mice. Therefore, more in-depth studies should be conducted to evaluate the long-term treatment effect of 11R-VIVIT in the immune system of the host.

In the current study, we validated the brain trafficking and engagement of peripherally administrated 11R-VIVIT in a synucleinopathy mouse model. Furthermore, we verified that the treatment of 11R-VIVIT can ameliorate the neuropathology and behavioral deficits of the synucleinopathy mouse model. We also provided the effect and related mechanism of 11R-VIVIT treatment, which redirect excessively activated microglia to active healthy microglia in the synucleinopathy animal model. However, although the advantages of 11R-VIVIT treatment have been validated in vivo, its clinical application has not yet been addressed. Consistent with previous studies^30,69^, we also observed reduced microglial activation in the neocortex and hippocampus of 11R-VIVIT-administrated α-syn-tg mice, but striatal glial activations were not rescued by the inhibition. Although recent studies demonstrated that intraperitoneally injected peptide could reach various brain regions including the neocortex, hippocampus, hypothalamus and amygdala, it is not yet confirmed whether the injected peptide can reach to the striatum^98–101^. In addition, the amount of peptide delivered to each brain region might be affected by several factors, including regional differences in the BBB and characteristics of the peptide^100,102–104^. Furthermore, we previously demonstrated that NFAT1 is expressed in neurons and astrocytes, but its particular role in those cells are not explored yet^29^. Therefore, it is possible that the systemic inhibition of NFAT1 may modulate the function of NFAT1 in neurons and astrocytes as well, thereby reducing neuroinflammation and neurodegeneration. For these reasons, further research to address these ambiguities are required before utilizing 11R-VIVIT as a therapeutic approach.

## Supplementary information

Supplementary information

## Data Availability

All data needed to evaluate the conclusions in the Article are present in the Article and/or the Supplementary Information. The RNA-seq data have been deposited in the Gene Expression Omnibus (GEO) of NCBI under the accession number GSE223625.
