# Supplementary material for: Redirecting microglia phenotype via inhibition of NFAT1 ameliorates deficits in mouse model of synucleinopathies
Source: Exp Mol Med. 2025 Nov 17;57(11):2588–607. doi: 10.1038/s12276-025-01564-4 (PMC12686539; doi:10.1038/s12276-025-01564-4)
Supplement: Supplementary file 1 — Supplementary information [file 12276_2025_1564_MOESM1_ESM.pdf]

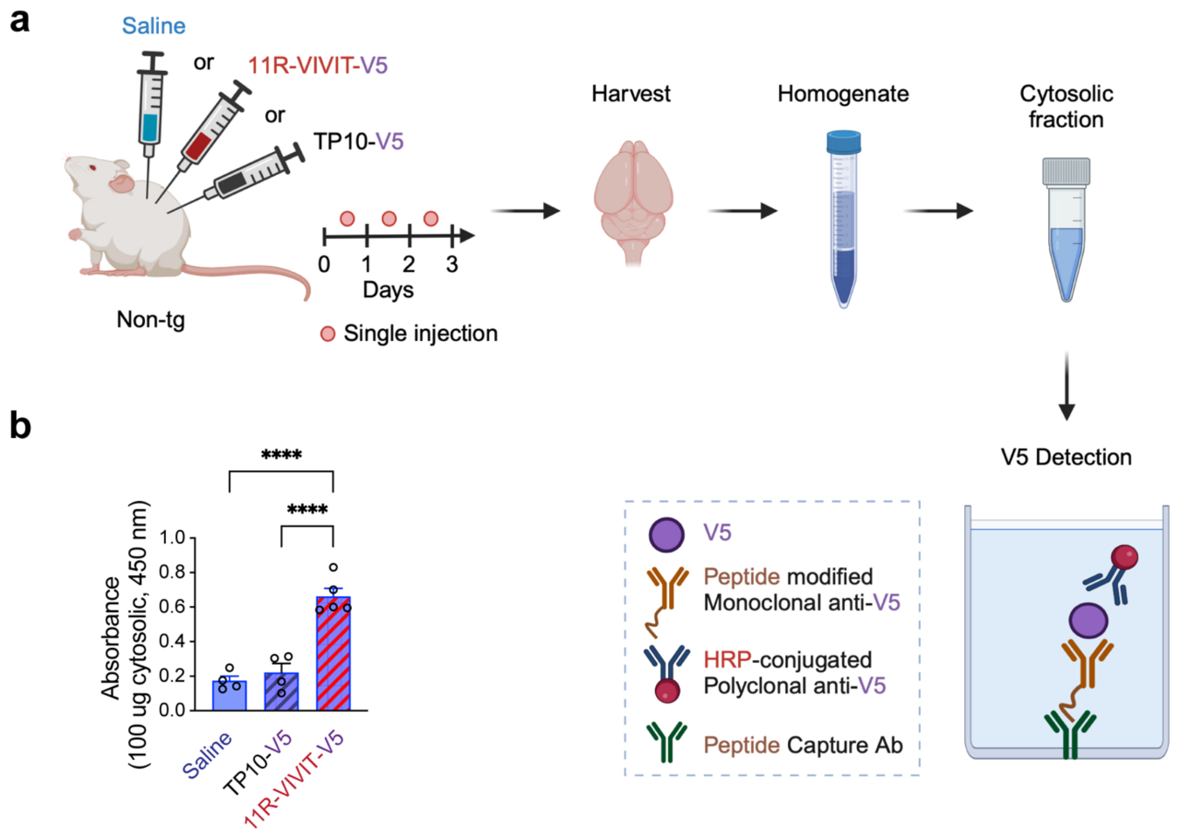

Supplementary Fig. 1 Validation of brain infiltration of peripherally administrated peptides. a Experimental scheme. Eight-month-old Non-tg mice were injected with either saline, 11R-VIVIT-V5, or TP10-V5 daily for 3 days. One hour after the final injection, the mice were harvested, and their brains were homogenated. V5-tagged peptides were detected in the cytosolic fraction of brain homogenates. **b** The level of V5 in the cytosolic fraction was determined by custom V5 ELISA assay (n = 4 or 5 per group). Data are shown as means ± SEM. One-way ANOVA and Tukey's multiple comparison test. ***: $p < 0.001$.

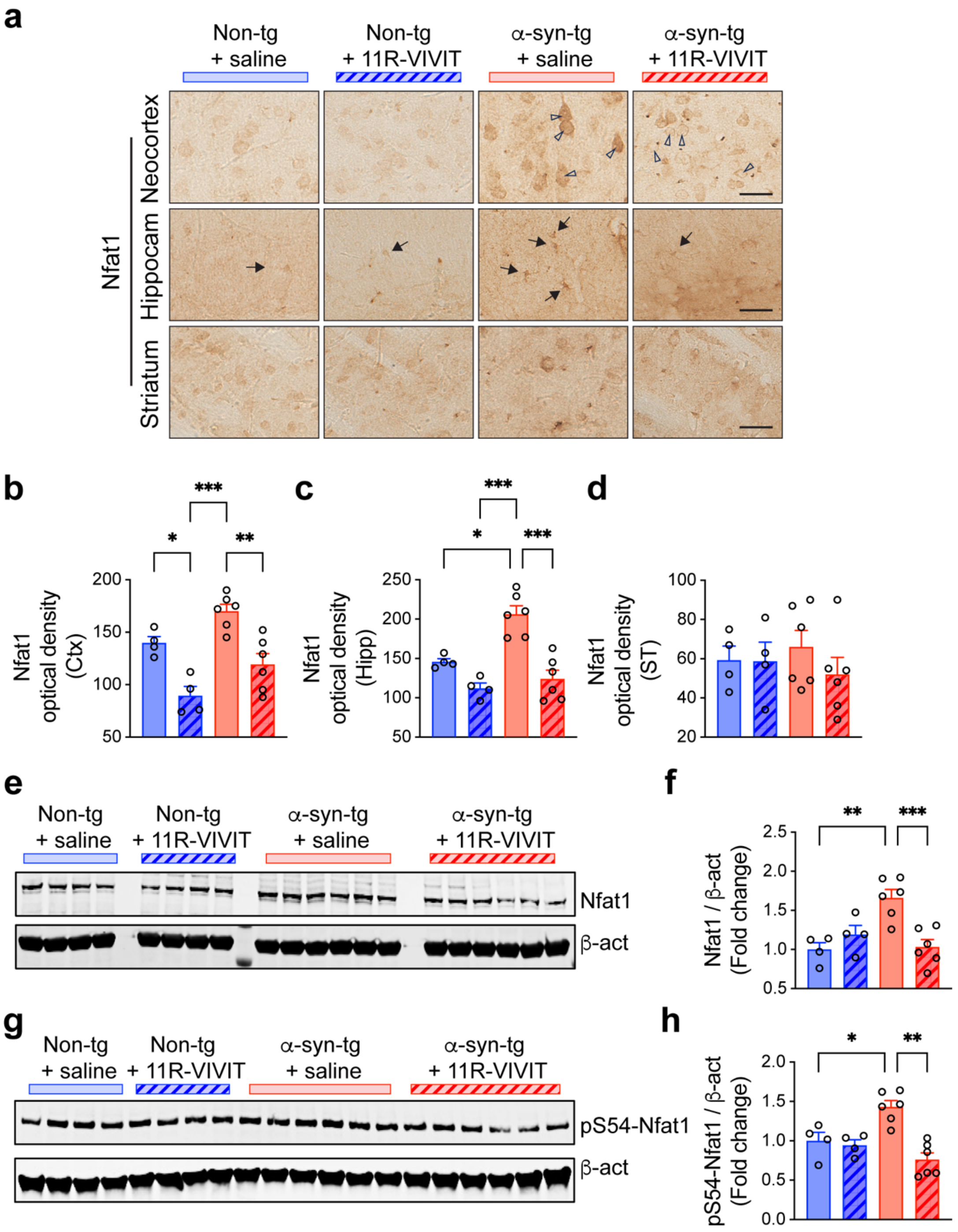

Supplementary Fig. 2 Administration of NFAT1 inhibitor reduced accumulation of NFAT1 in a mouse model of synucleinopathy. a Representative images from immunohistochemical staining of Nfat1 in the neocortex, hippocampus, and striatum of the mice. Arrowheads indicate nuclei of Nfat1-positive cells and arrows indicate Nfat1-positive cells. Scale bar, 25 mm. **b-d** The levels of Nfat1 in the neocortex (**b**), hippocampus (**c**), and striatum (**d**) were analyzed by optical density quantification (*n* = 4 or 6 per group). Data are

means ± SEM. **e**-**h** Immunoblot analysis of Nfat1 (**e**) and phosphor-S54-Nfat1 (**g**). The levels of Nfat1 (**f**) and phosphor-S54 Nfat1 (**h**) were determined by densitometric quantification and normalized to β-actin (n = 4 or 6 per group). Data are shown as means ± SEM. *: $p < 0.05$; **: $p < 0.01$; ***: $p < 0.001$. Two-way ANOVA and Tukey's multiple comparison test.

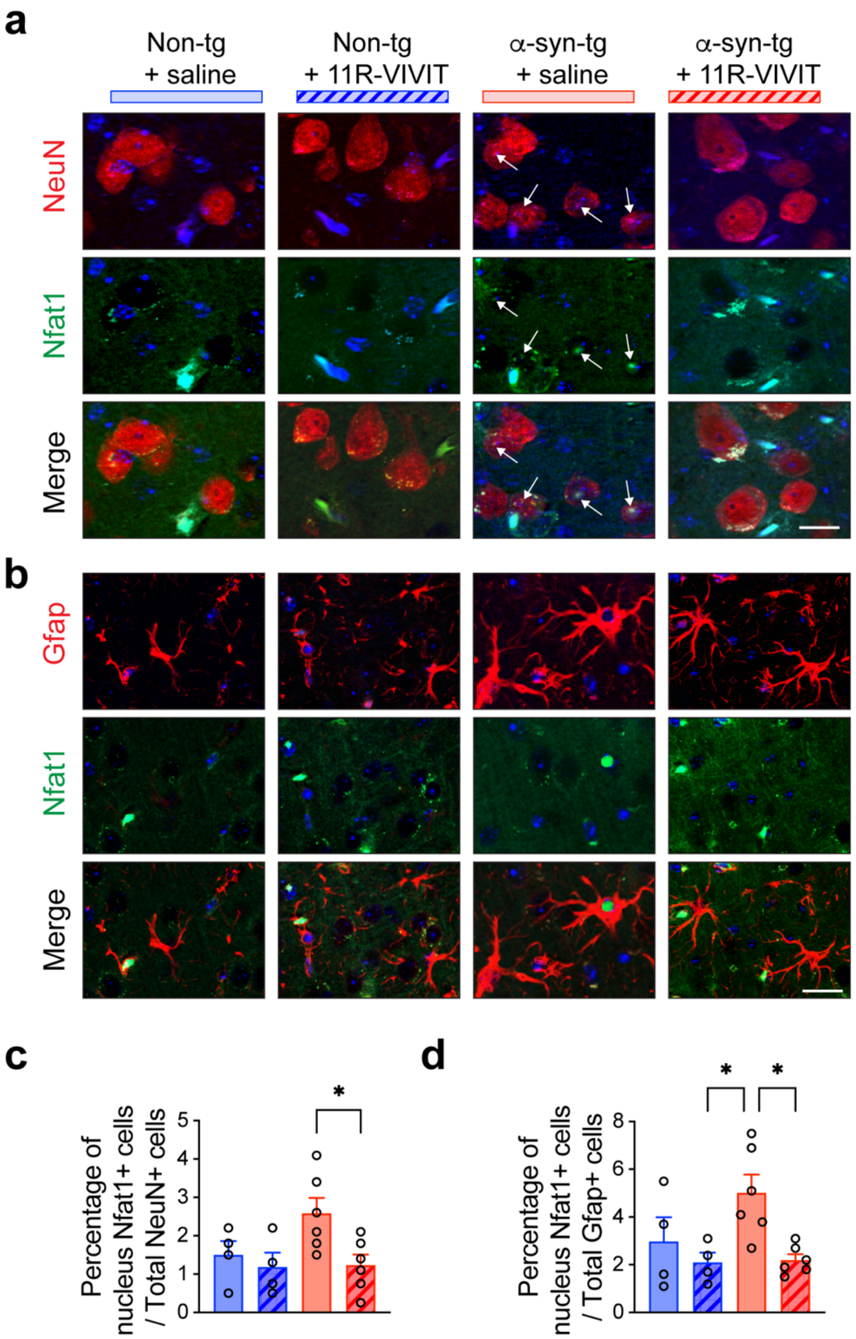

Supplementary Fig. 3 Level of nuclear NFAT1 in brain cells. **a**, **b** Representative images from double-immunolabeling for Nfat1, neuronal marker NeuN, (**a**) and astrocyte marker Gfap (**b**). Arrows indicate nuclear Nfat1. Scale bar, 20 mm. **c**, **d** Percentages of nuclear Nfat1-positive cells among NeuN-positive neurons (**c**) and Gfap-positive astrocytes (**d**) in the neocortices of the mice (n = 4 or 6 per group). Data are shown as means ± SEM. *: $p < 0.05$. Two-way ANOVA and Tukey's multiple comparison test.

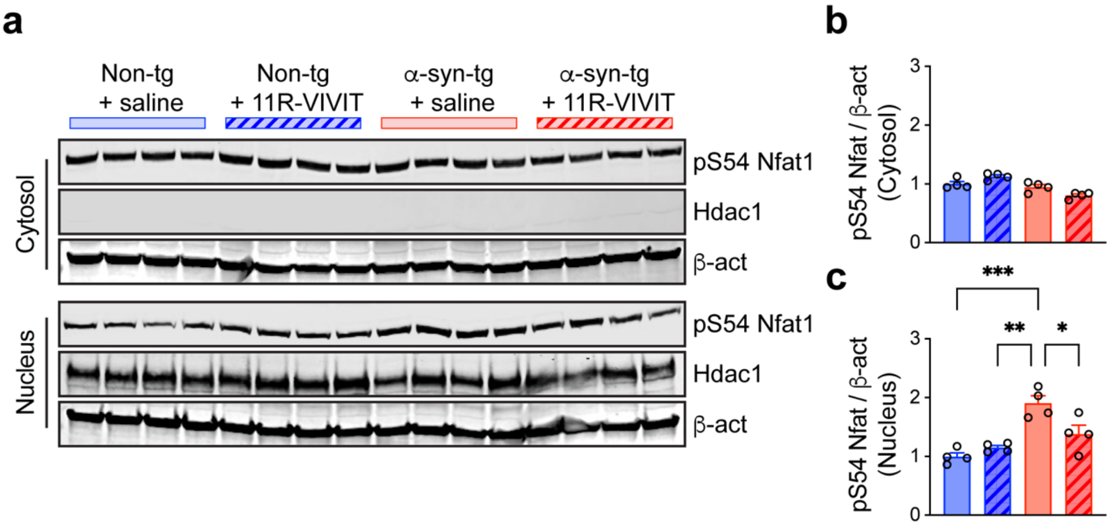

Supplementary Fig. 4 Level of cytosolic and nucleus phosphor-S54 NFAT1 in mice brains. **a** Immunoblot analysis of cytosolic and nucleus fractions of the mice. The blots were probed with phosphor-S54 Nfat1, nucleus marker Hdac1, and β-actin. **b**, **c** The levels of cytosolic (**b**) and nucleus (**c**) phosphor-S54 Nfat1 were determined by densitometric quantification and normalized to β-actin (n = 4 per group). Data are shown as means ± SEM. *: $p < 0.05$; **: $p < 0.01$; ***: $p < 0.001$. Two-way ANOVA and Tukey's multiple comparison test.

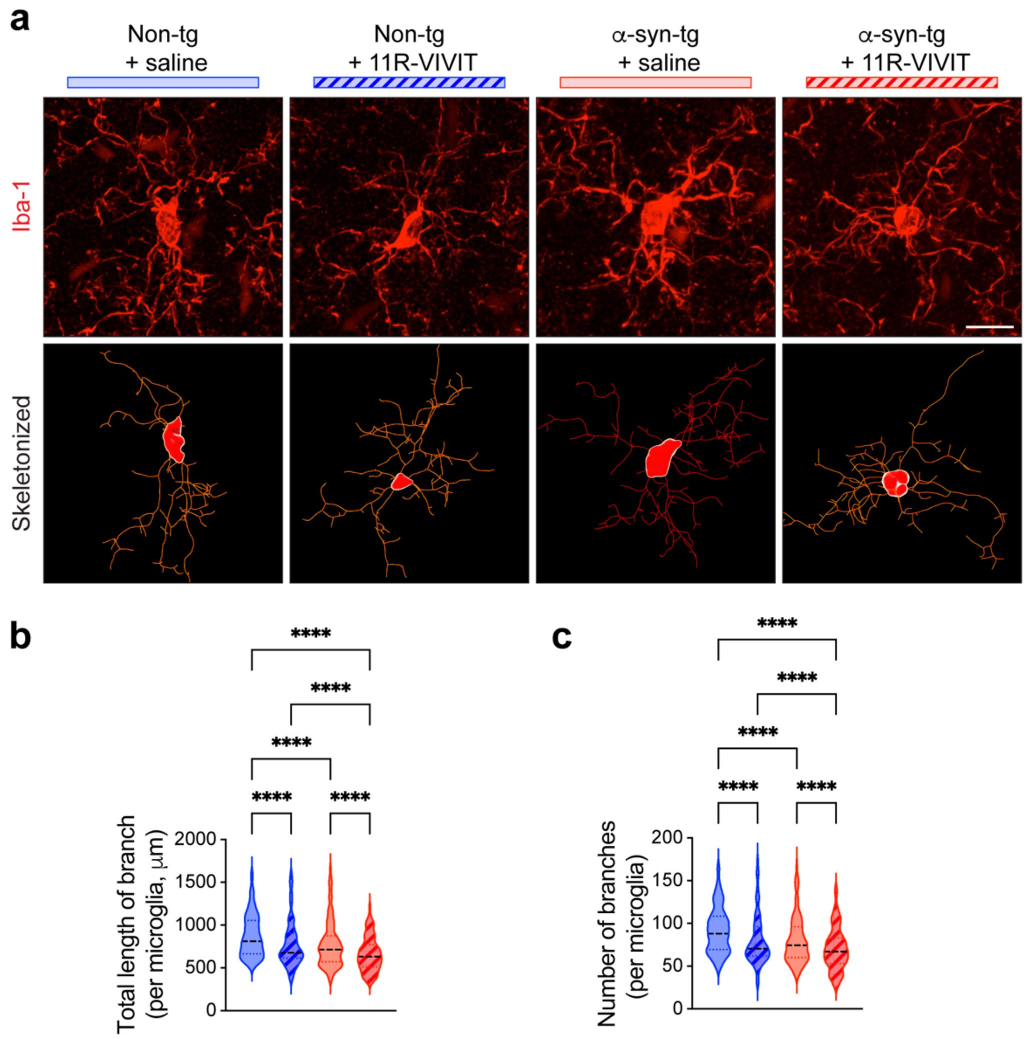

Supplementary Fig. 5 3D *in vivo* microglia phenotype analysis. a Representative images from immunolabeling for Iba-1. 3D reconstructed representative cell images (upper panels) with their matching skeletonized images (lower panels). Scale bar, 10 mm. **b**, **c** Total length (**b**) and number (**c**) of microglial branches were determined by Bitplane Imaris (n = 45, 60, 90, 75 per group). Data are shown as means ± SEM. ****: p < 0.0001. Two-way ANOVA and Tukey's multiple comparison test.

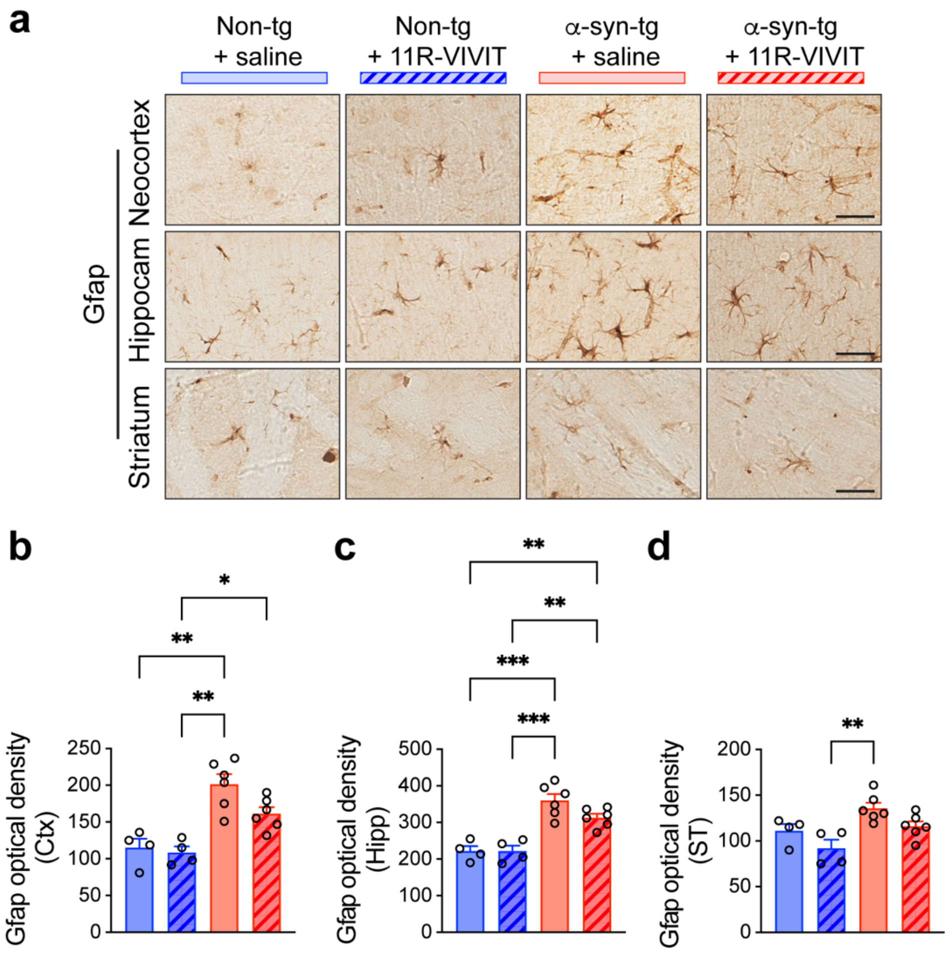

Supplementary Fig. 6 Inhibition of NFAT1 reduced astrogliosis. a Representative images from Gfap immunohistochemical staining in the neocortex, hippocampus, and striatum of the mice. Scale bar, 25 mm. **b-d** The level of Gfap was analyzed by optical density quantification in the neocortex (**b**), hippocampus (**c**), and striatum (**d**) (n = 4 or 6 per group). Data are shown as means ± SEM. *: $p < 0.05$; **: $p < 0.01$; ***: $p < 0.001$. Two-way ANOVA and Tukey's multiple comparison test.

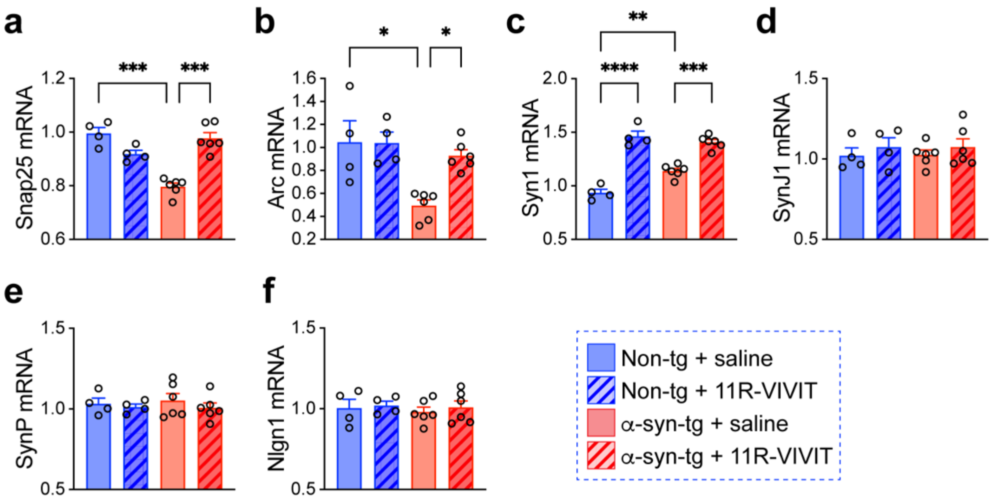

Supplementary Fig. 7 The expression of pre- and post- synaptic genes in a mouse model of synucleinopathy. Quantitative synaptic gene expression analysis from injected non-tg and α-syn-tg mice. **a-f** Expressions of Snap25 (**a**), Arc (**b**), Syn1 (**c**), SynJ1 (**d**), SynP (**e**), and Nlgn1 (**f**) were determined by quantitative PCR (n = 4 or 6 per group). Data are shown as means ± SEM. *: $p < 0.05$; **: $p < 0.01$; ***: $p < 0.001$; ****: $p < 0.0001$. Two-way ANOVA and Tukey's multiple comparison test.

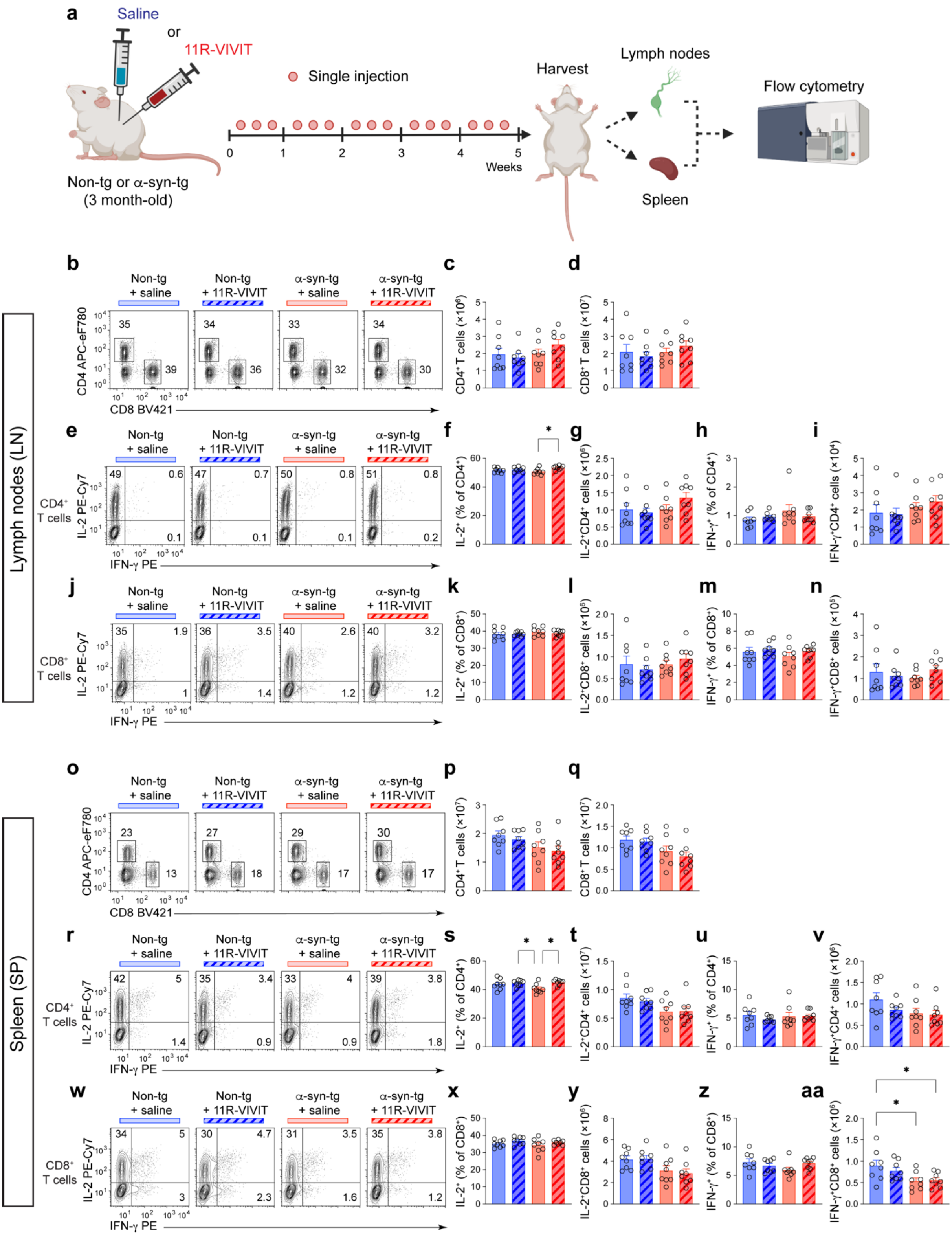

Supplementary Fig. 8 Functional analysis of peripheral T cells. a Experimental scheme. Non-tg and α-syn-tg mice were administered either saline or 11R-VIVIT intraperitoneally three times per week for 5 weeks. At the end of the treatment, single-cell suspensions from

the lymph nodes and spleens were stimulated with PMA and ionomycin for four hours, and the ability of the $CD4^+$ T cells and $CD8^+$ T cells to produce IL-2 and IFN-γ was determined by intracellular cytokine staining. **b**-**n** Representative contour plots depict the frequency of $CD4^+$ and $CD8^+$ T cells and IL-2- and IFN-γ-producing cells within $CD4^+$ and $CD8^+$ T cell populations from the lymph nodes of saline- or 11R-VIVIT-treated non-tg and α-syn-tg mice (**b**, **e**, **j**). Bar graphs demonstrate the numbers of $CD4^+$ and $CD8^+$ T cells (**c**, **d**), the frequencies and numbers of IL-2- and IFN-γ-producing $CD4^+$ T cells (**f**-**i**), and the frequencies and numbers of $CD8^+$ T cells (**k**-**n**) in the lymph nodes (n = 8 per group). Data are shown as means ± SEM. *: $p < 0.05$. Two-way ANOVA and Tukey's multiple comparison test. **o-aa** Representative contour plots depict the frequency of $CD4^+$ and $CD8^+$ T cells and IL-2- and IFN-γ-producing cells within $CD4^+$ and $CD8^+$ T cell populations from the spleens of saline- and 11R-VIVIT-treated non-tg and α-syn-tg mice (**o**, **r**, **w**). Bar graphs demonstrate the numbers of $CD4^+$ and $CD8^+$ T cells (**p**, **q**), the frequencies and numbers of IL-2- and IFN-γ-producing $CD4^+$ T cells (**s**-**v**), and the frequencies and numbers of $CD8^+$ T cells (**x**-**aa**) in the spleen. (n = 8 per group). Data are shown as means ± SEM. *: $p < 0.05$. Two-way ANOVA and Tukey's multiple comparison test.

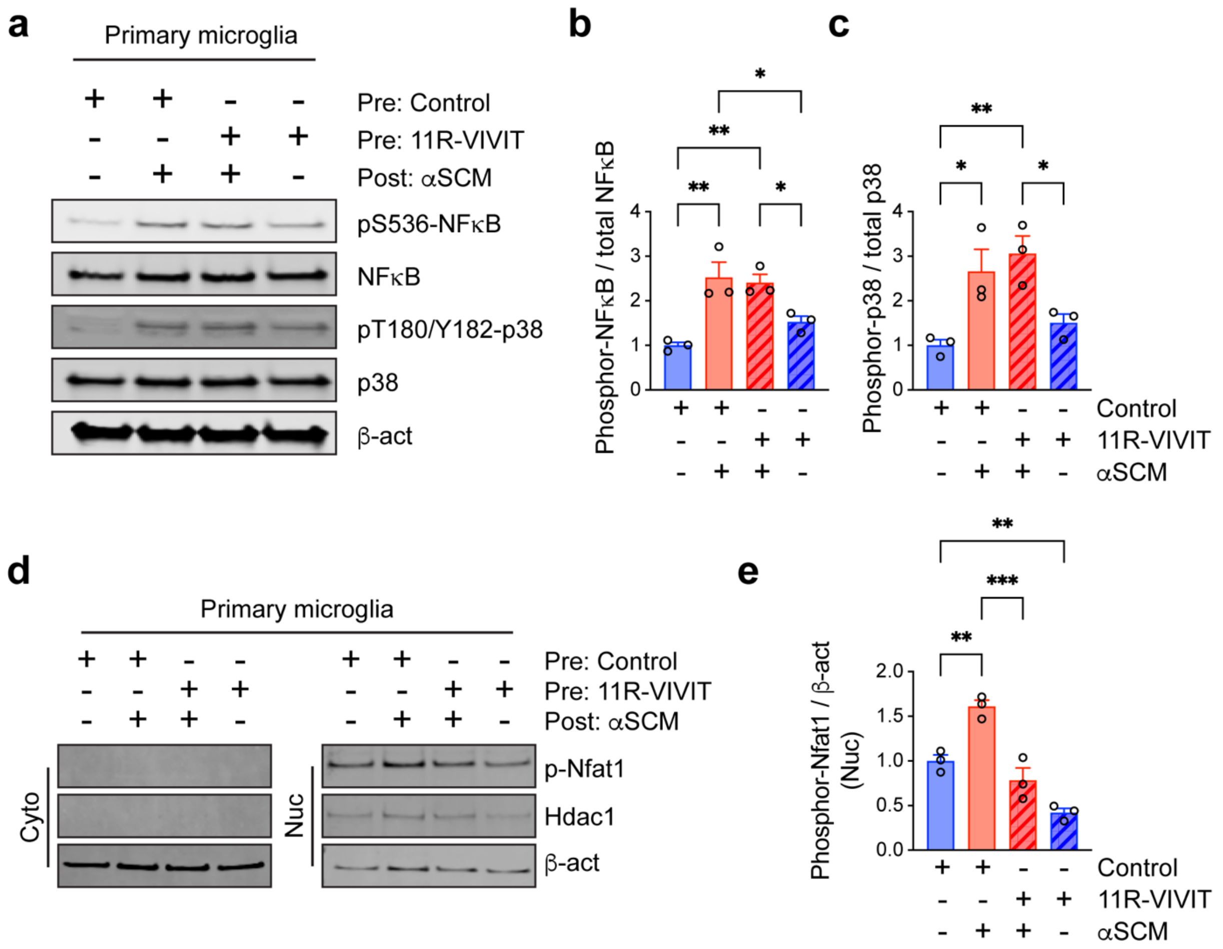

Supplementary Fig. 9 Inhibition of NFAT1 reduced nuclear translocation of NFAT1 through α-syn, but had no effect on the activations of NFkB and p38 MAPK by α-syn in microglia. a Primary mouse microglia were pre-treated with either control or 11R-VIVIT for 1 hour, followed by control LZCM or neuron-released α-syn (αSCM) for 2 hours. Immunoblot analysis of whole cell lysates. The transferred blot was probed for total NFkB, phosphor-NFkB (S536), total p38 MAPK, phosphor-p38 MAPK (T180/Y182), and β-actin. **b**, **c** The levels of phosphor-NFkB (**b**) and phosphor-p38 (**c**) were determined by densitometric quantification and normalized to total NFkB and p38, respectively (n = 3 per group). Data are shown as means ± SEM. *: $p < 0.05$; **: $p < 0.01$. Two-way ANOVA and Tukey's multiple comparison test. **d** Immunoblot analysis of soluble proteins in the nuclear and cytoplasmic fractions of the microglia. The transferred blot was probed for phosphor-S54 Nfat1, Hdac1, and β-actin. **e** The level of phosphor-Nfat1 was determined by densitometric quantification and normalized to β-actin (n = 3 per group). Data are shown as means ± SEM. **: $p < 0.01$; ***: $p < 0.001$. Two-way ANOVA and Tukey's multiple comparison test.

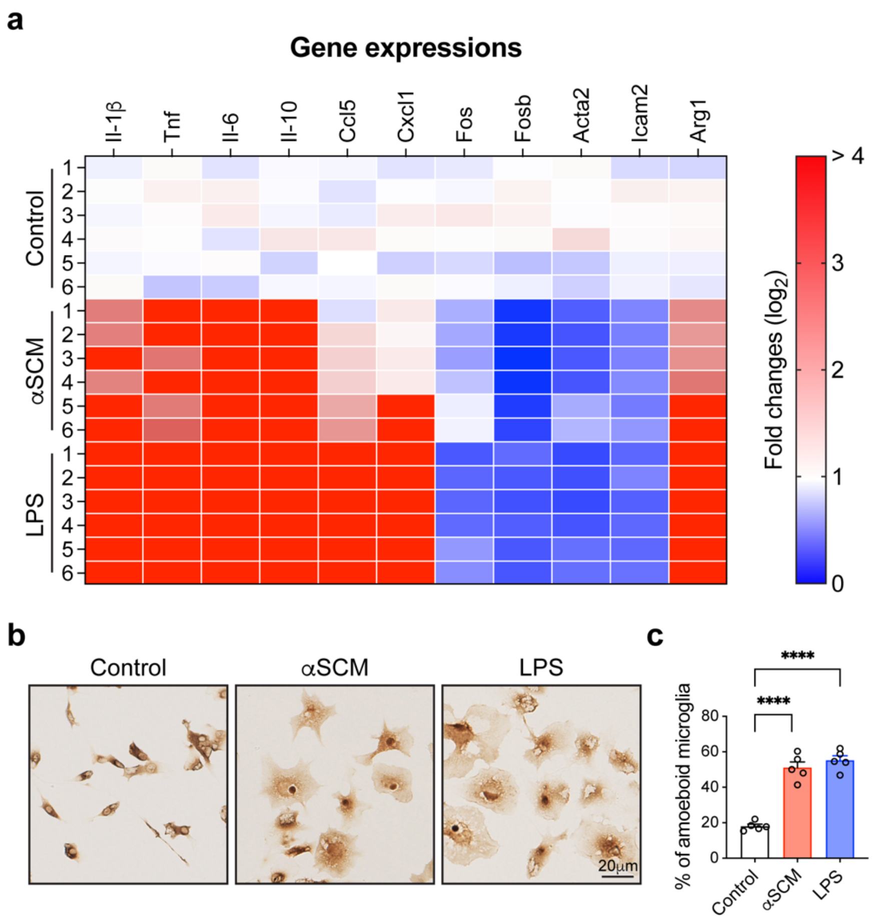

Supplementary Fig. 10 Comparison of microglia excessively activated by either αSCM or LPS. a Wildtype mouse primary microglia were treated with control, αSCM, or LPS (200 ng/ml) for 24 hours. Total RNA was extracted and gene expressions were analyzed by quantitative PCR. Target genes associated with inflammation, migration, and phagocytosis were selected according to the transcriptome data analysis in Fig. 4 (*n* = 6 per group). **b**, **c** Microglia were also immunohistochemically stained against Iba-1 (**b**) and percentage of amoeboid microglia (**c**) was calculated (*n* = 5 per group). Data are shown as means ± SEM. ****: $p < 0.0001$. Two-way ANOVA and Tukey's multiple comparison test.

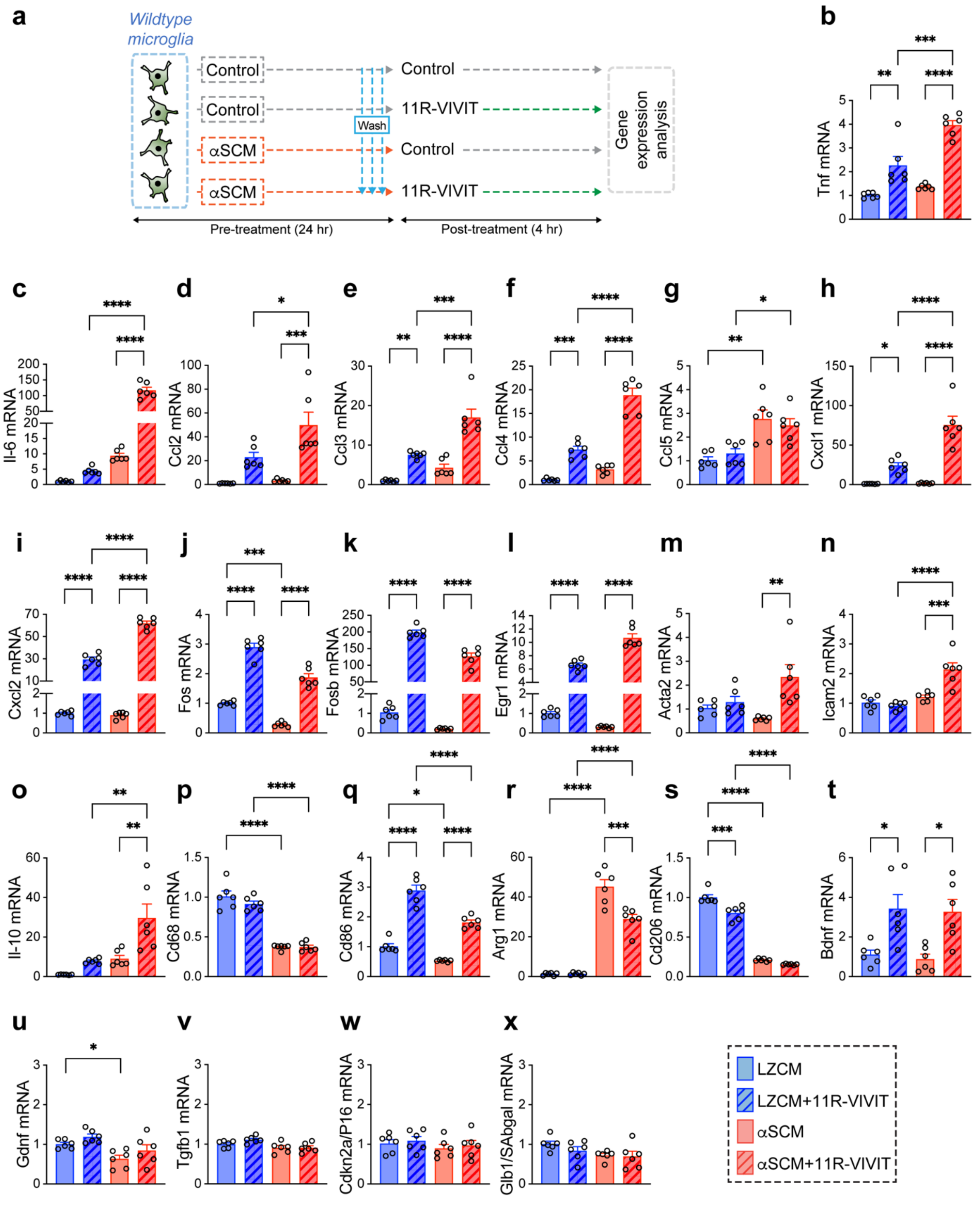

Supplementary Fig. 11 Profiling microglia states. **a** Experimental scheme. Primary wildtype mouse microglia were pretreated with either LZCM or αSCM to obtain control and excessively activated microglia. After a 24-hour pre-treatment, the microglia were incubated with or without 11R-VIVIT (1 mM) for an additional 4 hours. **b**-**x** Microglial expressions of Tnf (**b**), Il-6 (**c**), Ccl2 (**d**), Ccl3 (**e**), Ccl4 (**f**), Ccl5 (**g**), Cxcl1 (**h**), Cxcl2 (**i**), Fos (**j**), Fosb (**k**),

Egr1 (**l**), Acta2 (**m**), Icam2 (**n**), Il-10 (**o**), Cd68 (**p**), Cd86 (**q**), Arg1 (**r**), Cd206 (**s**), Bdnf (**t**), Gdnf (**u**), Tgfb1 (**v**), Cdkn2a/P16 (**w**), and Glb1/SAbgal (**x**) were determined by quantitative PCR ($n = 6$ per group). Data are shown as means ± SEM. *: $p < 0.05$; **: $p < 0.01$; ***: $p < 0.001$; ****: $p < 0.0001$. One-way ANOVA and Tukey's multiple comparison test.

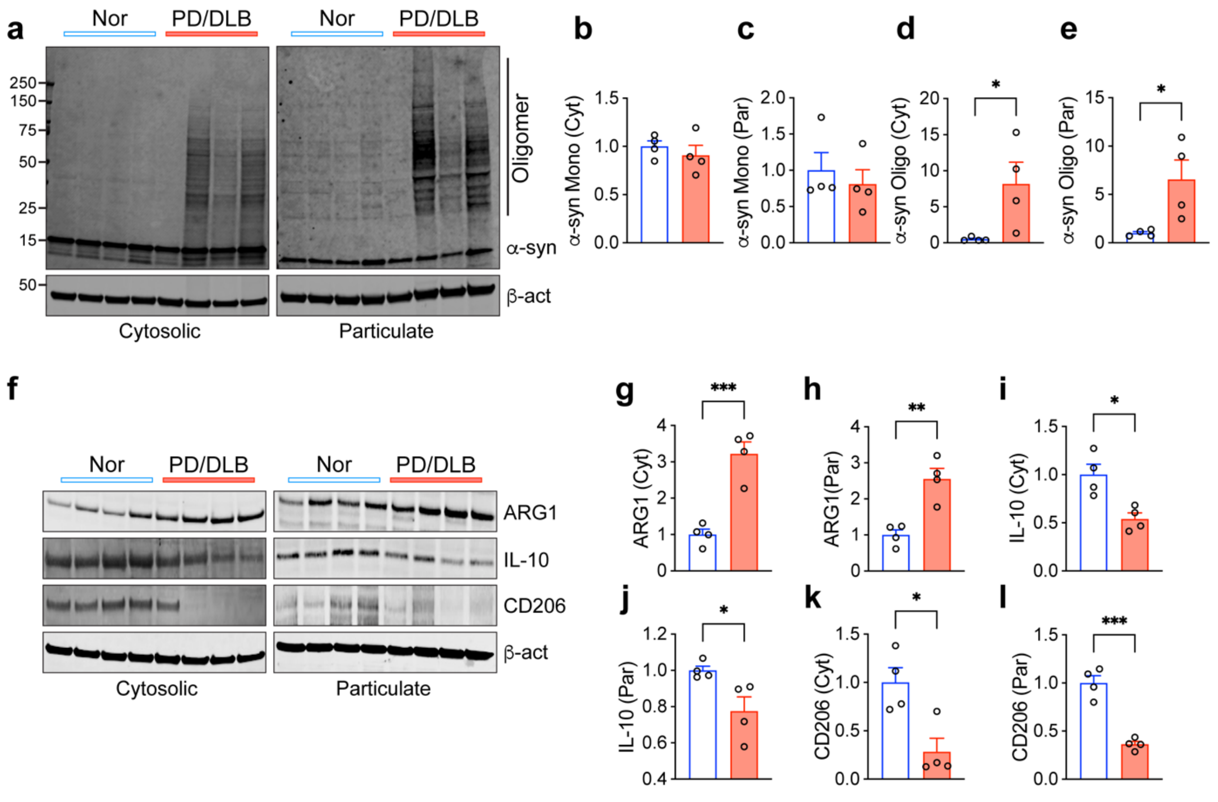

Supplementary Fig. 12 Immunoblot analysis of human brains. **a** Immunoblots of cytosolic and particulate fractions of normal and PD/DLB brain homogenates probed for α-syn and β-actin. **b**-**e** Levels of cytosolic α-syn monomer (**b**), particulate α-syn monomer (**c**), cytosolic α-syn oligomer (**d**), and particulate α-syn oligomer (**e**) were determined by densitometric quantification and normalized to β-actin (n = 4 per group). Data are shown as means ± SEM. *: $p < 0.05$. Unpaired t test. **f** Cytosolic and particulate fractions of normal and PD/DLB brain homogenates were probed for ARG1, IL-10, CD206, and β-actin. **g**-**l** Levels of cytosolic ARG1 (**g**), particulate ARG1 (**h**), cytosolic IL-10 (**i**), particulate IL-10 (**j**), cytosolic CD206 (**k**), and particulate CD206 (**l**) were determined by densitometric quantification and normalized to β-actin (n = 4 per group). Data are shown as means ± SEM. *: $p < 0.05$; **: $p < 0.01$; ***: $p < 0.001$. Unpaired t test.

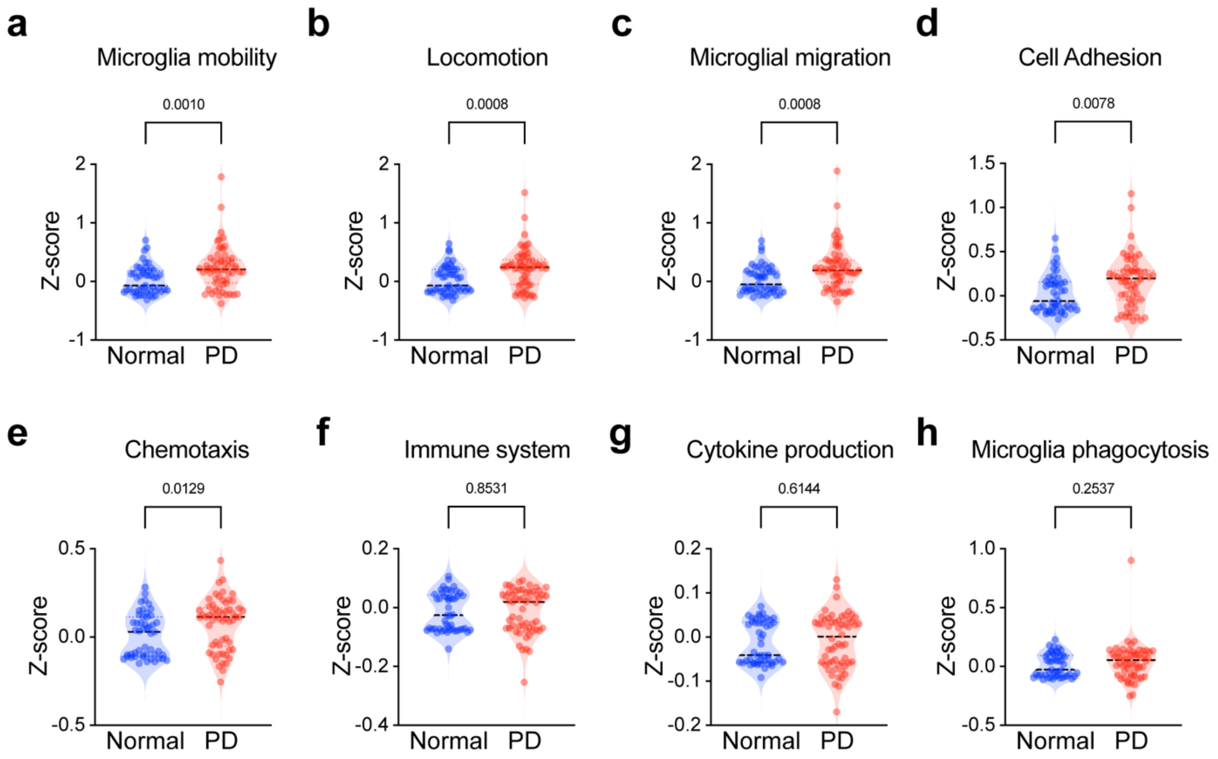

Supplementary Fig. 13 Transcriptional signature activity in the brains of normal and PD patients. a-h Violin plots indicating distribution of transcriptional signature activities in the brains of normal and PD patients. Each point indicates a Z-score of the sample. Horizontal lines indicate the means of each sample of Z-scores. Signature activities include microglial mobility (**a**), locomotion (**b**), microglial migration (**c**), cell adhesion (**d**), chemotaxis (**e**), immune system (**f**), cytokine production (**g**), and microglia phagocytosis (**h**) (n = 49 or 58 per group). Data are shown as means ± SEM. p values were indicated in the graph. Unpaired t test.

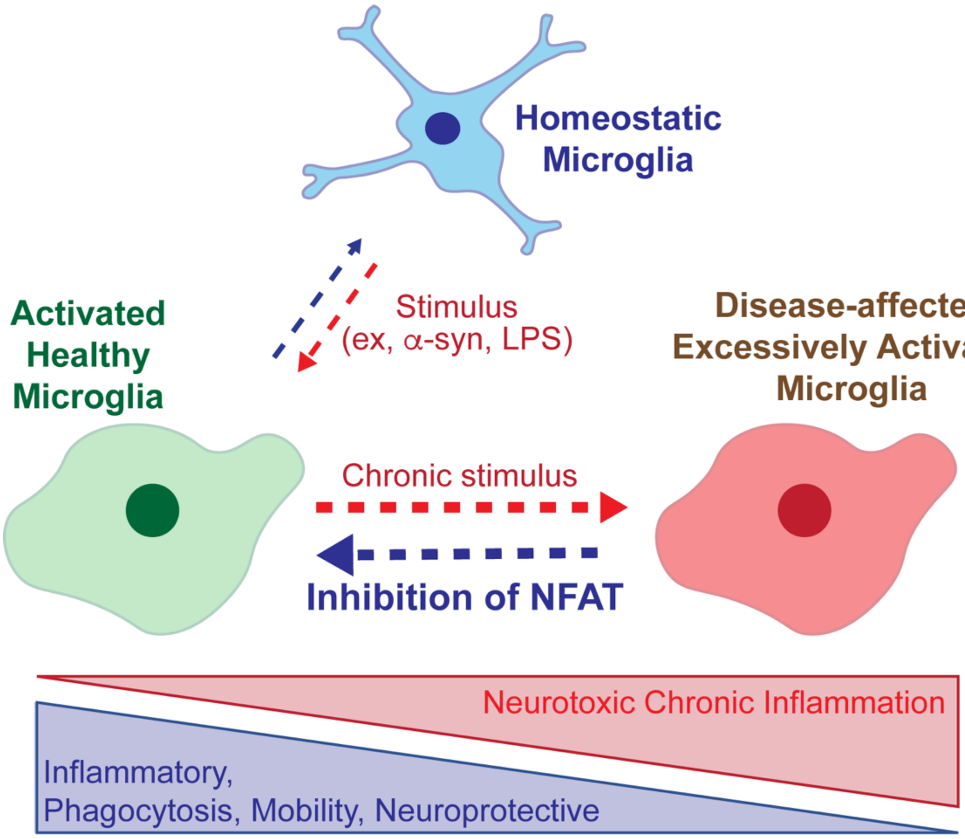

Supplementary Fig. 14 Schematic describing a model for NFAT1 inhibition restoring microglia from excessively activated to healthily active in synucleinopathies. In response to various stimuli, homeostatic microglia become activated, inducing their rapid migration to the damaged site. Activated microglia directly remove pathogens via phagocytosis and produce cytokines, chemokines, and neurohormones that elevate the immune response, in doing so also protecting neighboring neurons. However, chronic activation pushes "healthily active microglia" to become "excessively activated," characterized by production of large amounts of neurotoxic inflammatory cytokines and chemokines and suppression of their neuroprotective abilities like pathogen phagocytosis. We demonstrate that the functional inhibition of NFAT1 restores these "excessively activated microglia" to "activated healthy microglia", thereby reducing their production of inflammatory cytokines and chemokines and revesting their migration and phagocytic abilities.

Supplementary Table 1 List of antibodies for current study. The table shows comprehensive information on antibodies used. From left to right: Target gene, specificity, host species, dilutions, source, catalog number, and notes as applicable.

Antibody name	Host species	Dilution	Source	Cat NO.	Note
Anti-α-syn	Mouse	1:500 (IHC)<br>1:1K (IB)	BD Biosciences	610787	Syn1,<br>Total α-synuclein
Anti-ARG1	Rabbit	1:10K (IB)	Proteintech	16001-1-AP	-
Anti-β-actin	Mouse	1:1K (IB)	Millipore	A5441	-
Anti-β-actin	Rabbit	1:1K (IB)	Cell Signaling	4967	-
Anti-CD206	Rabbit	1:1K (IB)	Proteintech	18704-1-AP	-
Anti-GFAP	Mouse	1:500 (IHC, IF)	Millipore	MAB3402	Astrocyte marker
Anti-Hdac1	Rabbit	1:1K (IB)	Invitrogen	PA1-860	
Anti-Iba-1	Rabbit	1:500 (IHC, IF)	Fujifilm Wako	019-19741	Microglia marker
Anti-IL-10	Rabbit	1:1K (IB)	Invitrogen	PA5-85660	
Anti-LRRK2	Rabbit	1:1K (IB)	Abcam	ab133474	MJFF2 (c41-2)
Anti-NeuN	Mouse	1:500 (IHC, IF)	Millipore	MAB377	Neuronal marker
Anti-NFAT1	Rabbit	1:500 (IB)	Boster	PA1664	For human NFAT1
Anti-NFAT1	Mouse	1:1K (IB, IHC, IF)	Invitrogen	MA1-025	
Anti-NFkB p65	Rabbit	1:1K (IB)	Cell Signaling	8242	
Anti-p38 MAPK	Rabbit	1:1K (IB)	Cell Signaling	9211	Total p38 MAPK
Anti-phosphor-α-syn (81A)	Mouse	1:10K (IHC)	-	-	phosphor-S129, Gifted from Drs. Lee and Trojanowski
Anti-phosphor-NFAT1	Rabbit	1:1K (IB)	Invitrogen	44-944G	phosphor-S54
Anti-phosphor-NFAT1 (C9)	Mouse	1:100 (IB)	-	-	Generated with GenScript
Anti-phosphor-NFkB p65	Rabbit	1:1K (IB)	Cell Signaling	3033	phosphor-S536
Anti-phosphor-p38 MAPK	Rabbit	1:1K (IB)	Cell Signaling	9219	phosphor-T180 /Y182
Anti-TH	Rabbit	1:500 (IB)	Millipore	AB152	Tyrosine hydroxylase

Anti-V5	Mouse	-	Thermo Fisher Scientific	R96025	For custom ELISA
Anti-V5	Rabbit	-	Thermo Fisher Scientific	PA1-993	For custom ELISA
CD4 APC-eFluor 780	Rat	1:100	Invitrogen	RM4-5	For FACS analysis
CD8 Pacific Blue	Rat	1:100	Invitrogen	5H10	For FACS analysis
IL-2 PE-Cyanine7	Rat	1:100	Invitrogen	JES6-5H4	For FACS analysis
IFN-γ PE	Rat	1:100	BioLegend	XMG1.2	For FACS analysis
IRDye® 800CW Goat anti-Rabbit IgG (H + L)	Goat	1:20K (IB)	LI-COR Biosciences	925-32211	Detection antibody for IB
IRDye® 800CW Goat anti-Mouse IgG (H + L)	Goat	1:20K (IB)	LI-COR Biosciences	925-32210	Detection antibody for IB
IRDye® 680RD Goat anti-Rabbit IgG (H + L)	Goat	1:20K (IB)	LI-COR Biosciences	925-68071	Detection antibody for IB
IRDye® 680RD Goat anti-Mouse IgG (H + L)	Goat	1:20K (IB)	LI-COR Biosciences	925-68070	Detection antibody for IB
Fluorescein Horse Anti-Mouse IgG	Horse	1:500 (IF)	Vector laboratories	FI-2000	Detection antibody for IF
Fluorescein Goat Anti-Rabbit IgG	Goat	1:500 (IF)	Vector laboratories	FI-1000	Detection antibody for IF
Texas Red® Goat Anti-Rabbit IgG	Goat	1:500 (IF)	Vector laboratories	TI-1000	Detection antibody for IF
Texas Red® Horse Anti-Mouse IgG	Horse	1:500 (IF)	Vector laboratories	TI-2000	Detection antibody for IF
Biotinylated Horse Anti-Mouse IgG (H+L)	Horse	1:100 (IHC)	Vector laboratories	BA-2000-1.5	Detection antibody for IHC
Biotinylated Goat Anti-Rabbit IgG	Goat	1:100 (IHC)	Vector laboratories	BA-1000-1.5	Detection antibody for IHC

Supplementary Table 2 List of animals for current study. The table shows the information of animals (Cohorts 1 to 3). From left to right: Group, mouse ID, and sex.

Cohort 1			Cohort 2			Cohort 3		
**Group**	**Mouse ID**	**Sex**	**Group**	**Mouse ID**	**Sex**	**Group**	**Mouse ID**	**Sex**
Non-tg + Saline	168	M	Non-tg + Saline	539	F	Non-tg + Saline	1105	F
	176	F		540	F		1116	F
	177	F		542	M		1117	F
	189	F		543	M		1118	F
Non-tg + 11R-VIVIT	146	F	Non-tg + 11R-VIVIT	535	M		1122	F
	157	F		536	M		1129	M
	158	F		537	M		1130	M
	160	F		541	M		1137	M
α-syn-tg + Saline	169	M	α-syn-tg + Saline	547	F	Non-tg + 11R-VIVIT	1131	F
	170	M		549	F		1132	F
	185	F		554	M		1146	F
	186	F		555	M		1147	F
	187	F	α-syn-tg + 11R-VIVIT	534	M		1102	M
	188	F		538	F		1103	M
α-syn-tg + 11R-VIVIT	136	F		544	M		1115	M
	137	F		546	F		1121	M
	147	F				α-syn-tg + Saline	1106	F
	148	F					1107	F
	150	F					1123	F
	180	M					1124	F
							1134	M
							1135	M
							1136	M
							1138	M
						α-syn-tg + 11R-VIVIT	1099	F
							1100	F
							1101	F
							1133	F
							1104	M
							1114	M
							1119	M
							1120	M

Supplementary Table 3 List of primers for quantitative PCR. The table shows the information on primers. From left column to right: ***i***) Target gene; ***ii***) Source; and ***iii***) Catalog number.

Target gene	Source	Cat NO.
Mouse Tnfα	Thermo Fisher Scientific	Mm00443258
Mouse Il-6	Thermo Fisher Scientific	Mm00446190
Mouse Il-1β	Thermo Fisher Scientific	Mm00434228
Mouse Il-10	Thermo Fisher Scientific	Mm01288386
Mouse Cxcl1	Thermo Fisher Scientific	Mm04207460
Mouse Cxcl2	Thermo Fisher Scientific	Mm00436450
Mouse Ccl2	Thermo Fisher Scientific	Mm00441242
Mouse Ccl3	Thermo Fisher Scientific	Mm99999057
Mouse Ccl4	Thermo Fisher Scientific	Mm00443111
Mouse Ccl5	Thermo Fisher Scientific	Mm01302427
Mouse Egr1	Thermo Fisher Scientific	Mm00456650
Mouse Fos	Thermo Fisher Scientific	Mm00487425
Mouse Fosb	Thermo Fisher Scientific	Mm00500401
Mouse Acta2	Thermo Fisher Scientific	Mm01546133
Mouse Icam2	Thermo Fisher Scientific	Mm00494862
Mouse Arg1	Thermo Fisher Scientific	Mm00475988
Mouse Cd68	Thermo Fisher Scientific	Mm03047343
Mouse Cd86	Thermo Fisher Scientific	Mm01344638
Mouse Cd206	Thermo Fisher Scientific	Mm01329362
Mouse Bdnf	Thermo Fisher Scientific	Mm01334042
Mouse Gdnf	Thermo Fisher Scientific	Mm00599849
Mouse Tgfb1	Thermo Fisher Scientific	Mm01178820
Mouse Cdkn2a/p16	Thermo Fisher Scientific	Mm00494449
Mouse Glb1/SAbgal	Thermo Fisher Scientific	Mm00515342
Mouse Syn1	Thermo Fisher Scientific	Mm00449772
Mouse SynJ1	Thermo Fisher Scientific	Mm01210539

Mouse Snap25	Thermo Fisher Scientific	Mm01276449
Mouse SynP	Thermo Fisher Scientific	Mm00436850
Mouse Nlgn1	Thermo Fisher Scientific	Mm02344307
Mouse Arc	Thermo Fisher Scientific	Mm01204954
Mouse β-actin	Thermo Fisher Scientific	Mm00607939
Human TNFα	Thermo Fisher Scientific	Hs00174128
Human IL-6	Thermo Fisher Scientific	Hs00174131
Human IL-1β	Thermo Fisher Scientific	Hs01555410
Human CCL5	Thermo Fisher Scientific	Hs00982282
Human CXCL5	Thermo Fisher Scientific	Hs01099660
Human CX3CL1	Thermo Fisher Scientific	Hs00171086
Human FOS	Thermo Fisher Scientific	Hs04194186
Human FOSB	Thermo Fisher Scientific	Hs00171851
Human EGR1	Thermo Fisher Scientific	Hs00152928
Human ACTA2	Thermo Fisher Scientific	Hs05005341
Human ICAM2	Thermo Fisher Scientific	Hs00609563
Human RCAN1	Thermo Fisher Scientific	Hs01120954
Human β-actin	Thermo Fisher Scientific	Hs03023880

Supplementary Table 4 Human specimens, neuropathological evaluation and criteria for diagnosis. The table shows information on human specimens. From left column to right: ***i***) Diagnosis; ***ii***) number; ***iii***) Age; ***iv***) Gender; ***v***) Disease duration; ***vi***) Hemi-brain weight; ***vii***) Postmortem time; and ***viii***) Braak stage.

Group	N	Age (yrs)	Gender (M/F)	Disease duration (yrs)	Hemi Brain Weight (grs)	PMT (hrs)	Braak stage
Control	9	86.2 ± 11.4	4/5	NA	542.1 ± 48.76	20 ± 21.9	1.0 ± 0.50
PD/DLB	12	77.6 ± 2.41	7/5	11.3 ± 1.32	581.5 ± 30.73	11 ± 1.8	4.9 ± 0.51

Supplementary Table 5 A gene list of clustered differentially expressed genes. The table shows information on the genes grouped by cluster.

Entrez id	Symbol	Cluster	α-syn-tg-Veh vs. Non-tg-Veh			α-syn-tg-11R-VIVIT vs. α-syn-tg-Veh		
			log2 (FC)	p-value	FDR	log2 (FC)	p-value	log2 (FC)
14744	Gpr65	C1	0.451242	6.3475E-05	0.001786	-0.45597	0.007638	0.041817
217830	Dglucy	C1	0.54869	6.6437E-05	0.001825	-0.45857	0.000219	0.006905
100862085	Gm16867	C1	0.459432	0.00970245	0.036193	-0.45943	0.006226	0.037604
12774	Ccr5	C1	0.480224	2.6142E-06	0.000457	-0.47474	3.59E-05	0.004137
240444	Kcng2	C1	0.505742	0.01529944	0.049218	-0.47534	0.001361	0.01608
100038735	Gm10384	C1	0.485427	0.00565672	0.025167	-0.48543	0.000741	0.012127
102595	Plekho2	C1	0.671865	7.4561E-06	0.000645	-0.48704	0.005834	0.03615
269799	Clec4a1	C1	0.495301	0.00027285	0.003726	-0.4953	0.000481	0.009831
672195	Gm10053	C1	0.453445	0.00025073	0.003622	-0.5	0.002379	0.021535
80910	Gpr84	C1	0.581039	0.00014864	0.002758	-0.51212	0.001044	0.014182
20971	Sdc4	C1	0.698732	0.00013446	0.002638	-0.51881	0.000243	0.007107
619900	Rps27a-ps2	C1	0.529447	0.00728441	0.030055	-0.52945	0.001226	0.015314
13537	Dusp2	C1	0.653052	4.1129E-06	0.000482	-0.53255	0.003341	0.0263
12263	C2	C1	0.535195	0.00297884	0.016456	-0.53519	0.002798	0.023557
68709	Cilp2	C1	0.642993	2.5793E-05	0.001111	-0.55414	0.000219	0.006888
56485	Slc2a5	C1	0.49799	0.00116621	0.008931	-0.56255	4.87E-05	0.004455
15982	Ifrd1	C1	0.87232	6.7841E-07	0.000419	-0.57824	0.001291	0.015793
18175	Nrap	C1	0.584963	6.6704E-05	0.001822	-0.58496	0.004797	0.032255
18037	Nfkbie	C1	0.548835	9.2486E-07	0.000343	-0.58919	0.000223	0.006941
269959	Adamtsl3	C1	0.631517	0.00169675	0.011375	-0.59632	0.00697	0.03995
414801	Itprip	C1	0.611196	8.3347E-05	0.00201	-0.6087	0.002717	0.02326
12363	Casp4	C1	0.656683	5.517E-05	0.001637	-0.6112	0.000873	0.01318
75188	1700009J07Rik	C1	0.670785	0.0029664	0.016416	-0.61393	0.006511	0.038446
18106	Cd244a	C1	0.5	0.00265968	0.015182	-0.63152	0.000593	0.01067
67951	Tubb6	C1	0.685305	0.00011402	0.002394	-0.63982	0.000823	0.012798
664849	Gm7367	C1	0.753829	3.2845E-05	0.001221	-0.64135	5.37E-05	0.004647
384059	Tlr12	C1	0.454716	0.00054948	0.005435	-0.64411	0.000675	0.011462
12475	Cd14	C1	0.629285	3.9248E-06	0.000485	-0.65679	0.0003	0.007822
18036	Nfkbib	C1	0.521619	1.0258E-05	0.000719	-0.65888	0.000104	0.005632
16176	Il1b	C1	1.069753	3.0878E-07	0.000408	-0.66004	0.002505	0.022282
72504	Taf4b	C1	0.5	0.01149457	0.040175	-0.66096	5.84E-05	0.004537
56221	Ccl24	C1	0.812802	0.00039978	0.004592	-0.68129	0.000112	0.005642
74145	F13a1	C1	0.763593	0.00021757	0.003291	-0.68618	1.31E-06	0.001067
73316	Calr3	C1	0.692457	0.00131107	0.009588	-0.69398	0.001344	0.015943
629820	Gm7008	C1	0.584963	0.01387659	0.046132	-0.71423	0.003246	0.025904
18124	Nr4a3	C1	1.192716	1.27E-06	0.000392	-0.72029	0.00066	0.011333

21950	Tnfsf9	C1	0.835346	1.9702E-06	0.000424	-0.73902	0.00101	0.013996
17710	COX3	C1	0.705873	6.1154E-05	0.001742	-0.7561	0.004241	0.03009
12047	Bcl2a1d	C1	1.12649	6.9949E-07	0.00036	-0.78745	0.002559	0.022576
114332	Lyve1	C1	0.814016	2.1254E-05	0.001025	-0.7978	3.22E-06	0.001432
12768	Ccr1	C1	0.61323	0.00021733	0.003293	-0.80106	7.87E-05	0.00484
54720	Rcan1	C1	0.946762	1.7811E-07	0.00055	-0.80956	3.18E-05	0.003941
17133	Maff	C1	0.979679	6.0679E-07	0.000432	-0.86848	8.59E-05	0.005062
12051	Bcl3	C1	0.777608	1.5412E-05	0.000897	-0.8888	5.05E-05	0.00441
20310	Cxcl2	C1	0.76966	2.3028E-07	0.000426	-0.92194	2.52E-05	0.003732
56792	Stap1	C1	0.868483	2.5898E-06	0.000461	-0.95345	1.97E-05	0.003328
20556	Slfn2	C1	0.869046	5.182E-07	0.00048	-0.98248	0.002312	0.021421
14825	Cxcl1	C1	0.736966	1.7093E-05	0.000942	-1.02945	2.35E-07	0.000767
20306	Ccl7	C1	0.818715	6.9834E-05	0.001874	-1.03519	1.23E-05	0.002563
19074	Prg2	C1	1.09534	0.00940766	0.035378	-1.04006	0.002551	0.022526
546643	Ly6a2	C1	0.694249	0.0001282	0.002553	-1.06263	3.07E-06	0.001502
231507	Plac8	C1	0.733752	0.0121028	0.041795	-1.08175	0.007799	0.042441
12655	Chil3	C1	0.728257	0.0012805	0.009447	-1.15226	9.1E-05	0.005208
16153	Il10	C1	0.707519	0.00050753	0.005192	-1.26565	8.08E-07	0.001129
12796	Camp	C1	0.662548	0.00361443	0.018581	-1.51277	4.51E-06	0.001697
17002	Ltf	C1	1.003985	0.00326551	0.017456	-1.60921	0.000126	0.005775
18054	Ngp	C1	1.021187	0.00174551	0.011552	-1.92764	3.19E-06	0.001485
68055	Dmac2l	C2	0.207519	0.26212258	0.311896	-0.45023	0.004981	0.033128
215257	Il36g	C2	0.292481	0.05509062	0.115685	-0.45199	0.000909	0.013461
621100	Rpl27-ps3	C2	0.131517	0.20545119	0.270425	-0.45345	0.002648	0.022991
56744	Pf4	C2	0.377444	0.01992903	0.058781	-0.45345	0.000166	0.006326
12569	Cdk5r1	C2	0.314428	0.00276554	0.015632	-0.45425	5.37E-06	0.001695
13664	Eif1a	C2	0.429959	0.00078183	0.00692	-0.45619	0.004433	0.030784
19332	Rab20	C2	0.326629	0.00037465	0.00443	-0.45759	0.004738	0.03199
74748	Slamf8	C2	0.315025	0.00062809	0.005964	-0.46322	0.005308	0.034459
69790	Med30	C2	0.292481	0.05531658	0.116107	-0.46644	0.001577	0.017454
66953	Cdca7	C2	0.345336	0.00084915	0.007327	-0.4668	0.000406	0.00903
246738	Dnajc28	C2	0.080232	0.68013269	0.534414	-0.47154	0.002331	0.021392
14794	Spsb2	C2	0.354476	0.00197665	0.012467	-0.47498	0.003013	0.024714
69142	Cd209f	C2	0.368483	0.03852161	0.091496	-0.47968	0.002725	0.023284
11689	Alox5	C2	0.439496	6.2737E-05	0.001776	-0.48266	0.001522	0.01724
224674	Slc37a1	C2	0.014874	0.59911272	0.501028	-0.48543	0.00621	0.03762
228839	Tgif2	C2	0.431545	5.0447E-05	0.001547	-0.48599	0.001027	0.01405
15205	Hes1	C2	0.393466	0.00020605	0.003217	-0.48673	0.000724	0.011947
12262	C1qc	C2	0.401909	0.00034278	0.004215	-0.48721	0.00082	0.012798
100503794	Gm9920	C2	0.207519	0.02092811	0.060684	-0.5	0.004839	0.032407
102632708	Gm15523	C2	0.207519	0.02092811	0.060627	-0.5	0.004902	0.032668
78802	Ttc30a1	C2	0.181285	0.05176503	0.111224	-0.5	1.59E-06	0.001196

76132	Faxc	C2	0	0.8128989	0.569305	-0.5	0.005306	0.034473
258325	Olfr110	C2	0.207519	0.1461656	0.217568	-0.50447	0.009719	0.048368
12649	Chek1	C2	0.333959	0.00430042	0.020835	-0.50571	0.002278	0.021269
210741	Kcnk12	C2	0.229716	0.03887298	0.091859	-0.51324	0.000623	0.010881
17533	Mrc1	C2	0.286685	0.03928915	0.092324	-0.51544	6.53E-06	0.001995
17357	Marcksl1	C2	0.381852	0.00323861	0.017372	-0.51586	0.000776	0.012464
69156	Comtd1	C2	0.328174	0.01475202	0.047957	-0.52876	0.000499	0.009904
99543	Olfml3	C2	0.425833	2.2007E-05	0.00104	-0.54117	4.27E-05	0.004307
218865	Chdh	C2	-0.13339	0.47463375	0.449508	-0.54655	0.004155	0.029936
60322	Chst7	C2	0.415037	8.2849E-05	0.002013	-0.56986	4.99E-06	0.001684
100504344	Gm20186	C2	0.368483	0.01500602	0.048527	-0.576	0.001315	0.015846
70240	Ufsp1	C2	0.160964	0.23412173	0.293874	-0.576	2.04E-05	0.00332
102639573	Gm35853	C2	0.160964	0.46265564	0.443196	-0.576	0.006873	0.039582
12821	Col17a1	C2	-0.24271	0.3829669	0.396256	-0.576	0.001008	0.013996
15267	H2ac18	C2	-0.29248	0.13433655	0.206604	-0.576	0.003927	0.028891
22271	Upp1	C2	0.292481	0.25286501	0.305913	-0.58496	0.00393	0.028891
75732	Iqcd	C2	0.292481	0.25286501	0.305833	-0.58496	0.003196	0.025629
70989	Jhy	C2	0.292481	0.25286501	0.305793	-0.58496	0.002631	0.022902
545622	Ptpn3	C2	0.292481	0.08389485	0.152093	-0.58496	0.002723	0.023287
319455	Pld5	C2	0.292481	0.25286501	0.305673	-0.58496	3.88E-05	0.004369
545812	Pilrb2	C2	0.292481	0.00432236	0.020876	-0.58496	0.000103	0.005653
100503199	5430416N02Rik	C2	0.292481	0.04878414	0.106676	-0.58496	0.001866	0.018915
100039026	Csnk2a1-ps3	C2	0.292481	0.08513957	0.152853	-0.58496	0.003134	0.025282
115486873	6330537M06Rik	C2	0.292481	0.06660232	0.131004	-0.58496	6.17E-05	0.004605
100503318	Gm15853	C2	0	0.53435172	0.478184	-0.58496	6.17E-05	0.00464
338403	Cndp1	C2	-0.41504	0.02674386	0.071418	-0.58496	0.002392	0.021609
170741	Pilrb1	C2	0.292481	0.00432236	0.020865	-0.5878	8.22E-05	0.004963
74127	Krt80	C2	0.254073	0.16013983	0.230512	-0.59632	0.009099	0.046515
12156	Bmp2	C2	0.437235	0.00136942	0.00989	-0.5982	0.000104	0.005594
17386	Mmp13	C2	0.292481	0.08513957	0.152972	-0.61286	0.000141	0.005887
76206	Gpr165	C2	0.235653	0.26358793	0.313116	-0.61607	0.002727	0.023283
216864	Mgl2	C2	0.297181	0.02144926	0.061788	-0.61852	0.000362	0.008443
16369	Irs3	C2	0	0.77422213	0.561986	-0.61917	5.4E-05	0.004551
100039636	Atp6v0c-ps2	C2	0.292481	0.08389485	0.151915	-0.62967	0.002059	0.020207
235312	C1qtnf5	C2	-0.20752	0.24040359	0.299083	-0.63152	0.006972	0.039915
666684	Gm8237	C2	0.009585	0.94758878	0.600373	-0.6432	0.000334	0.008221
17173	Ascl2	C2	0.298786	0.00818359	0.032463	-0.64835	4.39E-05	0.004251
66107	Wfdc21	C2	0.366799	0.05561381	0.116599	-0.65976	0.000962	0.013766
100504104	Gm16062	C2	0.304263	0.08878364	0.157081	-0.66002	0.001989	0.019736
72962	Tymp	C2	0.311083	0.11266467	0.184099	-0.68203	0.004075	0.029555
108078	Olr1	C2	0.415037	0.00096169	0.007866	-0.70752	0.000112	0.005689
217306	Cd300e	C2	0.292481	0.32411601	0.357497	-0.70752	0.007536	0.041444

76905	Lrg1	C2	0.131517	0.581908	0.493595	-0.70752	0.001302	0.015828
20201	S100a8	C2	-0.00896	0.79898536	0.5662	-0.73697	0.004234	0.030084
16633	Klra2	C2	0.096323	0.17679113	0.246212	-0.74271	8.5E-05	0.005069
56620	Clec4n	C2	0.388804	0.00656494	0.027907	-0.79248	6.58E-05	0.004702
213002	Ifitm6	C2	0.292481	0.07089115	0.135329	-0.79248	0.001137	0.01471
67855	Asprv1	C2	0.207519	0.44517063	0.432851	-0.79248	0.008917	0.04599
235712	Mrgpra2b	C2	0.368229	0.03703032	0.089259	-0.82527	0.00011	0.005683
16415	Itgb2l	C2	0.191327	0.47090215	0.447945	-0.8491	0.000138	0.005932
68891	Cd177	C2	0.154061	0.40361381	0.406932	-0.85022	0.000111	0.005691
242253	Dnai3	C2	-0.33904	0.07034044	0.134666	-0.86848	0.000126	0.005797
224840	Treml4	C2	0.125769	0.40731428	0.40937	-0.86848	0.000729	0.012013
546644	Ly6g	C2	0.445953	0.04822368	0.106052	-0.93441	1.88E-05	0.00323
13587	Ear2	C2	0.045487	0.8671266	0.582266	-0.95345	0.002028	0.020018
15439	Hp	C2	-0.04067	0.74052587	0.554481	-0.96255	0.005905	0.036454
16819	Lcn2	C2	0.544503	0.06304911	0.126405	-1.08496	0.000139	0.005925
17523	Mpo	C2	-0.58496	0.0520459	0.111388	-1.86372	3.25E-05	0.003973
67425	Eps8l1	C3	-0.84975	0.07174299	0.136337	1.247979	1.15E-05	0.002619
20716	Serpina3n	C3	-0.29248	0.28253773	0.328092	1.084963	0.003099	0.025139
105246823	Gm42047	C3	-0.33904	0.26572481	0.314364	1.055516	1.07E-06	0.00105
11815	Apod	C3	-0.1065	0.26045276	0.310988	1.030677	8.91E-05	0.005157
16790	Anpep	C3	-0.40368	0.00841313	0.032838	1.029447	4.96E-05	0.004452
57875	Angptl4	C3	-0.29248	0.11473801	0.186369	1.029447	0.001932	0.01933
382111	Susd5	C3	-0.48476	0.0285654	0.074815	0.984761	0.000265	0.007417
66977	Nuf2	C3	0	0.95373797	0.60336	0.953445	0.002352	0.02143
56149	Tamalin	C3	-0.576	0.03147413	0.07964	0.926221	9.49E-05	0.005303
16533	Kcnmb1	C3	-0.36848	0.03023397	0.077326	0.903677	0.0009	0.013441
53417	Hif3a	C3	-0.1007	0.16441469	0.234659	0.893183	7.56E-05	0.004803
66695	Aspn	C3	-0.41504	0.0194982	0.057953	0.868483	0.001037	0.014127
381310	Stum	C3	-0.41504	0.02600842	0.070183	0.868483	0.00183	0.018693
14613	Gja5	C3	-0.41504	0.04301668	0.097353	0.868483	0.00057	0.010556
21380	Tbx1	C3	-0.576	0.01948499	0.057951	0.868483	0.001636	0.01777
22240	Dpysl3	C3	-0.576	0.01753147	0.053872	0.868483	0.003596	0.027318
68659	Gask1b	C3	-0.576	0.01753147	0.053854	0.868483	0.000796	0.012627
16592	Fabp5	C3	-0.20752	0.36182364	0.382457	0.861233	0.001816	0.018605
18125	Nos1	C3	-0.45345	0.02668088	0.071415	0.85022	0.00146	0.016761
18823	Plp1	C3	-0.14005	0.47169294	0.448328	0.842631	0.008315	0.04407
229003	Helz2	C3	-0.36123	0.10785194	0.178695	0.830533	0.000218	0.006921
16011	Igfbp5	C3	-0.16974	0.28685859	0.330992	0.821025	0.001454	0.016755
54141	Spag5	C3	-0.30142	0.02487139	0.068027	0.801416	0.00305	0.024888
18132	Notch4	C3	-0.51601	0.01612392	0.050967	0.799384	0.000609	0.01077
20745	Spock1	C3	0	0.8194384	0.570348	0.792481	0.001536	0.017277
20681	Sox8	C3	-0.29248	0.12743983	0.199712	0.792481	0.002614	0.022875

68764	Cdhr3	C3	-0.29248	0.15521081	0.22616	0.792481	0.001662	0.017905
100702	Gbp6	C3	-0.40368	0.04248698	0.097271	0.792481	0.003425	0.026639
14586	Gfra2	C3	-0.5	0.0353654	0.086462	0.792481	0.00726	0.040565
99899	Ifi44	C3	-0.5	0.04322205	0.097579	0.792481	0.004226	0.030115
22390	Wee1	C3	-0.58496	0.08272803	0.150864	0.792481	0.007914	0.042873
622175	E430024I08Rik	C3	-0.58496	0.04634665	0.102828	0.792481	0.000991	0.013918
18810	Plec	C3	-0.44924	0.00374552	0.019043	0.773358	0.000463	0.009702
93695	Gpnmb	C3	0.020321	0.5486903	0.479883	0.77216	0.005347	0.034578
381409	Cdh26	C3	-0.34768	0.00601673	0.026289	0.769493	0.000335	0.008208
235503	Eef1a1-ps1	C3	0.055047	0.37884449	0.393706	0.766916	3.7E-06	0.001507
66395	Ahnak	C3	-0.41246	0.01566183	0.049933	0.763694	0.001417	0.016523
627235	Gm6741	C3	-0.40775	0.01640102	0.051562	0.762098	0.000456	0.009581
233744	Spon1	C3	-0.29248	0.04547958	0.101366	0.745927	0.001351	0.015987
320064	D130017N08Rik	C3	0	0.93373608	0.594852	0.736966	0.000828	0.01284
22418	Wnt5a	C3	-0.41504	0.09008117	0.158649	0.736966	0.00579	0.036035
22169	Cmpk2	C3	-0.33329	0.03961454	0.092877	0.716056	0.001119	0.014657
207607	Ccdc40	C3	0	0.82378807	0.571271	0.707519	0.000199	0.006721
100043823	Gm4673	C3	-0.20752	0.25860989	0.309626	0.707519	0.003735	0.027899
13482	Dpp4	C3	-0.41504	0.00160287	0.010952	0.707519	0.000413	0.009123
320563	Islr2	C3	-0.41504	0.00027453	0.003716	0.707519	0.005181	0.033955
93699	Pcdhgb1	C3	-0.5	0.02982998	0.076696	0.707519	0.000304	0.007805
100041621	Gm3435	C3	-0.5	0.03908361	0.092169	0.707519	0.000182	0.006394
14102	Fas	C3	-0.576	0.02168963	0.062268	0.707519	0.006426	0.038177
224697	Adamts10	C3	-0.34975	0.08000842	0.147591	0.693512	0.001288	0.015774
29818	Hspb7	C3	-0.33824	0.02330821	0.065137	0.685358	0.00018	0.006346
207728	Pde2a	C3	-0.27983	0.05060146	0.109538	0.682923	0.002246	0.021091
209837	Slc38a5	C3	-0.43536	0.03183305	0.080219	0.681285	0.003739	0.027902
12870	Cp	C3	-0.39143	0.01073516	0.038524	0.681285	0.000871	0.013174
26936	Mprip	C3	-0.31199	0.01151309	0.040224	0.671873	0.000292	0.007697
26949	Vat1	C3	-0.27627	0.02287216	0.064444	0.668935	6.73E-05	0.004702
240427	Setbp1	C3	-0.48543	0.02039898	0.059767	0.666712	0.007793	0.042431
103712	6330403K07Rik	C3	0	0.77065958	0.562931	0.660964	0.004055	0.029433
211712	Pcdh9	C3	-0.5	0.02982998	0.076717	0.660964	0.001116	0.014679
70536	Qpct	C3	0	0.93373608	0.59993	0.660964	0.000147	0.005995
11540	Adora2a	C3	-0.36848	0.00654913	0.027865	0.660964	0.002872	0.023956
18791	Plat	C3	-0.40698	0.05570567	0.116738	0.659599	0.006437	0.038216
208836	Fanci	C3	-0.02462	0.89593956	0.589137	0.65927	0.000194	0.006633
328329	Mast4	C3	-0.4287	0.00371836	0.018947	0.652427	0.000875	0.01319
327978	Slfn5	C3	-0.2218	0.1892012	0.256249	0.646811	0.000613	0.010787
72792	2810459M11Rik	C3	-0.40236	0.00129842	0.009518	0.634839	0.001031	0.014083
16500	Kcnb1	C3	-0.36848	0.01169044	0.04069	0.631517	0.002225	0.020956

109323	C1qtnf7	C3	-0.04206	0.34351901	0.370641	0.627027	0.000232	0.007039
13518	Dst	C3	-0.34426	0.15025409	0.221235	0.620751	0.006765	0.039117
57756	Fhl5	C3	-0.37314	0.0348692	0.085701	0.619712	0.001661	0.017918
13730	Emp1	C3	-0.18129	0.71144514	0.545861	0.61852	0.000853	0.013025
14164	Fgf1	C3	-0.411	0.00714586	0.029708	0.61852	0.000994	0.013927
214137	Arhgap29	C3	-0.43355	0.00518973	0.023787	0.615306	0.002173	0.020701
226040	Tmem252	C3	-0.40368	0.03790662	0.090453	0.611196	0.008117	0.043566
18208	Ntn1	C3	-0.40103	0.02936273	0.075958	0.603225	0.001798	0.018534
235041	Kank2	C3	-0.4303	0.00096235	0.007864	0.601642	0.000964	0.013776
268396	Sh3pxd2b	C3	-0.19351	0.06186807	0.124767	0.596323	0.000683	0.011498
278795	Lrrc10b	C3	-0.30124	0.02457548	0.067437	0.593718	0.000807	0.012739
209086	Samd9l	C3	-0.18926	0.25348369	0.306021	0.590286	0.001652	0.017838
50708	H1f2	C3	-0.44681	0.03369073	0.083559	0.588011	0.001882	0.019027
106585	Ankrd12	C3	-0.43801	0.01867095	0.056363	0.587786	0.000534	0.01026
100039796	Tgtp2	C3	-0.29248	0.06836944	0.132787	0.584963	0.00641	0.038149
11838	Arc	C3	0	0.54740959	0.48026	0.584963	0.000962	0.013775
12021	Bard1	C3	0	0.77422213	0.563001	0.584963	0.000485	0.009807
74918	Iqca	C3	0	0.7205015	0.549667	0.584963	0.00073	0.012009
66634	Mcm8	C3	0	0.54523771	0.481044	0.584963	0.000485	0.009787
72535	Aldh1b1	C3	0	0.93373608	0.598559	0.584963	0.006673	0.038841
67080	1700019D03Rik	C3	0	0.54740959	0.479942	0.584963	0.001548	0.017306
242894	Actr3b	C3	0	0.8194384	0.570305	0.584963	0.008624	0.045024
56747	Sez6l	C3	0	0.4112744	0.412098	0.584963	0.001022	0.014025
12879	Cys1	C3	0	0.91373231	0.594587	0.584963	0.005977	0.03676
100040540	Gm10288	C3	0	0.53435172	0.478693	0.584963	0.009789	0.048469
619292	G430095P16Rik	C3	0	0.93373608	0.595836	0.584963	0.006673	0.038772
100039495	Gm15706	C3	0	0.77422213	0.561458	0.584963	0.000207	0.00683
100043761	Gm14399	C3	0	0.93373608	0.595016	0.584963	0.006673	0.038749
16948	Lox	C3	-0.16096	0.59372374	0.499454	0.584963	0.004564	0.031337
194590	Reps2	C3	-0.16096	0.52540774	0.476863	0.584963	0.00085	0.013017
140493	Kcnn3	C3	-0.29248	0.03670628	0.088617	0.584963	0.001143	0.014771
57441	Gmnn	C3	-0.29248	0.24734781	0.304045	0.584963	0.000471	0.009804
20557	Slfn3	C3	-0.29248	0.36090045	0.382005	0.584963	0.000241	0.00714
19183	Psmc3ip	C3	-0.29248	0.24734781	0.303924	0.584963	0.000207	0.006877
71137	Rfx4	C3	-0.29248	0.06189558	0.124795	0.584963	0.002943	0.024364
22712	Zfp54	C3	-0.29248	0.25164887	0.306121	0.584963	0.009789	0.048567
16580	Kifc5b	C3	-0.29248	0.04271636	0.097435	0.584963	2.83E-06	0.001457
71233	Enkur	C3	-0.29248	0.17781019	0.246445	0.584963	0.006673	0.038864
67182	Pdzk1ip1	C3	-0.29248	0.03306313	0.082533	0.584963	0.000133	0.005882
56087	Dnah10	C3	-0.29248	0.25164887	0.30592	0.584963	0.006673	0.038818
56175	Bace2	C3	-0.29248	0.11107389	0.182239	0.584963	0.000485	0.009767
17444	Grap2	C3	-0.29248	0.25164887	0.30588	0.584963	0.009789	0.048542

210356	Nckap5	C3	-0.29248	0.24734781	0.30312	0.584963	0.006673	0.038795
14723	Gp1ba	C3	-0.29248	0.25164887	0.30576	0.584963	0.009789	0.048518
215772	Adgb	C3	-0.29248	0.25164887	0.305639	0.584963	0.009789	0.048493
100504389	9030407P20Rik	C3	-0.29248	0.24734781	0.302799	0.584963	0.009789	0.048395
74186	Ccdc3	C3	-0.32193	0.07281869	0.137759	0.584963	0.005177	0.03395
93711	Pcdhga3	C3	-0.58496	0.08272803	0.150834	0.584963	0.005309	0.034448
170736	Parvb	C3	-0.26564	0.07104487	0.135483	0.582933	0.000821	0.012785
72834	Cerox1	C3	0	0.54523771	0.479536	0.576002	0.001176	0.015079
14412	Slc6a13	C3	-0.20752	0.09901069	0.168726	0.576002	0.003267	0.025963
195434	Utp14b	C3	-0.20752	0.24114592	0.299523	0.576002	0.005566	0.03541
638247	9530082P21Rik	C3	-0.20752	0.05116825	0.110352	0.576002	0.000796	0.012645
224836	Usp49	C3	-0.29248	0.19029876	0.257096	0.576002	0.002807	0.023574
11899	Astn1	C3	-0.36848	0.03859299	0.091478	0.576002	0.003085	0.02507
22223	Uchl1	C3	-0.41504	0.09722801	0.166794	0.576002	0.005804	0.036054
320560	Dennd5b	C3	-0.41504	0.00122404	0.009191	0.576002	0.001521	0.017248
17064	Cd93	C3	-0.3332	0.03626255	0.087889	0.574546	0.003342	0.026288
101497	Plekhg2	C3	-0.38842	0.0181839	0.055307	0.570229	0.001174	0.0151
74182	Gpcpd1	C3	-0.31935	0.03643505	0.088146	0.568344	0.000142	0.005872
19411	Rarg	C3	-0.44553	0.00279484	0.015749	0.564766	0.005744	0.035956
117160	Ttyh2	C3	-0.34757	0.00404679	0.019951	0.564052	0.001018	0.013987
71653	Shtn1	C3	-0.12256	0.31601263	0.351955	0.559791	0.001438	0.016729
140810	Ttbk2	C3	-0.40971	0.01837091	0.055711	0.557086	0.007213	0.040372
26390	Mapkbp1	C3	-0.39425	0.02108665	0.060933	0.556729	0.007045	0.040006
17311	Kitl	C3	-0.38173	0.01242119	0.042672	0.553803	0.000905	0.013431
18628	Per3	C3	-0.35022	0.07940133	0.14688	0.549768	0.0021	0.020443
243219	2900026A02Rik	C3	-0.39571	0.00233727	0.013943	0.548842	0.000782	0.012523
69938	Scrn1	C3	-0.24271	0.12361126	0.195399	0.546555	0.004794	0.032258
108100	Baiap2	C3	-0.34423	0.0131414	0.044486	0.540568	9.41E-05	0.005293
27015	Polk	C3	-0.41034	0.00538183	0.024401	0.536532	0.001102	0.014589
70097	Sash1	C3	-0.35448	0.00585182	0.025787	0.536075	0.001558	0.017402
71660	Rarres2	C3	-0.1112	0.24571429	0.30272	0.535195	0.003494	0.026831
233064	Wdr62	C3	-0.33904	0.08406366	0.151775	0.535195	0.008652	0.045126
16773	Lama2	C3	-0.35391	0.06776024	0.132075	0.535195	0.005056	0.033427
74257	Tspan17	C3	-0.424	0.00114482	0.008825	0.535195	0.000235	0.007057
229731	Slc25a24	C3	-0.45023	0.01732804	0.053513	0.535195	0.002251	0.021117
100382	AW011738	C3	-0.45023	0.03328083	0.082786	0.535195	0.001735	0.018196
13609	S1pr1	C3	-0.42833	0.00642376	0.027585	0.534532	0.002305	0.021414
75007	Mindy1	C3	-0.40642	0.00495276	0.02302	0.528292	0.002078	0.020315
20740	Sptan1	C3	-0.36374	0.00250085	0.014618	0.527964	0.000918	0.013522
20719	Serpinb6a	C3	-0.22522	0.10841171	0.179269	0.524952	0.001086	0.014519
16513	Kcnj10	C3	-0.2839	0.06469845	0.128488	0.522788	0.003674	0.02757
227937	Pkp4	C3	-0.26684	0.03226739	0.081115	0.520321	0.002177	0.020697

29876	Clic4	C3	-0.08759	0.63108127	0.515198	0.520305	0.004238	0.030092
67533	Ppfibp1	C3	-0.43125	0.00323789	0.017379	0.519543	0.00348	0.02681
22782	Slc30a1	C3	-0.39313	0.00389061	0.019534	0.518721	0.000517	0.010091
11987	Slc7a1	C3	-0.2033	0.1282688	0.20057	0.515513	0.010099	0.04948
26570	Slc7a11	C3	-0.22075	0.18683314	0.254194	0.513236	0.003765	0.028038
223254	Farp1	C3	-0.34757	0.00645687	0.027664	0.512768	0.002213	0.020901
71228	Dlg5	C3	-0.43857	0.01138516	0.039928	0.507323	0.006347	0.037981
19663	Rbpms	C3	-0.36668	0.0115958	0.040406	0.5	0.001787	0.018483
20449	St8sia1	C3	0	0.93373608	0.599181	0.5	0.001327	0.01585
235180	Fez1	C3	-0.13152	0.22400968	0.28595	0.5	0.004355	0.03046
237436	Gas2l3	C3	-0.20752	0.67355724	0.531684	0.5	0.00693	0.039742
667370	Ifit3b	C3	-0.24271	0.18310957	0.250861	0.5	0.00846	0.04455
11733	Ank1	C3	-0.29248	0.26643751	0.315046	0.5	0.009292	0.047204
224833	AI661453	C3	-0.29248	0.05947495	0.12196	0.5	0.009934	0.048938
14219	Ccn2	C3	-0.40368	0.0040776	0.020028	0.5	0.003479	0.026827
16590	Kit	C3	-0.41504	0.01943426	0.057893	0.5	0.010109	0.049428
11606	Agt	C3	-0.41504	0.00816085	0.032414	0.5	0.006814	0.039335
58237	Nkain4	C3	-0.5	0.02870381	0.075051	0.5	0.005455	0.03493
237360	Adamts14	C3	-0.5	0.05904335	0.121344	0.5	0.010162	0.049564
12724	Clcn2	C3	-0.33904	0.06339293	0.12682	0.5	0.008539	0.04482
227059	Slc39a10	C3	-0.35534	0.01425638	0.046989	0.496958	0.001872	0.018956
101351	Eogt	C3	-0.44737	0.00825141	0.032454	0.490446	0.008399	0.044373
353187	Nr1d2	C3	-0.35733	0.02142281	0.06177	0.489313	0.000315	0.00791
70122	Mllt3	C3	-0.26625	0.066431	0.130945	0.487002	0.003433	0.026658
22700	Zfp40	C3	-0.32604	0.11641922	0.187814	0.487002	0.000984	0.013876
319504	Nrcam	C3	-0.33904	0.02472088	0.067796	0.485427	0.009218	0.047
94092	Trim16	C3	-0.37423	0.0373966	0.089722	0.485427	0.010197	0.049638
105445	Dock9	C3	-0.34197	0.00238327	0.014145	0.485267	0.002136	0.02051
100142658	Gm10222	C3	-0.23875	0.06780869	0.132059	0.481263	0.006981	0.039899
21366	Slc6a6	C3	-0.27525	0.02044742	0.059833	0.476357	4.24E-05	0.004368
29815	Bcar3	C3	-0.41001	0.00345895	0.018042	0.472776	0.003457	0.02674
20364	Selenow	C3	-0.13565	0.33539758	0.365327	0.472246	0.009562	0.047978
226519	Lamc1	C3	-0.37692	0.00294396	0.016351	0.4693	0.003044	0.024859
16440	Itpr3	C3	-0.21497	0.07692504	0.143359	0.467654	0.002116	0.020479
57782	Rbak	C3	-0.44654	0.01752487	0.053869	0.464805	0.001797	0.018563
216033	Ctnna3	C3	-0.17182	0.04977338	0.108251	0.464297	0.008311	0.044075
21417	Zeb1	C3	-0.27467	0.02706584	0.072008	0.463925	0.00137	0.016147
16324	Inhbb	C3	-0.21943	0.02639954	0.070949	0.463033	0.002545	0.022495
231287	Atp10d	C3	-0.28171	0.06234945	0.125437	0.463	0.004609	0.031536
218215	Rnf144b	C3	-0.25619	0.03765555	0.090156	0.462888	0.000192	0.006648
94246	Arid4b	C3	-0.30753	0.04927909	0.107479	0.4627	0.002683	0.023148
50767	Pnpla6	C3	-0.38413	0.00557401	0.024943	0.461774	0.004576	0.031395

16184	Il2ra	C3	-0.16904	0.03244457	0.081494	0.461521	0.001012	0.013983
72446	Prr5l	C3	-0.05346	0.64107932	0.519376	0.460524	0.001232	0.015356
20893	Bhlhe40	C3	-0.13782	0.21566277	0.279573	0.460197	0.001087	0.014509
224139	Golgb1	C3	-0.44765	0.00867513	0.033494	0.459193	0.0064	0.038135
12334	Capn2	C3	-0.397	0.00073144	0.006613	0.456026	0.00402	0.02929
56788	Scube2	C3	-0.41877	0.01233772	0.042448	0.455034	0.009097	0.046525
226751	Cdc42bpa	C3	-0.38729	0.00114405	0.008827	0.453928	0.00651	0.038462
347722	Agap1	C3	-0.35712	0.00039977	0.004598	0.453445	0.004128	0.029828
320528	Vps13c	C3	-0.37495	0.24558446	0.302722	0.453012	0.007396	0.040997
73182	Pear1	C3	-0.3283	0.03425086	0.084563	0.45226	0.003472	0.026813
50887	Hmgn5	C3	-0.24271	0.23436043	0.293895	0.450232	0.010087	0.049445
239122	Setdb2	C3	-0.35391	0.0216897	0.062249	0.450232	0.003596	0.027336
235283	Gramd1b	C3	-0.41253	0.00329404	0.017568	0.450232	0.001637	0.017738
238447	Igha	C4	-2.32433	3.0021E-06	0.000479	2.160964	1.42E-07	0.001391
16071	Igkc	C4	-2.83621	7.7741E-07	0.000379	1.792481	9.42E-06	0.002363
213393	Depp1	C4	-1.08496	0.00031898	0.004057	1.753897	1.02E-06	0.001114
17880	Myh11	C4	-0.93165	0.00028997	0.003797	1.626895	1.19E-06	0.001057
19416	Rasd1	C4	-1	9.9348E-05	0.002233	1.522197	4.48E-07	0.000731
19713	Ret	C4	-0.85391	0.0046644	0.022124	1.505986	0.000112	0.005667
19736	Rgs4	C4	-0.9228	0.00026167	0.003682	1.505757	1.36E-05	0.002707
116621581	Derpc	C4	-1.03869	0.00820754	0.032502	1.489724	0.001202	0.015252
319508	Syt15	C4	-1.13152	7.6845E-05	0.001923	1.437235	5.05E-06	0.001648
14281	Fos	C4	-1.84904	8.4096E-08	0.000389	1.419883	3.79E-07	0.000927
72289	Malat1	C4	-0.82053	0.00752711	0.030606	1.398414	3.54E-05	0.004119
107589	Mylk	C4	-0.79248	0.00107351	0.008423	1.380358	1.42E-05	0.002726
16069	Jchain	C4	-1.83232	3.7607E-07	0.000435	1.332322	1.58E-07	0.000774
16007	Ccn1	C4	-1.23977	1.7163E-06	0.000441	1.330365	4.85E-06	0.001758
14013	Mecom	C4	-1.20027	3.5218E-06	0.000487	1.320773	1.62E-06	0.001134
72630	Hspa12b	C4	-0.82	0.00031248	0.003991	1.300452	9.28E-06	0.00239
72333	Palld	C4	-0.68129	0.00266893	0.015206	1.292481	2.31E-05	0.003524
213469	Lgi3	C4	-1.16096	0.00060619	0.005846	1.292481	4E-05	0.00435
239691	AU021092	C4	-1.22239	2.0609E-05	0.001015	1.292481	2.39E-05	0.003597
320106	Slc38a11	C4	-0.95023	0.00016742	0.002941	1.292481	5.74E-05	0.004566
93960	Nkd1	C4	-0.94654	7.3654E-05	0.001905	1.257287	7.01E-06	0.002018
22004	Tpm2	C4	-0.87381	0.00061183	0.005876	1.25595	2.26E-05	0.003514
54611	Pde3a	C4	-0.70752	0.00158914	0.010915	1.25125	3.27E-05	0.003897
13717	Eln	C4	-0.7978	0.00013279	0.002621	1.242713	2.64E-05	0.003805
442801	Arhgef15	C4	-0.93857	0.0001091	0.002333	1.231053	1.69E-05	0.003064
11754	Aoc3	C4	-0.91825	0.00077043	0.006859	1.229716	0.000117	0.005649
18821	Pln	C4	-0.924	0.00017397	0.002988	1.229716	8.18E-05	0.004972
239559	A4galt	C4	-0.90368	0.00010408	0.002305	1.229716	2.68E-05	0.003807
234395	Ushbp1	C4	-1.16096	4.1729E-05	0.00139	1.229716	1.76E-05	0.003076

54485	Dll4	C4	-1.07806	0.00044049	0.004832	1.22784	0.000119	0.005685
16449	Jag1	C4	-0.73328	0.00029879	0.003874	1.223729	2.67E-06	0.001632
98932	Myl9	C4	-0.90501	0.00033525	0.004178	1.223729	3.26E-05	0.003941
70059	Degs2	C4	-1.16096	2.892E-06	0.000478	1.21648	1.17E-05	0.002604
93689	Lmod1	C4	-0.63152	0.01063763	0.038278	1.21648	0.000199	0.006699
18131	Notch3	C4	-0.87645	0.00011723	0.002423	1.212511	1.48E-05	0.002781
15896	Icam2	C4	-0.84225	0.00054322	0.005385	1.210732	3.1E-05	0.004149
11475	Acta2	C4	-0.79857	0.00096241	0.007858	1.202119	5.02E-05	0.004426
16009	Igfbp3	C4	-0.66671	0.00236681	0.014074	1.200269	3.16E-05	0.004063
13876	Erg	C4	-1	5.2541E-05	0.00159	1.181285	3.13E-05	0.004133
668303	Kif26a	C4	-1.04286	5.0323E-05	0.001548	1.181285	2.81E-05	0.003929
19252	Dusp1	C4	-1.53626	2.388E-07	0.000368	1.164151	2.83E-06	0.001538
12323	Camk2b	C4	-1.17525	0.00030523	0.00393	1.160964	2.24E-06	0.001463
231440	Parm1	C4	-0.81871	0.0005329	0.005369	1.160964	0.00011	0.005682
242553	Kank4	C4	-0.90368	0.00014128	0.002703	1.160964	6.59E-05	0.00467
75689	Higd1b	C4	-1	2.8276E-06	0.000476	1.160964	1.18E-05	0.002513
73181	Nfatc4	C4	-1	0.00010043	0.002251	1.160964	0.000377	0.008644
16981	Lrrn3	C4	-1.16096	1.4585E-06	0.000397	1.160964	1.18E-05	0.002565
81701	Egfl8	C4	-0.83904	0.00015233	0.002804	1.153714	7.65E-06	0.002138
56349	Net1	C4	-0.96174	6.8102E-05	0.001844	1.147728	1.53E-05	0.002817
16598	Klf2	C4	-1.53657	1.5759E-06	0.000417	1.143914	0.000158	0.006295
68888	Gkn3	C4	-1.22478	3.38E-05	0.001247	1.131517	0.000219	0.006875
380928	Lmo7	C4	-1.05774	3.9631E-05	0.001359	1.12649	5.63E-05	0.004631
225341	Lims2	C4	-0.86158	0.00059973	0.005808	1.123361	0.00022	0.006878
17540	Irag1	C4	-0.70752	0.00147622	0.010394	1.119733	0.000137	0.005944
21345	Tagln	C4	-0.88277	0.00025094	0.003608	1.11633	6.95E-05	0.004722
16842	Lef1	C4	-1.05774	9.813E-06	0.000704	1.115477	2.82E-05	0.003892
19737	Rgs5	C4	-0.59527	0.00118673	0.009006	1.115355	5E-05	0.004445
84004	Mcam	C4	-0.67682	0.00075932	0.006792	1.115306	4.2E-05	0.004416
13616	Edn3	C4	-0.90689	6.4382E-05	0.00179	1.114409	3.93E-05	0.004322
76854	Gper1	C4	-0.88378	0.00042936	0.004749	1.111196	6.08E-05	0.004612
238803	Zfp366	C4	-0.67807	0.00198409	0.012488	1.111196	5.74E-05	0.004601
100038755	Gm9917	C4	-0.81871	0.00044887	0.004889	1.111196	0.000134	0.005893
217480	Dgkb	C4	-0.86848	0.00021036	0.003241	1.111196	0.000584	0.010641
17470	Cd200	C4	-0.99497	2.2197E-05	0.001028	1.111196	3.91E-05	0.004351
224796	Clic5	C4	-0.9323	5.574E-05	0.001649	1.092933	4.81E-05	0.004438
232237	Fgd5	C4	-0.65995	0.00193762	0.012304	1.089989	6.25E-05	0.0046
27205	Podxl	C4	-0.75071	0.00078397	0.006926	1.088493	9.15E-05	0.005207
12741	Cldn5	C4	-0.96047	9.5612E-05	0.002191	1.086004	6.29E-05	0.004558
71774	Shroom1	C4	-0.87744	0.00045435	0.004926	1.084963	0.000307	0.007813
13614	Edn1	C4	-0.8888	9.6738E-05	0.002195	1.084963	3.36E-06	0.001428
227545	Proser2	C4	-1	3.5608E-05	0.001278	1.084963	0.000129	0.005843

217166	Nr1d1	C4	-0.81339	0.00010641	0.002318	1.083192	4.16E-07	0.000813
65962	Slc9a3r2	C4	-0.61431	0.00320249	0.017249	1.077775	5.64E-05	0.004595
240185	Jcad	C4	-0.69523	0.00080706	0.007096	1.076575	5.91E-05	0.004555
259097	Olfr558	C4	-0.73697	0.00343948	0.018012	1.076002	0.000444	0.009465
226250	Afap1l2	C4	-0.75407	0.00025325	0.00363	1.074196	2.96E-05	0.004016
13819	Epas1	C4	-0.74021	0.00044505	0.004865	1.073293	5.92E-05	0.004525
14612	Gja4	C4	-0.79248	0.00018229	0.003069	1.068752	5.8E-05	0.004539
101772	Ano1	C4	-0.89479	0.00018031	0.003046	1.068752	0.000115	0.005659
80721	Slc19a3	C4	-0.90368	6.7441E-05	0.001836	1.068752	4.4E-05	0.004216
15220	Foxq1	C4	-1.20027	2.208E-06	0.000454	1.068752	7.8E-05	0.004832
18127	Nos3	C4	-0.69927	0.00208431	0.012934	1.066316	0.000104	0.00558
20668	Sox13	C4	-0.75729	0.00049985	0.005136	1.065979	6.8E-05	0.004688
13601	Ecm1	C4	-0.52945	0.00412968	0.020241	1.061198	2.17E-05	0.003426
78754	Galnt15	C4	-0.70027	0.00132766	0.009679	1.055516	4.9E-05	0.004435
72565	Uaca	C4	-0.7506	0.00018839	0.00312	1.053458	2.55E-05	0.003727
14254	Flt1	C4	-0.77044	0.00041588	0.004707	1.049417	8.5E-05	0.005043
23928	Lamc3	C4	-0.6112	0.00092064	0.007707	1.043731	5.37E-05	0.004608
16404	Itga7	C4	-0.90368	7.2661E-05	0.001922	1.043731	0.00012	0.005679
67866	Wfdc1	C4	-0.72905	0.00236319	0.014061	1.042444	0.000337	0.008199
619288	Fam71a	C4	-1.65243	2.0607E-07	0.000477	1.041231	0.001588	0.017513
64009	Syne1	C4	-1.23356	1.9301E-05	0.000976	1.038722	0.002577	0.022649
494504	Apcdd1	C4	-0.84925	2.8859E-05	0.001152	1.038502	6.8E-06	0.002015
16600	Klf4	C4	-1.31454	2.6369E-05	0.001095	1.033284	0.000497	0.009893
12351	Car4	C4	-1.00725	5.3241E-06	0.000567	1.032065	7.63E-05	0.004819
16776	Lama5	C4	-0.67807	0.00019399	0.003168	1.029751	4.05E-05	0.004356
623230	Tmem200b	C4	-0.66096	0.01107065	0.039331	1.029447	0.000355	0.008328
243653	Clec1a	C4	-0.86848	0.00047903	0.005092	1.029447	7.43E-05	0.004845
22419	Wnt5b	C4	-1	0.00015782	0.002848	1.029447	0.000324	0.008096
12288	Cacna1c	C4	-1	0.00014579	0.00276	1.029447	0.000355	0.008348
59091	Jph2	C4	-1.02945	0.00038203	0.004494	1.029447	0.002472	0.022084
20361	Sema7a	C4	-0.80559	0.00049115	0.005086	1.028515	9.54E-05	0.005306
12826	Col4a1	C4	-0.52479	0.00779926	0.031409	1.025353	0.000164	0.006327
259300	Ehd2	C4	-0.63877	0.0038923	0.019532	1.022418	0.000141	0.005904
60440	Iigp1	C4	-0.58496	0.00274885	0.015566	1.022197	0.000165	0.006316
78560	Adgra2	C4	-0.66828	0.00187006	0.012091	1.02091	0.000121	0.005697
22371	Vwf	C4	-0.83526	0.00060018	0.005806	1.020179	0.000246	0.007129
72022	Slc35f2	C4	-0.66096	0.00161204	0.01099	1.014874	0.000242	0.007113
104110	Adcy4	C4	-0.75892	0.00217671	0.013232	1.010174	0.000478	0.009849
241327	Olfml2a	C4	-0.65153	0.00093568	0.007728	1.009308	6.96E-05	0.004696
13170	Dbp	C4	-0.95345	7.1115E-05	0.001892	1.008961	2.76E-06	0.001587
21804	Tgfb1i1	C4	-0.71648	0.00187752	0.01208	1.005986	0.00023	0.007046
13346	Des	C4	-0.93723	0.00010629	0.002321	1.005986	0.000405	0.009021

57764	Ntn4	C4	-0.71425	0.00062675	0.005958	1.004494	0.000214	0.006894
381290	Atp2b4	C4	-0.69616	0.00029565	0.00385	1.003213	0.000133	0.005882
12827	Col4a2	C4	-0.55447	0.00736126	0.03017	1.002823	0.0002	0.006688
70574	Cpm	C4	-0.576	0.00398213	0.019811	1	0.000553	0.010364
11600	Angpt1	C4	-0.58496	0.00837256	0.032763	1	0.001325	0.015854
16497	Kcnab1	C4	-0.58496	0.00926837	0.035025	1	0.000139	0.005957
13610	S1pr3	C4	-0.63152	0.00175624	0.011598	1	9.8E-05	0.005417
228788	Ccm2l	C4	-0.71648	0.0018458	0.011976	1	0.000507	0.010023
83691	Crispld1	C4	-0.73697	0.00060914	0.005869	1	0.000482	0.009817
20713	Serpini1	C4	-0.73697	0.00060914	0.005863	1	0.000553	0.010345
269831	Tspan12	C4	-0.73697	0.00027078	0.003709	1	0.000634	0.011014
109042	Cavin3	C4	-0.76303	0.00017832	0.003029	1	0.000119	0.005698
11639	Ak4	C4	-0.79248	7.3636E-05	0.001931	1	4.15E-05	0.004413
75905	Dipk2b	C4	-0.79248	0.0016448	0.011156	1	0.000311	0.007882
18377	Omg	C4	-0.79248	0.00180908	0.011829	1	0.000253	0.007204
73173	Pcdh18	C4	-0.86848	0.00023319	0.003422	1	0.001315	0.01588
277744	Srarp	C4	-0.86848	0.00089996	0.007609	1	0.002493	0.022197
623474	Rad54b	C4	-0.86848	0.00320006	0.017256	1	0.000404	0.009026
108897	Aif1l	C4	-0.8888	9.6901E-06	0.000701	1	3.67E-05	0.004175
16431	Itm2a	C4	-0.93303	8.0651E-06	0.000667	0.995468	5.68E-05	0.004595
218877	Sema3g	C4	-0.74781	0.00374913	0.019051	0.994676	0.000318	0.007988
12006	Axin2	C4	-1.06875	7.813E-07	0.000362	0.99275	0.000123	0.005741
74144	Robo4	C4	-0.9323	0.000121	0.002468	0.992447	0.000238	0.007124
20671	Sox17	C4	-0.85772	0.00026797	0.003714	0.991039	0.000115	0.005649
20257	Stmn2	C4	-0.90368	0.00022472	0.00334	0.98864	0.000179	0.006352
107449	Unc5b	C4	-1.19265	3.3989E-06	0.000492	0.985126	0.000132	0.005911
105450	Mmrn2	C4	-0.7782	0.00086711	0.007433	0.984994	0.000342	0.008233
22436	Xdh	C4	-0.65658	0.01167057	0.040636	0.984534	0.000139	0.005938
109624	Cald1	C4	-0.51446	0.00120695	0.0091	0.98396	0.000121	0.005692
13591	Ebf1	C4	-0.86123	0.00015738	0.002852	0.981737	0.000248	0.007169
54139	Irf6	C4	-0.576	0.00010618	0.002324	0.979679	4.97E-06	0.001735
18166	Npy1r	C4	-0.576	0.00396639	0.019754	0.979679	0.000508	0.010006
224405	Cyyr1	C4	-0.82604	0.00010825	0.002325	0.973766	0.000172	0.006418
278279	Tmtc2	C4	-0.924	8.3146E-05	0.00201	0.972429	0.000126	0.005771
20348	Sema3c	C4	-0.78558	5.4372E-06	0.000566	0.970195	4.77E-05	0.004444
243616	Slc6a11	C4	-1.054	0.00021126	0.003238	0.967452	0.001103	0.014582
433323	Sgpp2	C4	-0.85525	2.7287E-05	0.001103	0.966443	6.82E-05	0.004668
69903	Rasip1	C4	-0.72373	0.00095331	0.007839	0.963	0.000271	0.007501
246228	Vwa1	C4	-0.7547	0.00037463	0.004435	0.961689	0.000158	0.006283
170757	Adgrl4	C4	-0.62633	0.00250927	0.014648	0.961069	0.000273	0.007536
66873	Tril	C4	-1.2187	3.8964E-06	0.000494	0.958769	0.000168	0.006338
19285	Cavin1	C4	-0.69845	0.00066327	0.006209	0.95386	0.00021	0.006845

19038	Ppic	C4	-0.79248	0.00090627	0.007656	0.953445	0.000302	0.007841
16493	Kcna5	C4	-0.79248	0.00085134	0.007332	0.953445	0.002237	0.021022
231162	Cytl1	C4	-0.79248	7.3636E-05	0.001915	0.953445	6.18E-05	0.00458
57814	Kcne4	C4	-1	0.00012584	0.002533	0.953445	0.002401	0.021629
243547	Grip2	C4	-0.66096	0.00915672	0.034688	0.953445	0.005239	0.034243
69524	Esam	C4	-0.90742	0.00019535	0.003179	0.953445	0.000346	0.008292
17067	Ly6c1	C4	-0.77111	0.00076474	0.006821	0.94839	0.000363	0.008439
50778	Rgs1	C4	-0.64124	0.0010891	0.008495	0.947733	0.000105	0.005567
208431	Shroom4	C4	-0.74593	0.00026839	0.003714	0.947409	0.000211	0.00686
104027	Synpo	C4	-0.51457	0.00359723	0.018544	0.946542	0.001306	0.01585
20315	Cxcl12	C4	-1.04534	5.0223E-06	0.000554	0.944025	0.000173	0.006437
14461	Gata2	C4	-0.77028	0.00136713	0.009889	0.940209	0.000363	0.008427
22695	Zfp36	C4	-1.22707	3.649E-05	0.001294	0.939865	0.000411	0.009099
94214	Spock2	C4	-1.06884	6.5779E-06	0.000597	0.938939	0.000141	0.005928
108075	Ltbp4	C4	-0.91272	0.00020042	0.003216	0.935413	0.000371	0.008538
329251	Ppp1r12b	C4	-0.55774	0.00964387	0.036018	0.933249	0.000172	0.006438
54409	Ramp2	C4	-0.64609	0.00065175	0.006145	0.931524	0.000183	0.006414
110454	Ly6a	C4	-0.63962	0.00417691	0.020397	0.927271	0.000343	0.008238
67784	Plxnd1	C4	-0.79248	0.00037894	0.004464	0.927212	6.41E-05	0.004609
668212	Efr3b	C4	-0.84915	4.2672E-05	0.001411	0.925154	0.000166	0.00631
19263	Ptprb	C4	-0.7511	0.00036789	0.004412	0.925144	0.000206	0.006856
18452	P4ha2	C4	-0.53519	0.00305813	0.016783	0.923998	0.000234	0.007057
268977	Ltbp1	C4	-0.63152	0.00179938	0.011799	0.923998	0.001221	0.015351
20618	Sncg	C4	-0.74271	0.00091784	0.00769	0.923998	0.000667	0.011345
213783	Plekhg1	C4	-0.74271	1.1033E-05	0.000768	0.923998	0.000763	0.012334
21386	Tbx3	C4	-0.82768	0.00022435	0.003345	0.923998	0.000285	0.00755
319197	Gpr4	C4	-0.86848	9.6826E-05	0.002192	0.923998	0.000934	0.013626
70784	Rasl12	C4	-0.74271	0.00187667	0.012083	0.923998	0.000558	0.010415
80981	Arl4d	C4	-0.6358	0.00149418	0.010472	0.923998	0.000163	0.006335
19126	Prom1	C4	-0.97243	8.2961E-06	0.000662	0.923998	0.000137	0.005959
18671	Abcb1a	C4	-0.79579	0.00011881	0.002434	0.922221	0.000192	0.006631
224792	Adgrf5	C4	-0.79712	0.0001771	0.003014	0.921754	0.000226	0.006982
12562	Cdh5	C4	-0.6455	0.00129364	0.009498	0.919124	0.000242	0.007132
59308	Emcn	C4	-1.00428	3.8542E-06	0.000496	0.918251	0.000256	0.00726
140721	Caskin2	C4	-0.67198	0.00053195	0.005365	0.918251	0.000132	0.005886
15957	Ifit1	C4	-0.5	0.0021982	0.013337	0.915037	0.000157	0.006299
65964	Map3k20	C4	-0.66096	0.00060047	0.005803	0.915037	0.000361	0.00845
226777	C130074G19Rik	C4	-0.89945	1.343E-05	0.000813	0.910873	0.000112	0.005691
17153	Mal	C4	-0.81871	0.00026398	0.003703	0.908943	0.000197	0.006706
67801	Pllp	C4	-1.01118	1.1843E-05	0.000783	0.907958	0.000161	0.006279
11982	Atp10a	C4	-0.63116	0.00184457	0.011976	0.904903	0.000274	0.007496
58226	Cacna1h	C4	-0.66096	0.00166979	0.011268	0.903677	0.0009	0.013421

19279	Ptprr	C4	-0.79248	0.00033162	0.004166	0.903677	0.000593	0.010703
13617	Ednra	C4	-0.79248	0.00038646	0.004523	0.903677	0.000699	0.011673
214239	Ccdc9b	C4	-0.79248	7.5627E-05	0.001929	0.903677	0.001322	0.015874
20680	Sox7	C4	-0.80735	0.00020837	0.003242	0.903677	0.000974	0.013852
21414	Tcf7	C4	-0.90368	0.0001055	0.002326	0.903677	0.000352	0.008371
67252	Cap2	C4	-0.95345	0.00015679	0.002846	0.903677	0.00075	0.012223
14368	Fzd6	C4	-0.93723	1.2194E-06	0.000403	0.903677	0.000117	0.005694
18008	Nes	C4	-0.56437	0.002014	0.012591	0.899851	0.000161	0.006293
58234	Shank3	C4	-0.69616	0.00119861	0.009081	0.89068	0.000863	0.013132
241576	Ldlrad3	C4	-0.85022	0.00016615	0.002941	0.888804	0.000251	0.007217
214547	She	C4	-0.79248	0.00058158	0.00568	0.884694	0.000354	0.00835
80880	Kank3	C4	-0.69779	0.0011686	0.008934	0.883777	0.000256	0.00722
434215	Lrrc32	C4	-0.74271	0.00431787	0.020887	0.882767	0.002763	0.023383
14115	Fbln2	C4	-0.73516	0.00043205	0.004768	0.877444	0.000281	0.007559
329416	Nostrin	C4	-0.79248	0.00030192	0.003904	0.877444	0.000794	0.012646
22341	Vegfc	C4	-0.90368	0.00028709	0.003786	0.877444	0.000848	0.01303
226041	Pgm5	C4	-0.90368	0.00010546	0.00233	0.877444	0.000242	0.00715
66333	Aqp11	C4	-1.08496	3.1981E-06	0.000478	0.877444	0.000794	0.012625
20510	Slc1a1	C4	-1.24593	5.5956E-06	0.000557	0.877444	0.000898	0.013432
21687	Tek	C4	-0.62897	0.00184341	0.011977	0.875033	0.00027	0.007536
320183	Msrb3	C4	-0.63743	0.0007117	0.006498	0.873806	0.000929	0.013635
94242	Tinagl1	C4	-0.58541	0.00174659	0.011551	0.873122	0.000527	0.010195
16476	Jun	C4	-1.19991	7.7317E-06	0.000657	0.870946	0.000283	0.007536
56229	Thsd1	C4	-0.77748	1.1186E-05	0.000767	0.868483	0.000113	0.005626
14257	Flt4	C4	-0.75729	0.00045661	0.004939	0.868483	0.000484	0.009801
17300	Foxc1	C4	-0.79248	0.00025188	0.003616	0.868483	0.001634	0.017763
53318	Pdlim3	C4	-0.5	0.00381549	0.019314	0.868483	0.001281	0.01576
27421	Abcc6	C4	-0.66096	0.00382962	0.019343	0.868483	0.000403	0.009022
224419	Map3k7cl	C4	-0.73697	0.00253104	0.014747	0.868483	0.003596	0.027297
230157	Tmeff1	C4	-0.73697	0.00089528	0.007591	0.868483	0.003608	0.027366
140795	P2ry14	C4	-0.73697	0.00092765	0.007731	0.868483	0.000144	0.005928
233733	Galnt18	C4	-0.73697	0.00060914	0.005856	0.868483	0.001636	0.01775
50908	C1s1	C4	-0.73697	0.00253104	0.014738	0.868483	0.000965	0.013762
24058	Sigirr	C4	-0.77216	0.00190325	0.012161	0.868483	0.000312	0.007878
13636	Efna1	C4	-0.86848	0.00058231	0.005681	0.868483	0.000638	0.011066
407790	Ndufa4l2	C4	-0.86848	3.3185E-05	0.001229	0.868483	0.000534	0.01024
71721	Fam13c	C4	-0.86848	0.00022917	0.003395	0.868483	0.000419	0.009161
223864	Rapgef3	C4	-0.93723	1.6928E-05	0.000938	0.868483	0.00034	0.008258
545370	Hmcn1	C4	-0.97968	2.4198E-06	0.000467	0.868483	0.000223	0.006949
18189	Nrxn1	C4	-1	5.4129E-05	0.001627	0.868483	0.000546	0.010352
105387	Akr1c14	C4	-1	9.501E-06	0.000704	0.868483	0.000114	0.005677
11684	Alox12	C4	-1.02945	1.7574E-05	0.000951	0.868483	0.000517	0.010077

209047	Gipc3	C4	-1.1112	1.9227E-06	0.000424	0.868483	0.000304	0.007796
677884	Pakap	C4	-0.67634	0.00102125	0.008109	0.864098	0.000585	0.010644
121021	Cspg4	C4	-0.71425	0.00028078	0.003751	0.861233	0.000388	0.008782
75599	Pcdh1	C4	-0.83148	0.00013901	0.002681	0.861233	0.000565	0.010483
319565	Syne2	C4	-0.96644	8.2581E-06	0.000665	0.860691	0.002258	0.021166
233752	Insc	C4	-0.6531	0.00628565	0.027106	0.860619	7.66E-05	0.004802
209039	Tns2	C4	-0.62741	0.00129225	0.009511	0.858579	0.000483	0.009806
22003	Tpm1	C4	-0.49785	0.0035546	0.018375	0.855675	0.000235	0.007071
18845	Plxna2	C4	-0.589	6.5539E-05	0.001811	0.853017	0.000336	0.00822
20324	Cavin2	C4	-0.81339	1.5179E-05	0.000889	0.85022	0.000251	0.007205
12778	Ackr3	C4	-0.76553	4.0553E-05	0.001375	0.847997	0.000526	0.010187
105841	Dennd3	C4	-0.90531	2.6601E-05	0.001095	0.847573	0.000283	0.007533
17112	Tm4sf1	C4	-0.65519	0.00084796	0.007324	0.846996	0.000426	0.009251
53614	Reck	C4	-0.68926	0.00022457	0.003343	0.845581	0.000329	0.00818
19879	Slc22a8	C4	-0.90609	8.0916E-07	0.000341	0.844125	0.00035	0.008353
70873	Cnbd2	C4	-1.03356	0.0008498	0.007326	0.842249	0.000117	0.005672
77569	Limch1	C4	-0.79248	3.6562E-05	0.001292	0.841536	0.000299	0.007831
14083	Ptk2	C4	-0.73516	3.2426E-05	0.001225	0.840912	0.000168	0.00633
21846	Tie1	C4	-0.92194	0.00012755	0.002551	0.839779	0.000995	0.013923
106042	Prickle1	C4	-0.70752	1.9261E-05	0.00098	0.839036	0.000124	0.005763
15213	Hey1	C4	-0.86848	0.00028532	0.003774	0.839036	0.002627	0.02293
328949	Mcc	C4	-0.848	6.0844E-06	0.000569	0.839036	0.000325	0.008112
80837	Rhoj	C4	-0.924	5.8289E-05	0.001697	0.839036	0.00126	0.015587
54195	Gucy1b1	C4	-0.65208	0.00034217	0.004213	0.835346	0.000706	0.011754
16542	Kdr	C4	-0.61079	0.00025025	0.003626	0.833288	0.000104	0.005609
18627	Per2	C4	-0.58496	0.00423178	0.020589	0.827676	1.05E-05	0.002497
109700	Itga1	C4	-0.65236	0.00103571	0.00821	0.827252	0.000374	0.008591
69601	Dab2ip	C4	-0.6289	0.00039108	0.004532	0.826988	0.000477	0.009842
11647	Alpl	C4	-0.90548	7.7574E-05	0.001931	0.826038	0.000244	0.007138
72148	Tdrp	C4	-0.93723	1.5573E-05	0.000896	0.826038	0.000418	0.00918
230857	Ece1	C4	-0.56803	0.00153253	0.010628	0.825029	6.67E-05	0.004696
384569	Nova2	C4	-0.79248	0.00310866	0.01695	0.821928	0.001701	0.018126
20672	Sox18	C4	-0.8488	0.00180843	0.011833	0.819705	0.006001	0.036809
98845	Eps8l2	C4	-0.74271	0.00019339	0.003163	0.818715	0.000508	0.010024
55990	Fmo2	C4	-0.97968	1.7648E-05	0.000939	0.818715	0.001104	0.014575
14238	Foxf2	C4	-0.69452	0.00283396	0.015902	0.815025	0.001127	0.014662
54135	Lsr	C4	-0.69796	0.00035394	0.004312	0.811903	0.000662	0.011335
22288	Utrn	C4	-0.5024	0.00189051	0.012155	0.811536	0.000379	0.008666
11898	Ass1	C4	-0.48543	8.9879E-05	0.002112	0.811465	0.000473	0.009803
20742	Sptbn1	C4	-0.57507	0.00028414	0.003774	0.808763	0.000303	0.007824
18767	Pkia	C4	-0.58496	1.3127E-05	0.000805	0.807355	0.000312	0.007859
329628	Fat4	C4	-0.79248	2.2012E-05	0.001035	0.807355	0.000551	0.01034

171463	Il17rd	C4	-0.80735	2.5849E-05	0.001103	0.807355	0.001584	0.017517
29856	Smtn	C4	-0.63374	0.00146073	0.010316	0.805717	0.000381	0.008689
14282	Fosb	C4	-1.02571	0.00020019	0.003218	0.802936	0.003459	0.026731
171469	Gpr37l1	C4	-0.89605	0.00035503	0.004319	0.802816	0.00226	0.021155
209378	Itih5	C4	-0.78305	4.5399E-05	0.001465	0.800555	0.000279	0.007539
18096	Nkx6-1	C4	-0.51021	0.00120467	0.009097	0.798026	0.00048	0.009838
218952	Fermt2	C4	-0.73733	0.00018684	0.003111	0.797473	0.000915	0.013503
114255	Dok4	C4	-0.52945	0.00126462	0.009389	0.792481	0.000622	0.010879
21385	Tbx2	C4	-0.58496	0.00075376	0.006756	0.792481	0.000608	0.010783
16535	Kcnq1	C4	-0.58496	0.00041726	0.004711	0.792481	0.001536	0.017297
72293	Nkd2	C4	-0.58496	0.00048985	0.00516	0.792481	0.002352	0.021462
58187	Cldn10	C4	-0.58496	0.01276027	0.043546	0.792481	0.003515	0.026934
170441	Slc2a10	C4	-0.58496	0.01037971	0.037731	0.792481	0.001702	0.018117
16772	Lama1	C4	-0.58496	0.00092919	0.007723	0.792481	0.000616	0.010812
66949	Trim59	C4	-0.58496	0.00167403	0.01128	0.792481	0.000341	0.008244
272381	Lrrc4b	C4	-0.58496	0.01092628	0.039013	0.792481	0.000465	0.009731
14239	Foxs1	C4	-0.58496	0.00041726	0.00466	0.792481	0.001702	0.018097
101359	Prrt4	C4	-0.58496	0.00091459	0.00767	0.792481	0.002352	0.021442
17130	Smad6	C4	-0.68129	0.00011537	0.002406	0.792481	0.001521	0.01726
12832	Col5a2	C4	-0.69616	0.00070931	0.006489	0.792481	0.001287	0.015775
26903	Dysf	C4	-0.69616	0.00059231	0.005748	0.792481	0.001211	0.015329
76574	Mfsd2a	C4	-0.72972	0.0002653	0.003716	0.792481	0.000819	0.012804
70435	Inf2	C4	-0.75548	3.4816E-05	0.001254	0.792481	0.000152	0.006163
13371	Dio2	C4	-0.79248	0.00016708	0.002952	0.792481	0.003845	0.0285
20969	Sdc1	C4	-0.79248	0.00199429	0.01251	0.792481	2.03E-05	0.003364
239530	Gpr20	C4	-0.79248	0.00090627	0.007649	0.792481	0.006653	0.038885
235527	Plscr4	C4	-0.80735	4.1471E-05	0.001386	0.792481	0.000494	0.009872
28250	Slco1a4	C4	-0.85343	4.0205E-06	0.000477	0.792481	0.00027	0.007516
16426	Itih3	C4	-0.90368	9.917E-05	0.002234	0.792481	0.000581	0.010612
12373	Casq2	C4	-0.90368	3.7361E-05	0.001305	0.792481	0.000588	0.010675
269132	Colgalt2	C4	-0.95345	2.688E-05	0.001096	0.792481	0.00274	0.023313
30937	Lmcd1	C4	-1.16096	6.4589E-05	0.00179	0.792481	0.003867	0.028597
433182	Eno1b	C4	-1.35022	0.00030746	0.003948	0.792481	0.00296	0.024437
76453	Prss23	C4	-0.71442	0.00032182	0.00407	0.792481	0.002448	0.021958
22352	Vim	C4	-0.50583	0.01200756	0.04156	0.788775	0.000605	0.010775
13602	Sparcl1	C4	-0.78044	1.8148E-05	0.000955	0.787464	0.000624	0.01089
60596	Gucy1a1	C4	-0.66695	0.00025038	0.003622	0.787454	0.00071	0.01176
234214	Sorbs2	C4	-0.68247	0.00011639	0.002411	0.782125	0.000603	0.010769
14605	Tsc22d3	C4	-0.6256	0.00084153	0.007295	0.781602	0.000105	0.005587
15507	Hspb1	C4	-0.63463	0.00763241	0.030953	0.780944	0.003975	0.02911
18596	Pdgfrb	C4	-0.69425	0.00029629	0.003847	0.780697	0.001058	0.014322
20525	Slc2a1	C4	-0.60625	0.00338982	0.017883	0.776342	0.000833	0.012876

15953	Ifi47	C4	-0.53519	0.00289671	0.016185	0.776271	0.000307	0.007831
17988	Ndrg1	C4	-0.58816	0.00136011	0.009861	0.775181	0.000842	0.012994
97064	Wwtr1	C4	-0.62899	0.00076732	0.006838	0.773744	0.00069	0.011577
68519	Eml1	C4	-0.72239	0.00129297	0.009508	0.77216	0.001907	0.019215
16450	Jag2	C4	-0.6563	0.0018216	0.011894	0.768828	0.000805	0.012743
244853	Nxpe4	C4	-0.6112	0.00078556	0.006933	0.765257	0.000537	0.010271
66864	Clec14a	C4	-0.87447	4.939E-05	0.00154	0.765257	0.001589	0.017485
12390	Cav2	C4	-0.60429	0.00024782	0.003602	0.765257	0.000332	0.00823
57278	Bcam	C4	-0.69467	0.00026145	0.003684	0.76495	0.000931	0.013597
545428	Ccdc141	C4	-0.79364	1.814E-06	0.000442	0.764189	0.000589	0.010672
21859	Timp3	C4	-0.56172	0.00075045	0.006733	0.763452	0.001797	0.018551
170643	Kirrel	C4	-0.66671	0.00010686	0.002317	0.763034	0.00177	0.018423
12169	Bmx	C4	-0.83904	0.00189767	0.012159	0.763034	0.006284	0.03772
319876	Cobll1	C4	-0.79248	1.2945E-05	0.000799	0.761781	0.000271	0.007492
75646	Rai14	C4	-0.52418	0.01555391	0.049743	0.758924	0.000607	0.010773
235633	Als2cl	C4	-0.62577	0.00187157	0.012092	0.757287	0.000907	0.013443
99526	Usp53	C4	-0.54977	0.00046597	0.004999	0.757287	0.000277	0.00754
18483	Palm	C4	-0.74593	9.3394E-05	0.002162	0.753369	0.001624	0.017697
208177	Phldb2	C4	-0.7187	8.3568E-06	0.000661	0.75125	0.001749	0.018278
215114	Hip1	C4	-0.48745	0.00976793	0.036305	0.749786	0.000521	0.01012
268780	Egflam	C4	-0.67807	0.00027625	0.003718	0.742713	0.004515	0.03113
56233	Hdac7	C4	-0.70148	0.00038671	0.004521	0.742713	0.000678	0.011481
68169	Ndnf	C4	-0.83904	3.7972E-06	0.000502	0.742713	0.000232	0.007023
319899	Dock6	C4	-0.78927	1.3525E-05	0.000813	0.742713	0.0003	0.007803
14184	Fgfr3	C4	-0.97278	1.1314E-05	0.000765	0.736966	0.001913	0.019258
20356	Sema5a	C4	-0.576	0.00596302	0.026153	0.736966	0.002134	0.020549
71406	Spaar	C4	-0.576	0.00398213	0.0198	0.736966	0.00478	0.032185
76527	Il34	C4	-0.576	0.00085909	0.007385	0.736966	0.002335	0.021374
72690	Fam110d	C4	-0.576	0.01485101	0.048144	0.736966	0.009883	0.048737
100039781	Hrct1	C4	-0.86848	0.00066818	0.006236	0.736966	0.00579	0.036013
20620	Plk2	C4	-0.67682	0.00027042	0.003715	0.734743	5.43E-05	0.004539
353156	Egfl7	C4	-0.86051	6.1217E-05	0.001739	0.734743	0.00097	0.013821
77446	Heg1	C4	-0.65873	0.00038687	0.004517	0.730727	0.001618	0.017692
105844	Card10	C4	-0.60751	0.00177228	0.011679	0.729716	0.000664	0.011352
338367	Myo1d	C4	-0.72972	0.00053337	0.005362	0.729716	0.001457	0.016749
17207	Mcf2l	C4	-0.68532	0.00042303	0.004708	0.723729	0.002041	0.020133
233335	Synm	C4	-0.75899	3.9625E-06	0.000476	0.722305	0.000593	0.01069
12389	Cav1	C4	-0.73927	0.00019086	0.003133	0.721803	0.000944	0.013649
68481	Mpzl1	C4	-0.73697	0.00034141	0.004209	0.72055	0.002285	0.021289
53313	Atp2a3	C4	-0.65018	0.00067796	0.006289	0.719923	0.005448	0.034997
74090	Paqr5	C4	-0.72239	2.761E-05	0.001111	0.718703	0.001259	0.015595
12554	Cdh13	C4	-0.83904	2.9092E-05	0.001151	0.71648	0.002527	0.022376

78751	Zc3h6	C4	-0.924	0.00018551	0.0031	0.71648	0.003443	0.026713
20617	Snca	C4	-0.91745	0.00210477	0.012974	0.709929	0.003192	0.025624
211945	Plekhh1	C4	-0.576	0.00398213	0.01979	0.707519	0.006245	0.037645
233651	Dchs1	C4	-0.63152	7.681E-05	0.001927	0.707519	0.000878	0.0132
241226	Itga8	C4	-0.70752	0.00036975	0.004406	0.707519	0.010285	0.049868
69219	Ddah1	C4	-0.70752	0.00014231	0.002717	0.707519	0.002345	0.021426
243864	Mill2	C4	-0.70752	0.00129982	0.009521	0.707519	0.002137	0.020497
399548	Scn4b	C4	-0.70752	0.00694525	0.029096	0.707519	0.005023	0.033295
12291	Cacna1g	C4	-0.86848	5.4627E-05	0.001637	0.707519	0.004326	0.030405
13195	Ddc	C4	-0.97968	4.1625E-06	0.000476	0.707519	0.000693	0.011618
381823	Apold1	C4	-0.57565	0.00897199	0.034269	0.704042	0.002263	0.021163
29817	Igfbp7	C4	-0.5421	0.00264842	0.01523	0.703062	0.002015	0.019916
15901	Id1	C4	-0.7022	1.5806E-05	0.000898	0.702195	0.003268	0.025955
51789	Tnk2	C4	-0.70826	3.8386E-05	0.001326	0.701532	0.001012	0.013969
14268	Fn1	C4	-0.60146	0.00295266	0.016379	0.698193	0.004409	0.030724
54525	Syt7	C4	-0.58496	0.0057667	0.025509	0.696159	0.005455	0.034975
78514	Arhgap10	C4	-0.69616	0.00051831	0.005262	0.696159	0.000435	0.009351
118449	Synpo2	C4	-0.79248	2.9623E-05	0.001162	0.696159	0.000597	0.010705
50768	Dlc1	C4	-0.47364	0.00317406	0.017165	0.690052	0.001649	0.017831
17929	Myom1	C4	-0.47243	0.00988995	0.036553	0.689256	0.000474	0.009806
276919	Gemin4	C4	-0.79248	0.00017987	0.003044	0.689256	0.000546	0.01034
56332	Amotl2	C4	-0.76872	6.9765E-06	0.000615	0.688485	0.000251	0.007188
18830	Pltp	C4	-0.91156	2.5797E-06	0.000468	0.679997	0.000761	0.012319
16775	Lama4	C4	-0.77627	0.00015927	0.002869	0.679948	0.001594	0.017528
21807	Tsc22d1	C4	-0.72553	0.0001196	0.002444	0.679499	0.002285	0.021269
15530	Hspg2	C4	-0.8216	3.0646E-06	0.000473	0.678897	0.001175	0.015091
102747	Lrrc49	C4	-0.924	1.4227E-06	0.000399	0.678072	0.001202	0.015239
66860	Tanc1	C4	-0.77216	1.1269E-05	0.000767	0.668935	0.000975	0.013842
56198	Heyl	C4	-0.58175	3.3995E-05	0.001239	0.666712	0.001096	0.014569
11603	Agrn	C4	-0.48227	0.00763505	0.03095	0.66325	0.00061	0.010766
74103	Nebl	C4	-0.60768	0.00017452	0.002987	0.663194	0.001585	0.017504
12258	Serping1	C4	-0.5	0.00952004	0.035714	0.660964	0.002848	0.023794
19223	Ptgis	C4	-0.52945	0.00426569	0.020721	0.660964	0.007352	0.040825
103967	Dnm3	C4	-0.66096	0.00154044	0.010667	0.660964	0.005162	0.033874
70393	2210416O15Rik	C4	-0.73697	3.8081E-06	0.000497	0.660964	0.001422	0.016559
23805	Apc2	C4	-0.77216	0.0002729	0.003721	0.660964	0.005525	0.035261
330450	Far2	C4	-0.79248	0.00108082	0.008452	0.660964	0.006253	0.037627
230971	Megf6	C4	-0.79248	0.00031916	0.004053	0.660964	0.005276	0.034346
19091	Prkg1	C4	-0.90368	1.262E-05	0.0008	0.660964	0.002076	0.020315
68312	Gstm7	C4	-1.1112	2.3746E-06	0.000468	0.660964	0.007924	0.042904
13846	Ephb4	C4	-0.48899	0.00470673	0.022245	0.659505	0.003201	0.025649
50780	Rgs3	C4	-0.72561	6.2869E-05	0.001775	0.656117	0.000227	0.006996

16975	Lrp8	C4	-0.63152	5.0207E-05	0.001549	0.654061	0.001882	0.019043
68337	Crip2	C4	-0.76883	2.2175E-05	0.001032	0.653166	0.001452	0.016759
18073	Nid1	C4	-0.46644	0.00582008	0.02566	0.653052	0.001221	0.01534
16779	Lamb2	C4	-0.56731	0.00198199	0.012483	0.651418	0.001218	0.01534
20312	Cx3cl1	C4	-0.73581	0.00020947	0.003238	0.647372	0.006175	0.037525
70747	Tspan2	C4	-0.47243	0.00338721	0.017879	0.646391	0.002886	0.024008
13405	Dmd	C4	-0.58496	0.00066925	0.00624	0.644753	0.001725	0.018226
277010	Marveld1	C4	-0.51874	0.00221203	0.013386	0.644063	0.001469	0.016847
16329	Inpp1	C4	-0.48899	0.00198924	0.012503	0.638767	0.000548	0.010339
17356	Afdn	C4	-0.50499	0.00161382	0.010994	0.634849	0.00061	0.010758
20536	Slc4a3	C4	-0.53519	0.00262846	0.015125	0.631517	0.005295	0.034446
330938	Dixdc1	C4	-0.6112	0.00053408	0.005363	0.631517	0.001129	0.014656
60345	Nrip2	C4	-0.64639	0.00061636	0.005901	0.631517	0.001727	0.018191
20447	St6galnac3	C4	-0.64639	0.0010377	0.008212	0.631517	0.00138	0.016204
14261	Fmo1	C4	-0.74271	0.0003853	0.004516	0.631517	0.001155	0.014873
17161	Maoa	C4	-0.66507	0.00010975	0.002341	0.631517	0.001213	0.015328
56213	Htra1	C4	-0.8795	8.9021E-05	0.002103	0.631517	0.005652	0.035631
22370	Vtn	C4	-0.6603	2.3312E-05	0.001043	0.629596	0.002054	0.020175
53901	Rcan2	C4	-0.77258	6.0654E-05	0.001733	0.627827	0.003127	0.025243
17909	Myo10	C4	-0.78894	3.3125E-06	0.000487	0.626194	0.00109	0.014532
15129	Hbb-b1	C4	-1.14114	2.0521E-05	0.001016	0.625995	0.002628	0.022916
22330	Vcl	C4	-0.58118	0.00178674	0.011732	0.625272	0.002166	0.020651
68070	Pdzd2	C4	-0.70119	6.7631E-06	0.000608	0.62476	0.003022	0.024762
232333	Slc6a1	C4	-0.89716	0.0003778	0.004456	0.62476	0.005116	0.033682
225608	Sh3tc2	C4	-0.62445	0.0002496	0.003622	0.624453	0.005492	0.0351
20511	Slc1a2	C4	-0.8289	0.00026656	0.003717	0.623964	0.00841	0.044405
98660	Atp1a2	C4	-0.8714	1.7838E-06	0.000446	0.61941	0.002657	0.02303
229699	Slc16a4	C4	-0.82604	2.0934E-06	0.00044	0.61852	0.001665	0.017903
23849	Klf6	C4	-0.77	2.636E-05	0.001099	0.617474	0.00768	0.042001
16206	Lrig1	C4	-0.90368	6.29E-06	0.000582	0.615477	0.002137	0.020482
242202	Pde5a	C4	-0.47968	0.00265462	0.015181	0.611196	0.008027	0.043224
17472	Gbp4	C4	-0.79248	0.00149001	0.010451	0.611196	0.005195	0.034021
224129	Adcy5	C4	-0.86848	6.3055E-06	0.000578	0.611196	0.003492	0.026858
19283	Ptprz1	C4	-0.66096	0.00157155	0.010818	0.609983	0.004682	0.031837
18260	Ocln	C4	-0.62396	1.623E-05	0.000916	0.606497	0.001991	0.019739
101488143	Hbb-bt	C4	-1.0982	5.8018E-05	0.001695	0.60516	0.002333	0.021373
407823	Baz2b	C4	-0.49874	0.01183265	0.041077	0.602723	0.00065	0.011209
29820	Tnfrsf19	C4	-0.68187	7.3217E-06	0.00064	0.59897	0.001213	0.015299
12822	Col18a1	C4	-0.59897	0.00059153	0.005759	0.596912	0.001312	0.015864
26938	St6galnac5	C4	-0.80061	0.00035735	0.004325	0.59613	0.002837	0.023761
20393	Sgk1	C4	-0.66675	2.2548E-05	0.001039	0.59493	5.47E-05	0.004532
13653	Egr1	C4	-0.94415	3.1266E-05	0.001206	0.594734	0.007116	0.040152

216505	Pik3ip1	C4	-0.63402	8.6178E-05	0.002056	0.592933	0.001323	0.015865
110257	Hba-a2	C4	-1.13425	4.9847E-05	0.001544	0.592383	0.003143	0.02533
77739	Adamtsl1	C4	-0.81871	0.00017076	0.002961	0.591296	0.00872	0.045358
17131	Smad7	C4	-0.82393	1.1044E-05	0.000763	0.58977	0.003702	0.027754
69068	Tcim	C4	-0.45943	0.00779772	0.031417	0.584963	0.00085	0.013029
211914	Asap2	C4	-0.576	0.00034618	0.00424	0.584963	0.00882	0.045637
30805	Slc22a4	C4	-0.58496	0.00092919	0.007729	0.584963	0.000279	0.007548
18552	Pcsk5	C4	-0.58496	0.01037971	0.037746	0.584963	0.006673	0.038887
51800	Bok	C4	-0.58496	0.00091459	0.007698	0.584963	0.010129	0.049479
11444	Chrnb2	C4	-0.58496	0.01442715	0.047316	0.584963	0.000179	0.006413
23948	Mmp17	C4	-0.58496	0.00900301	0.034302	0.584963	0.007753	0.042281
12515	Cd69	C4	-0.58496	0.00048985	0.005136	0.584963	0.009264	0.047135
110891	Slc8a2	C4	-0.58496	0.00926837	0.035011	0.584963	0.005755	0.035954
170826	Ppargc1b	C4	-0.58496	0.01442715	0.047283	0.584963	0.000207	0.006854
21390	Tbxa2r	C4	-0.58496	0.00091459	0.007691	0.584963	0.007937	0.042928
211134	Lzts1	C4	-0.58496	0.00041726	0.004705	0.584963	0.007937	0.042904
229011	Samd10	C4	-0.58496	0.00048985	0.005119	0.584963	0.000179	0.006389
68810	Nexn	C4	-0.58496	0.01442715	0.047266	0.584963	0.000179	0.006366
215798	Adgrg6	C4	-0.58496	0.00041726	0.004694	0.584963	0.008624	0.045072
214359	Tmem51	C4	-0.58496	0.00010131	0.00226	0.584963	0.000532	0.010248
546336	Prrg1	C4	-0.58496	0.00041726	0.004677	0.584963	0.001213	0.01531
17760	Map6	C4	-0.58496	0.00041726	0.004671	0.584963	0.008624	0.045048
58804	Cdc42ep5	C4	-0.58496	0.00048985	0.005096	0.584963	0.000485	0.009747
211378	6720489N17Rik	C4	-0.58496	0.01442715	0.047233	0.584963	0.009789	0.048444
269389	Tox2	C4	-0.58496	0.01442715	0.047216	0.584963	0.000471	0.009783
77994	Mir99ahg	C4	-0.58496	0.01038704	0.037625	0.584963	0.009789	0.04842
629159	1700008J07Rik	C4	-0.58496	0.01442715	0.047166	0.584963	0.006673	0.038726
666257	Zfp660	C4	-0.58496	0.00037204	0.004422	0.584963	0.000808	0.012729
320736	Vstm4	C4	-0.69616	0.00045663	0.004933	0.584963	0.003592	0.027351
242425	Gabbr2	C4	-0.79248	0.00028913	0.003797	0.584963	0.005251	0.034297
225187	Ankrd29	C4	-0.79248	3.7268E-05	0.001307	0.584963	0.007123	0.040169
14615	Gjc1	C4	-0.83904	2.1173E-05	0.001026	0.584963	0.005386	0.034738
18795	Plcb1	C4	-0.924	8.8413E-07	0.000341	0.584963	0.003374	0.026412
13106	Cyp2e1	C4	-1	6.4723E-07	0.000428	0.584963	0.010129	0.049504
109979	Art3	C4	-1	1.9041E-06	0.000441	0.584963	0.001624	0.01771
319670	Eml5	C4	-1	1.9041E-06	0.00043	0.584963	0.007937	0.042881
104445	Cdc42ep1	C4	-0.72403	7.3788E-05	0.001903	0.583972	0.003025	0.024743
74354	Lrguk	C4	-0.55552	0.00334999	0.017764	0.581749	5.79E-05	0.004572
74229	Paqr8	C4	-0.61325	0.00075966	0.006789	0.581749	0.006216	0.037588
14696	Gnb4	C4	-0.67807	1.6515E-05	0.000927	0.581749	0.00388	0.028651
66066	Gng11	C4	-0.67807	0.00065338	0.006154	0.581749	0.001855	0.018848
18217	Ntsr2	C4	-0.76303	6.3788E-05	0.00179	0.581749	0.003499	0.026851

239719	Mrtfb	C4	-0.48465	0.00250034	0.014624	0.580732	0.00078	0.012518
18952	Septin4	C4	-0.70645	0.00029495	0.003846	0.580681	0.00349	0.026868
268878	Atp13a5	C4	-0.5	0.00060575	0.005848	0.576002	0.004424	0.030809
72147	Zbtb46	C4	-0.54843	0.00354151	0.018359	0.576002	0.001947	0.019414
74194	Rnd3	C4	-0.576	0.00126558	0.009389	0.576002	0.000593	0.010651
67009	Ttc23	C4	-0.576	0.00039674	0.00458	0.576002	0.000497	0.0099
12337	Capn5	C4	-0.576	0.00084719	0.007324	0.576002	0.000605	0.010771
11676	Aldoc	C4	-0.71618	0.00073982	0.006657	0.575797	0.007164	0.040305
226251	Ablim1	C4	-0.58262	7.4688E-05	0.001916	0.575332	0.001116	0.014642
208449	Sgms1	C4	-0.50421	0.00142539	0.010128	0.568368	0.004165	0.029941
109294	Prex2	C4	-0.74526	6.0613E-05	0.001737	0.56821	0.003011	0.024715
70445	Cd248	C4	-0.58496	0.00200836	0.012564	0.564642	0.009088	0.046504
380713	Scarf1	C4	-0.69616	0.00025829	0.003662	0.564642	0.00841	0.044382
217410	Trib2	C4	-0.90368	4.9037E-06	0.000547	0.564642	0.008138	0.043628
66425	Pcp4l1	C4	-0.81946	4.1452E-06	0.00048	0.563556	0.001853	0.018862
12032	Bcan	C4	-0.97968	4.681E-05	0.001494	0.557739	0.008309	0.044086
216551	Lgalsl	C4	-0.56277	0.00012597	0.00253	0.555212	0.004613	0.03152
54403	Slc4a4	C4	-0.59897	0.00360552	0.018566	0.554467	0.009054	0.046451
13998	Fgd6	C4	-0.47377	0.004006	0.019866	0.549768	0.001223	0.015339
12818	Col14a1	C4	-0.72972	8.8363E-06	0.000693	0.549768	3.14E-05	0.004095
15122	Hba-a1	C4	-1.16508	4.3908E-05	0.001431	0.548289	0.004307	0.03034
93742	Pard3	C4	-0.924	9.0122E-06	0.000695	0.546555	0.006667	0.038944
98402	Sh3bp4	C4	-0.54549	0.001432	0.010136	0.545487	0.003631	0.027366
23917	Impdh1	C4	-0.60932	0.00021532	0.003273	0.544503	0.006201	0.03759
12925	Crip1	C4	-0.52502	0.00395377	0.019744	0.541231	0.002992	0.024597
17281	Fyco1	C4	-0.46897	0.00311427	0.016971	0.539058	0.001412	0.016526
20708	Serpinb6b	C4	-0.53519	0.00264871	0.015222	0.535195	0.000649	0.011219
19242	Ptn	C4	-0.65364	9.0589E-05	0.002123	0.534474	0.00205	0.020174
268709	Fam107a	C4	-0.45097	0.00342618	0.017972	0.532548	0.008564	0.044879
57776	Ttyh1	C4	-0.55791	0.00204286	0.012737	0.531677	0.007403	0.040991
207212	Arhgef17	C4	-0.69716	1.7593E-05	0.000947	0.529953	0.004295	0.030344
237775	Zfp867	C4	-0.52945	0.00046638	0.004998	0.529447	0.00705	0.039987
224829	Trerf1	C4	-0.52945	4.4626E-05	0.00145	0.529447	0.007098	0.040144
208659	Fam20a	C4	-0.66096	0.00157286	0.010819	0.529447	0.004682	0.031858
237504	Rassf9	C4	-0.86848	8.9433E-06	0.000696	0.529447	0.009585	0.047995
20358	Sema6a	C4	-0.90368	1.262E-05	0.000806	0.529447	0.003277	0.025964
72061	Aopep	C4	-0.47278	2.0475E-05	0.001025	0.526234	1.37E-05	0.002682
29875	Iqgap1	C4	-0.56199	0.0051852	0.023778	0.524675	0.007172	0.040304
100213	Rusc2	C4	-0.48891	0.00698195	0.029184	0.524455	0.000439	0.009391
18798	Plcb4	C4	-0.64475	7.3237E-05	0.001926	0.522544	0.00628	0.037745
58996	Arhgap23	C4	-0.51621	0.00052582	0.005315	0.521534	0.001371	0.016144
72536	Tagap	C4	-0.50698	0.00213454	0.013062	0.521372	0.004621	0.031532

71729	Rgs12	C4	-0.67215	1.2914E-05	0.000808	0.518087	0.002174	0.02069
56448	Cyp2d22	C4	-0.83535	1.8628E-06	0.000442	0.514573	0.005458	0.034928
54169	Kat6b	C4	-0.45088	0.00834058	0.032707	0.511028	0.003537	0.027037
18484	Pam	C4	-0.72659	5.8296E-06	0.000568	0.508537	0.005069	0.03349
70292	Afap1	C4	-0.45023	0.00074588	0.006698	0.507971	0.002477	0.022114
18710	Pik3r3	C4	-0.63842	0.00072968	0.006617	0.506903	0.006574	0.038652
58807	Slco1c1	C4	-0.66848	3.1786E-05	0.001216	0.505377	0.005574	0.035416
13731	Emp2	C4	-0.70341	1.7351E-05	0.000951	0.503116	0.003151	0.025353
75723	Amotl1	C4	-0.45301	0.00126626	0.009379	0.502359	0.001693	0.018083
170459	Stard4	C4	-0.6112	0.00045126	0.00491	0.5	0.008391	0.044356
217721	Flvcr2	C4	-0.58496	0.00012473	0.002516	0.5	0.008309	0.04411
16826	Ldb2	C4	-0.58496	5.4914E-06	0.000565	0.5	0.008436	0.044471
76252	Atp6v0e2	C4	-0.66096	0.00047379	0.005048	0.5	0.00794	0.042873
14221	Fjx1	C4	-0.66096	0.00100707	0.00808	0.5	0.004372	0.030557
269233	Fam171a1	C4	-0.66893	2.3531E-05	0.001047	0.5	0.005562	0.035409
108069	Grm3	C4	-0.79248	0.00044621	0.004866	0.5	0.003673	0.027578
70598	Filip1	C4	-0.80735	5.5514E-06	0.000565	0.5	0.01024	0.049724
72014	Btbd17	C4	-0.86848	9.3622E-05	0.002162	0.5	0.005455	0.034953
14658	Glrb	C4	-0.86848	0.00033502	0.00418	0.5	0.009288	0.047231
67302	Zc3h13	C4	-0.53401	0.00165803	0.011205	0.497508	0.006315	0.037838
56338	Txnip	C4	-0.68738	0.00032848	0.004143	0.493022	0.008204	0.04379
20411	Sorbs1	C4	-0.57927	0.00102735	0.008151	0.488987	0.008484	0.044627
100604	Lrrc8c	C4	-0.57628	0.00027482	0.003714	0.485993	0.003746	0.027938
228071	Sestd1	C4	-0.48543	6.8855E-05	0.001853	0.485427	0.001921	0.019314
12558	Cdh2	C4	-0.58175	0.00030821	0.003952	0.485427	0.004441	0.030817
67306	Zc2hc1a	C4	-0.67807	0.00038689	0.004511	0.485427	0.009625	0.048121
243362	Stard13	C4	-0.71648	8.6556E-07	0.000348	0.485427	0.005982	0.036762
15936	Ier2	C4	-0.91179	1.459E-05	0.00086	0.483268	0.00385	0.028518
19679	Pitpnm2	C4	-0.52829	6.4188E-05	0.00179	0.481737	0.005128	0.033741
67916	Plpp3	C4	-0.73251	0.00025728	0.003653	0.477032	0.005535	0.035301
27494	Amot	C4	-0.67626	1.8048E-05	0.000955	0.473766	0.007343	0.040821
12953	Cry2	C4	-0.58397	1.0061E-05	0.000711	0.472776	0.000707	0.011741
20410	Sorbs3	C4	-0.55552	0.00040307	0.004619	0.470553	0.004376	0.030562
21808	Tgfb2	C4	-1.1541	6.9014E-07	0.000399	0.467255	0.002804	0.023593
234094	Arhgef10	C4	-0.56464	0.00083182	0.007231	0.459432	0.003639	0.027406
2044620446	#N/A	C4	-0.76966	7.8927E-06	0.000658	0.455029	0.010237	0.049732
81840	Sorcs2	C4	-0.56464	0.00497007	0.023077	0.453445	0.003229	0.025812
17974	Nck2	C4	-0.58496	0.00224978	0.013552	0.453445	0.00942	0.04753

Supplementary Table 6 Gene set enrichment analysis of gene ontology (GO) terms for the cluster. The table shows information on the genes, grouped by cluster.

Microglial phagocytosis		Immune system process		Cytokine production		Response to cytokine stimulus		Cell differentiation	
**Entrez id**	**Symbol**	**Entrez id**	**Symbol**	**Entrez id**	**Symbol**	**Entrez id**	**Symbol**	**Entrez id**	**Symbol**
17064	Cd93	17002	Ltf	17002	Ltf	16176	Il1b	17002	Ltf
14083	Ptk2	16176	Il1b	18124	Nr4a3	213002	Ifitm6	16176	Il1b
17909	Myo10	16153	Il10	16176	Il1b	20310	Cxcl2	16153	Il10
14461	Gata2	20971	Sdc4	16153	Il10	215257	Il36g	74748	Slamf8
16440	Itpr3	56792	Stap1	12051	Bcl3	17533	Mrc1	21950	Tnfsf9
108100	Baiap2	170741	Pilrb1	21950	Tnfsf9	12363	Casp4	215257	Il36g
16071	Igkc	114332	Lyve1	215257	Il36g	19332	Rab20	12262	C1qc
11540	Adora2a	16415	Itgb2l	269799	Clec4a1	56792	Stap1	15982	Ifrd1
211914	Asap2	15205	Hes1	12363	Casp4	20306	Ccl7	242253	Dnai3
103967	Dnm3	12475	Cd14	19074	Prg2	12768	Ccr1	17133	Maff
17909	Myo10	68891	Cd177	56620	Clec4n	56744	Pf4	228839	Tgif2
18710	Pik3r3	18124	Nr4a3	12774	Ccr5	14825	Cxcl1	17173	Ascl2
67916	Plpp3	67278	Pagr1a	12796	Camp	56221	Ccl24	56792	Stap1
11540	Adora2a	74748	Slamf8	12475	Cd14	15205	Hes1	170741	Pilrb1
17064	Cd93	20201	S100a8	18106	Cd244a	12796	Camp	12768	Ccr1
26903	Dysf	21950	Tnfsf9	11689	Alox5			56744	Pf4
14461	Gata2	12262	C1qc	12655	Chil3			54720	Rcan1
16071	Igkc	12363	Casp4					15205	Hes1
73182	Pear1	12263	C2					12156	Bmp2
14083	Ptk2	19074	Prg2					12774	Ccr5
54525	Syt7	224840	Treml4					12569	Cdk5r1
78514	Arhgap10	17173	Ascl2					11689	Alox5
13601	Ecm1	20306	Ccl7					76905	Lrg1
14268	Fn1	384059	Tlr12						
26570	Slc7a11	12768	Ccr1						
22352	Vim	56744	Pf4						
23917	Impdh1	14825	Cxcl1						
16592	Fabp5	56221	Ccl24						
77446	Heg1	56620	Clec4n						
18008	Nes	12047	Bcl2a1d						
229003	Helz2	18106	Cd244a						
20893	Bhlhe40								
70097	Sash1								
228071	Sestd1								
72535	Aldh1b1								
11987	Slc7a1								
108100	Baiap2								
14102	Fas								
18810	Plec								

21366	Slc6a6								
**Microglial migration**		**Microglial mobility**		**Locomotion**		**Cell adhesion**		**Chemotaxis**	
**Entrez id**	**Symbol**	**Entrez id**	**Symbol**	**Entrez id**	**Symbol**	**Entrez id**	**Symbol**	**Entrez id**	**Symbol**
67784	Plxnd1	67784	Plxnd1	67784	Plxnd1	67784	Plxnd1	67784	Plxnd1
94246	Arid4b	94246	Arid4b	94246	Arid4b	18377	Omg	218877	Sema3g
218952	Fermt2	218952	Fermt2	218952	Fermt2	218952	Fermt2	14254	Flt1
16476	Jun	16476	Jun	12390	Cav2	18132	Notch4	18131	Notch3
75723	Amotl1	75723	Amotl1	18131	Notch3	93711	Pcdhga3	13482	Dpp4
72333	Palld	72333	Palld	75723	Amotl1	72333	Palld	23928	Lamc3
13482	Dpp4	13482	Dpp4	72333	Palld	13482	Dpp4	16590	Kit
69601	Dab2ip	69601	Dab2ip	13482	Dpp4	12032	Bcan	15507	Hspb1
22352	Vim	22352	Vim	69601	Dab2ip	106042	Prickle1	13609	S1pr1
223864	Rapgef3	223864	Rapgef3	223864	Rapgef3	16590	Kit	12554	Cdh13
18096	Nkx6-1	18096	Nkx6-1	78560	Adgra2	11987	Slc7a1	107449	Unc5b
106042	Prickle1	106042	Prickle1	74194	Rnd3	12554	Cdh13	18810	Plec
16590	Kit	16590	Kit	243362	Stard13	12558	Cdh2	319504	Nrcam
78560	Adgra2	78560	Adgra2	18810	Plec	13405	Dmd	20348	Sema3c
74194	Rnd3	74194	Rnd3	14219	Ccn2	14219	Ccn2	13617	Ednra
12554	Cdh13	12554	Cdh13	235041	Kank2	268780	Egflam	27494	Amot
243362	Stard13	243362	Stard13	14461	Gata2	68764	Cdhr3	13616	Edn3
21380	Tbx1	21380	Tbx1	20348	Sema3c	241226	Itga8	226251	Ablim1
18810	Plec	18810	Plec	54485	Dll4	227937	Pkp4	16007	Ccn1
12558	Cdh2	12558	Cdh2	16404	Itga7	22003	Tpm1	16842	Lef1
14219	Ccn2	14219	Ccn2	233651	Dchs1	20745	Spock1	14268	Fn1
235041	Kank2	235041	Kank2	14102	Fas	16404	Itga7	13614	Edn1
70445	Cd248	70445	Cd248	17974	Nck2	233651	Dchs1	20356	Sema5a
20620	Plk2	20620	Plk2	18823	Plp1	16007	Ccn1	20312	Cx3cl1
545428	Ccdc141	545428	Ccdc141	11754	Aoc3	12562	Cdh5	22418	Wnt5a
66864	Clec14a	66864	Clec14a	20312	Cx3cl1	14102	Fas	16773	Lama2
71653	Shtn1	71653	Shtn1	105450	Mmrn2	12169	Bmx	19242	Ptn
14461	Gata2	14461	Gata2	20672	Sox18	11475	Acta2	16772	Lama1
22003	Tpm1	22003	Tpm1	240185	Jcad	17974	Nck2	68810	Nexn
20745	Spock1	20745	Spock1	20671	Sox17	11754	Aoc3	18596	Pdgfrb
20348	Sema3c	20348	Sema3c	18596	Pdgfrb	20312	Cx3cl1	71660	Rarres2
27494	Amot	27494	Amot	121021	Cspg4	105450	Mmrn2	226519	Lamc1
54485	Dll4	54485	Dll4	20315	Cxcl12	93695	Gpnmb	20315	Cxcl12
22240	Dpysl3	22240	Dpysl3	11603	Agrn	240185	Jcad	20358	Sema6a
16404	Itga7	16404	Itga7	11606	Agt	68810	Nexn	11603	Agrn
13653	Egr1	13653	Egr1	320736	Vstm4	93699	Pcdhgb1	57764	Ntn4
233651	Dchs1	233651	Dchs1	57764	Ntn4	216033	Ctnna3	16776	Lama5
16007	Ccn1	16007	Ccn1	16975	Lrp8	22330	Vcl	76527	Il34
12562	Cdh5	12562	Cdh5	140810	Ttbk2	19283	Ptprz1	230857	Ece1
14102	Fas	14102	Fas	230857	Ece1	18073	Nid1	384569	Nova2
12323	Camk2b	12323	Camk2b	11600	Angpt1	226519	Lamc1	12778	Ackr3

17974	Nck2	11475	Acta2	109700	Itga1	20315	Cxcl12	16779	Lamb2
21390	Tbxa2r	17974	Nck2	18127	Nos3	59308	Emcn	11600	Angpt1
18823	Plp1	21390	Tbxa2r	15530	Hspg2	228788	Ccm2l	109700	Itga1
16009	Igfbp3	18823	Plp1	20681	Sox8	12818	Col14a1	26903	Dysf
11754	Aoc3	16009	Igfbp3	207607	Ccdc40	11606	Agt	14083	Ptk2
20312	Cx3cl1	11754	Aoc3	12389	Cav1	57764	Ntn4	16542	Kdr
17300	Foxc1	20312	Cx3cl1	53614	Reck	11684	Alox12	15530	Hspg2
105450	Mmrn2	17300	Foxc1	12822	Col18a1	12778	Ackr3	14164	Fgf1
93695	Gpnmb	105450	Mmrn2	218877	Sema3g	11600	Angpt1	20361	Sema7a
20672	Sox18	93695	Gpnmb	23805	Apc2	109700	Itga1	22341	Vegfc
240185	Jcad	20672	Sox18	23928	Lamc3	17311	Kitl	19252	Dusp1
20671	Sox17	240185	Jcad	15507	Hspb1	381409	Cdh26	74144	Robo4
18596	Pdgfrb	20671	Sox17	56332	Amotl2	16184	Il2ra	16948	Lox
216033	Ctnna3	68810	Nexn	84004	Mcam	15530	Hspg2	21808	Tgfb2
19283	Ptprz1	18596	Pdgfrb	107449	Unc5b	58187	Cldn10	13636	Efna1
71660	Rarres2	216033	Ctnna3	93742	Pard3	75599	Pcdh1	18845	Plxna2
121021	Cspg4	22330	Vcl	107589	Mylk	74144	Robo4	18208	Ntn1
226519	Lamc1	19283	Ptprz1	319504	Nrcam	170736	Parvb		
20315	Cxcl12	71660	Rarres2	67916	Plpp3	29817	Igfbp7		
11606	Agt	19285	Cavin1	56349	Net1	21417	Zeb1		
57764	Ntn4	121021	Cspg4	11838	Arc	21414	Tcf7		
320736	Vstm4	226519	Lamc1	226251	Ablim1	73173	Pcdh18		
105844	Card10	20315	Cxcl12	76854	Gper1	545370	Hmcn1		
16975	Lrp8	11606	Agt	16600	Klf4	57776	Ttyh1		
140810	Ttbk2	57764	Ntn4	16842	Lef1	13518	Dst		
11684	Alox12	320736	Vstm4	329416	Nostrin	21808	Tgfb2		
12778	Ackr3	105844	Card10	16011	Igfbp5	12389	Cav1		
11600	Angpt1	16975	Lrp8	18795	Plcb1	15896	Icam2		
109700	Itga1	140810	Ttbk2	18791	Plat	380713	Scarf1		
17311	Kitl	11684	Alox12	18552	Pcsk5	12822	Col18a1		
330938	Dixdc1	12778	Ackr3	208177	Phldb2	13636	Efna1		
18127	Nos3	11600	Angpt1	70097	Sash1	14723	Gp1ba		
14164	Fgf1	109700	Itga1	118449	Synpo2	20411	Sorbs1		
20681	Sox8	17311	Kitl	21846	Tie1	434215	Lrrc32		
22341	Vegfc	330938	Dixdc1	98660	Atp1a2	20410	Sorbs3		
20969	Sdc1	18127	Nos3	17909	Myo10	23928	Lamc3		
74144	Robo4	14164	Fgf1	22370	Vtn	69524	Esam		
226751	Cdc42bpa	20681	Sox8	56233	Hdac7	27205	Podxl		
21808	Tgfb2	22341	Vegfc	76527	Il34	170643	Kirrel		
12389	Cav1	20969	Sdc1	384569	Nova2	73182	Pear1		
53614	Reck	74144	Robo4	14083	Ptk2	13846	Ephb4		
12822	Col18a1	13518	Dst	17356	Afdn	15507	Hspb1		
13636	Efna1	226751	Cdc42bpa	353156	Egfl7	84004	Mcam		
14723	Gp1ba	15936	Ier2	17470	Cd200	11540	Adora2a		

218877	Sema3g	207607	Ccdc40	19091	Prkg1	233744	Spon1	
14254	Flt1	21808	Tgfb2	381290	Atp2b4	13602	Sparcl1	
23805	Apc2	12389	Cav1	16948	Lox	211712	Pcdh9	
27205	Podxl	53614	Reck	11815	Apod	93742	Pard3	
19263	Ptprb	12822	Col18a1	16826	Ldb2	68169	Ndnf	
80837	Rhoj	13636	Efna1	18208	Ntn1	17130	Smad6	
13846	Ephb4	14723	Gp1ba	16476	Jun	17131	Smad7	
15507	Hspb1	218877	Sema3g	22352	Vim	18189	Nrxn1	
56332	Amotl2	14254	Flt1	18096	Nkx6-1	22288	Utrn	
84004	Mcam	23805	Apc2	106042	Prickle1	319504	Nrcam	
13609	S1pr1	27205	Podxl	16590	Kit	20668	Sox13	
14257	Flt4	19263	Ptprb	12554	Cdh13	67916	Plpp3	
13601	Ecm1	80837	Rhoj	21380	Tbx1	29875	Iqgap1	
93742	Pard3	13846	Ephb4	12558	Cdh2	57278	Bcam	
68169	Ndnf	15507	Hspb1	20620	Plk2	382111	Susd5	
19279	Ptprr	56332	Amotl2	70445	Cd248	13731	Emp2	
233064	Wdr62	84004	Mcam	545428	Ccdc141	16842	Lef1	
107589	Mylk	13609	S1pr1	66864	Clec14a	16600	Klf4	
29876	Clic4	14257	Flt4	71653	Shtn1	14268	Fn1	
67916	Plpp3	13601	Ecm1	22003	Tpm1	19713	Ret	
13617	Ednra	93742	Pard3	20745	Spock1	56229	Thsd1	
56349	Net1	68169	Ndnf	27494	Amot	16449	Jag1	
13616	Edn3	19279	Ptprr	22240	Dpysl3	50768	Dlc1	
11838	Arc	233064	Wdr62	13653	Egr1	71228	Dlg5	
29875	Iqgap1	107589	Mylk	16007	Ccn1	18795	Plcb1	
76854	Gper1	29876	Clic4	12323	Camk2b	20356	Sema5a	
13731	Emp2	67916	Plpp3	12562	Cdh5	21687	Tek	
16842	Lef1	13617	Ednra	11475	Acta2	242202	Pde5a	
16600	Klf4	56349	Net1	21390	Tbxa2r	22418	Wnt5a	
14268	Fn1	13616	Edn3	16009	Igfbp3	17064	Cd93	
19713	Ret	11838	Arc	17300	Foxc1	19242	Ptn	
239719	Mrtfb	29875	Iqgap1	93695	Gpnmb	16773	Lama2	
13614	Edn1	76854	Gper1	68810	Nexn	16772	Lama1	
494504	Apcdd1	13731	Emp2	216033	Ctnna3	208177	Phldb2	
329416	Nostrin	16842	Lef1	22330	Vcl	18552	Pcsk5	
16449	Jag1	16600	Klf4	19283	Ptprz1	272381	Lrrc4b	
50768	Dlc1	14268	Fn1	71660	Rarres2	17909	Myo10	
71228	Dlg5	19713	Ret	19285	Cavin1	26570	Slc7a11	
18795	Plcb1	239719	Mrtfb	226519	Lamc1	22370	Vtn	
16011	Igfbp5	13614	Edn1	105844	Card10	22371	Vwf	
20356	Sema5a	494504	Apcdd1	11684	Alox12	14115	Fbln2	
21687	Tek	329416	Nostrin	12778	Ackr3	16776	Lama5	
18952	Septin4	16449	Jag1	17311	Kitl	16775	Lama4	
18710	Pik3r3	50768	Dlc1	330938	Dixdc1	226041	Pgm5	

22418	Wnt5a	71228	Dlg5	14164	Fgf1	16779	Lamb2	
18791	Plat	18795	Plcb1	22341	Vegfc	329628	Fat4	
22419	Wnt5b	16011	Igfbp5	20969	Sdc1	94214	Spock2	
19242	Ptn	20356	Sema5a	74144	Robo4	26903	Dysf	
668303	Kif26a	21687	Tek	13518	Dst	14083	Ptk2	
208177	Phldb2	18830	Pltp	226751	Cdc42bpa	17356	Afdn	
18552	Pcsk5	18952	Septin4	15936	Ier2	16542	Kdr	
328949	Mcc	18710	Pik3r3	21808	Tgfb2	353156	Egfl7	
70097	Sash1	22418	Wnt5a	13636	Efna1	268709	Fam107a	
118449	Synpo2	18791	Plat	14723	Gp1ba	19252	Dusp1	
21846	Tie1	22419	Wnt5b	14254	Flt1	17470	Cd200	
20358	Sema6a	19242	Ptn	27205	Podxl	19091	Prkg1	
22370	Vtn	16773	Lama2	19263	Ptprb	225341	Lims2	
56233	Hdac7	16772	Lama1	80837	Rhoj	11899	Astn1	
16776	Lama5	668303	Kif26a	13846	Ephb4	11815	Apod	
76527	Il34	208177	Phldb2	58234	Shank3	77569	Limch1	
16779	Lamb2	18552	Pcsk5	13609	S1pr1	12741	Cldn5	
26903	Dysf	328949	Mcc	14257	Flt4	11898	Ass1	
14083	Ptk2	70097	Sash1	53901	Rcan2	18845	Plxna2	
17356	Afdn	118449	Synpo2	13601	Ecm1	18208	Ntn1	
16542	Kdr	21846	Tie1	68169	Ndnf			
72446	Prr5l	20358	Sema6a	19279	Ptprr			
20361	Sema7a	17909	Myo10	233064	Wdr62			
353156	Egfl7	22370	Vtn	29876	Clic4			
268709	Fam107a	56233	Hdac7	13617	Ednra			
19252	Dusp1	16776	Lama5	13616	Edn3			
17470	Cd200	16775	Lama4	29875	Iqgap1			
319565	Syne2	76527	Il34	13731	Emp2			
19091	Prkg1	16779	Lamb2	14268	Fn1			
381290	Atp2b4	26903	Dysf	239719	Mrtfb			
11899	Astn1	14083	Ptk2	19713	Ret			
16948	Lox	17356	Afdn	13614	Edn1			
11815	Apod	16542	Kdr	494504	Apcdd1			
77569	Limch1	72446	Prr5l	16449	Jag1			
12741	Cldn5	71233	Enkur	50768	Dlc1			
18845	Plxna2	20361	Sema7a	71228	Dlg5			
18208	Ntn1	353156	Egfl7	21687	Tek			
		268709	Fam107a	20356	Sema5a			
		56087	Dnah10	18830	Pltp			
		19252	Dusp1	18952	Septin4			
		17470	Cd200	18710	Pik3r3			
		319565	Syne2	22418	Wnt5a			
		19091	Prkg1	22419	Wnt5b			
		381290	Atp2b4	19242	Ptn			

	11899	Astn1	16773	Lama2		
	16948	Lox	668303	Kif26a		
	11815	Apod	16772	Lama1		
	77569	Limch1	328949	Mcc		
	12741	Cldn5	20358	Sema6a		
	16826	Ldb2	16776	Lama5		
	18845	Plxna2	16775	Lama4		
	18208	Ntn1	16779	Lamb2		
			26903	Dysf		
			16542	Kdr		
			72446	Prr5l		
			71233	Enkur		
			20361	Sema7a		
			268709	Fam107a		
			56087	Dnah10		
			19252	Dusp1		
			319565	Syne2		
			11899	Astn1		
			77569	Limch1		
			12741	Cldn5		
			18845	Plxna2		
